# A systematic review of chronic disease management interventions in primary care

**DOI:** 10.1186/s12875-017-0692-3

**Published:** 2018-01-09

**Authors:** Rebecca Reynolds, Sarah Dennis, Iqbal Hasan, Jan Slewa, Winnie Chen, David Tian, Sangeetha Bobba, Nicholas Zwar

**Affiliations:** 1 UNSW, Sydney, Australia; 2 University of Sydney, Camperdown, Australia; 3 School of Medicine, University of Wollongong, Wollongong, NSW 2522 Australia

**Keywords:** Chronic care, Disease management/care management, Systematic review, Endocrinology, Diabetes, Cardiovascular, Respiratory system, Musculoskeletal/connective tissue, Arthritis

## Abstract

**Background:**

Primary and community care are key settings for the effective management of long term conditions. We aimed to evaluate the pattern of health outcomes in chronic disease management interventions for adults with physical health problems implemented in primary or community care settings.

**Methods:**

The methods were based on our previous review published in 2006. We performed database searches for articles published from 2006 to 2014 and conducted a systematic review with narrative synthesis using the Cochrane Effective Practice and Organisation of Care taxonomy to classify interventions and outcomes. The interventions were mapped to Chronic Care Model elements. The pattern of outcomes related to interventions was summarized by frequency of statistically significant improvements in health care provision and patient outcomes.

**Results:**

A total of 9589 journal articles were retrieved from database searches and snowballing. After screening and verification, 165 articles that detailed 157 studies were included. There were few studies with Health Care Organization (1.9% of studies) or Community Resources (0.6% of studies) as the primary intervention element. Self-Management Support interventions (45.8% of studies) most frequently resulted in improvements in patient–level outcomes. Delivery System Design interventions (22.6% of studies) showed benefits in both professional and patient-level outcomes for a narrow range of conditions. Decision Support interventions (21.3% of studies) had impact limited to professional-level outcomes, in particular use of medications. The small number of studies of Clinical Information System interventions (8.9%) showed benefits for both professional- and patient-level outcomes.

**Conclusions:**

The published literature has expanded substantially since 2006. This review confirms that Self-Management Support is the most frequent Chronic Care Model intervention that is associated with statistically significant improvements, predominately for diabetes and hypertension.

**Electronic supplementary material:**

The online version of this article (10.1186/s12875-017-0692-3) contains supplementary material, which is available to authorized users.

## Background

Chronic disease is defined by the World Health Organization (WHO) as being of long duration, generally slow in progression and not passed from person to person [1]. The Global Burden of Disease study 2013 reported a substantial (42.3%) increase in the years lived with disability (YLD) from 1990 to 2013 [2]. This was overwhelming due to non-communicable diseases, with no infectious diseases in the top 20 leading causes of YLDs globally in 2013. Chronic condition multi-morbidity is high in developed countries [3] and the prevalence of it increases with age; Australian data indicate that around 40% of people aged over 44 years have chronic disease multi-morbidity, increasing to around 50% for 65–74 year olds, and 70% for 85 years or over [4].

Addressing chronic disease is a major challenge for healthcare systems around the world, which have largely developed to deal with acute episodic care, rather than to provide organized care for people with long-term conditions [5]. A characteristic of chronic diseases is that they often require a long period of supervision, observation or care. The defining features of primary care (including continuity, coordination, and comprehensiveness) makes this setting suitable for managing chronic conditions [6]. Evidence increasingly highlights the importance of reorienting health policy and healthcare towards chronic care systems, including primary care that are proactive rather than reactive [7]. Countries with strong primary care systems tend to have better health outcomes at a lower cost [8].

**Fig. 1:**
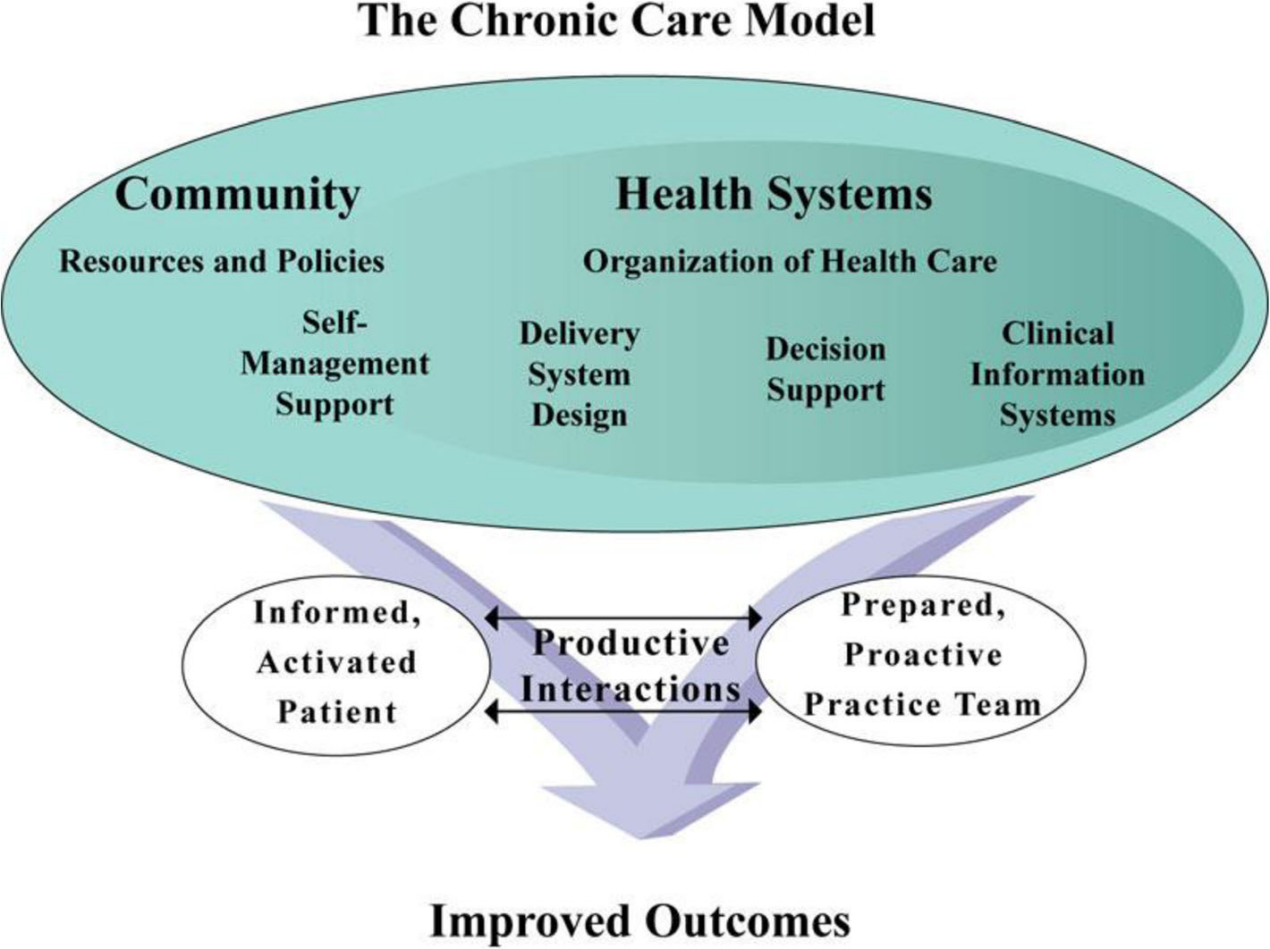
Chronic Care Model

The Chronic Care Model (CCM) was developed in the 1990s by Wagner et al. as a framework to improve the quality of chronic care [9]. It is an organizational approach to caring for people with chronic disease that is particularly applicable in the primary care setting. The six elements of the CCM operate within the context of the individual, community, provider organization and the health care system, see Fig. 1. The model can be used as a guide for system enhancement to provide higher-quality chronic disease management (CDM) [10–12].

Subsequent to development of the CCM there have been a number of other approaches to improving the quality and comprehensiveness of primary care including for the management of chronic diseases. These include the Patient-Centered Medical Home and The Ten Building Blocks of High-Performing Primary Care [13, 14]. Chronic disease management in primary care is an important part of both prevention and treatment of chronic conditions, but there is a need to understand which interventions are effective, for whom and in what context [6]. The literature on interventions to improve CDM in primary care, based on the CCM or otherwise, is diverse and growing. In 2006, we published a systematic review with narrative synthesis of interventions for common physical health problems managed in primary care in developed countries [15, 16]. This review classified interventions under the elements of the CCM, an approach that has subsequently been adopted by other authors [17]. In this article, we provide an updated review and narrative synthesis of the pattern of health outcomes in CDM interventions for physical health problems in the primary care setting to ensure that more recent published evidence is included and used to inform intervention development, policy and practice, as well as guide future research.

## Methods

The methods for the current systematic review with narrative synthesis were based on those used in our previous review [15, 16]. The database searches for the updated review were conducted between 1 January 2006 and 31 December 2014. This systematic review was registered with PROSPERO (CRD42014009219) [18].

### Literature search

Searches were run in four databases: Medline, Embase, PsycINFO and CINAHL. Inclusion and exclusion criteria for articles are described below. The database search terms are shown in Additional file 1.

### Study selection, quality assessment and data extraction

We included studies that were randomized controlled trials (RCTs), controlled clinical trials, controlled before and after and interrupted time series studies; included adults 18 years and older with the chronic physical health conditions of asthma, chronic obstructive pulmonary disease (COPD), type 2 diabetes mellitus (T2DM), heart disease (including heart failure and myocardial infarction), hypertension, lipid disorders, arthritis (osteoarthritis and rheumatoid arthritis) and osteoporosis; were delivered in a primary or community care setting (primary care, including family practice; managed care organizations; community-based but delivered by primary care professionals, including pharmacists) by non-hospital health professionals (including doctors and allied health professionals, nurses and pharmacists) in the following developed countries: Australia, Canada, Denmark, Finland, Iceland, Netherlands, New Zealand, Norway, Sweden, the UK (England, Northern Ireland, Scotland and Wales) and the USA; and the intervention could be mapped to the expanded Effective Practice and Organization of Care (EPOC) taxonomy [19]. Prior systematic reviews were not included.

**Fig. 2:**
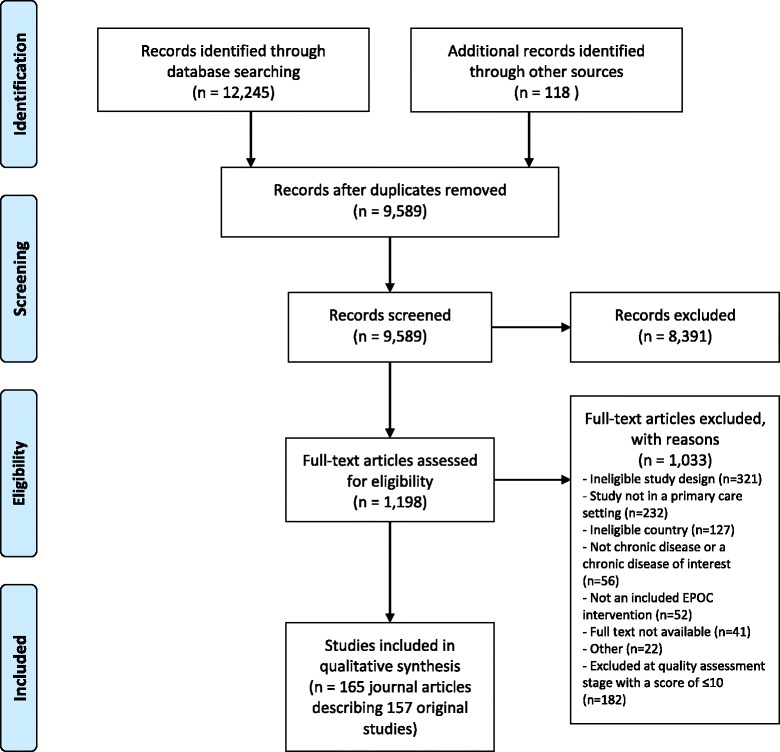
Prisma flow diagram

Articles underwent screening of titles and abstracts; verification of full text and quality assessment used templates (see Additional file 2 and Additional file 3) developed during the previous review [15, 16]. The reference lists of included articles were used in a snowballing process to identify any missed articles. See the Preferred Reporting Items for Systematic Reviews and Meta-Analyses (PRISMA) flow diagram of this assessment process as Fig. 2 [20].

Three reviewers (RR, IH and DT) completed the title/abstract screening process and any discrepancies were reviewed by a fourth reviewer (SD), who also reviewed 20% of excluded articles. No articles were found to have been wrongly excluded. A 20% exclusion check is a validated method to assess the quality of the screening process [21].

Two reviewers (RR and IH) completed the full text verification process and any discrepancies were assessed by a third reviewer (NZ). A 20% check of excluded articles was also carried out at this full text verification stage by one reviewer (SD) and no articles were found that should not have been excluded [21]. The reference lists of all included studies were reviewed by one reviewer (NZ). A further 118 articles were identified which underwent verification and quality assessment.

All articles remaining after full text verification were quality assessed based on study design and other characteristics using the EPOC quality assessment tool [19] by four reviewers (RR, IH, DT and SD). The quality score was out of a total of 14, the included articles had a mean score of 11.1 and a median score of 11.0. Consistent with the methods of the previous review [15, 16], articles with a median score below 11.0 were excluded. The inter-rater reliability for quality scores from the four reviewers was assessed. Each reviewer assessed a 20% sample of papers scored by another reviewer. There was a 1-tailed Pearson correlation coefficient of 0.414 and significance of *p* < 0.01.

Using methods developed for the previous review [15, 16] and also used subsequently by Pasricha et al. [17], data extraction was completed by two reviewers (JS and WC). A data extraction template was developed which included the EPOC Group taxonomy for the classification of the intervention methods [19] and of up to seven categories of study outcomes based on modified methods used by Weingarten et al. 2002 [22]. We expanded the list of outcomes from the previous review [15, 16] by adding change in medication and costs.

Study interventions were coded using the EPOC taxonomy and then mapped to the elements of the CCM (NZ, SD and RR) based on published descriptions of CCM elements [11, 12], with any discrepancies addressed by NZ. Up to three CCM elements (primary, secondary and tertiary) were mapped for each study intervention. Based on the methods used in our previous review [15, 16] and by Weingarten et al. 2002 [22], we used a vote-counting approach to summarize the impact of CCM intervention elements on the primary outcome of the study (as defined by the study authors). Significant and positive (i.e. beneficial) primary and up to six additional outcomes (where present) were recorded at the *p* < 0.05 level. As in our previous review the diversity of studies, types of CCM interventions and outcomes examined prevented the use of meta-analysis to examine effect sizes. The extraction of only the primary outcome and up to six additional outcomes was a modification of the methods from our earlier review where impact of the intervention on all extracted outcomes was examined [15, 16]. Data was analyzed for descriptive statistics using Excel, SPSS (IBM Corp. Released 2013. IBM SPSS Statistics for Windows, Version 22.0. Armonk, NY: IBM Corp) and SAS/STAT® software.

## Results

There were 9589 articles identified after duplicates were removed. After screening and verification 165 journal articles were included in the review, which detailed 157 original studies (some journal articles described different results from the one study design). See Fig. 2 PRISMA flow chart and Additional file 4 for a summary of the characteristics of the included studies.

The majority of the studies were of RCT design (92.9%), allocating the intervention at the patient-level (63.2%), and based in primary care (62.6%). The most common location for studies was the USA (49.0%) and the most common condition targeted was T2DM (43.9%). There were a total of 1,051,707 patients across 100 studies, 2961 health professionals across 14 studies and 7368 practices across 43 studies. Using mean data of participants in the control groups, the mean age was 60.7 years (SD 7.4), with 44.3% male and 23.9% in Caucasian/white populations (when ethnicity was specified). The mean duration of the studies was 14.1 (SD 11.6) months, ranging from 1 to 72 months.

Self-Management Support was the most frequent primary CCM element (*n* = 71, 45.8% of studies) followed by DS (*n* = 35, 22.6%), DSD (*n* = 33, 21.3%) and CIS (*n* = 14, 8.9%). There were relatively few studies with HCO (*n* = 3, 1.9%) or CR (*n* = 1, 0.6%) as the primary intervention element. Table 1 illustrates the CCM elements by disease; T2DM and hypertension were the most frequently studied conditions. Table 2 shows the number of CCM elements by primary outcome. Most study interventions addressed one or two CCM elements. Studies with three elements addressed in the intervention did not, in general, appear to be more effective with respect to statistically significant improvements in outcomes than studies with a smaller number of elements (Table 3).

**Table 1:** Types of Chronic Care Model intervention elements (primary, secondary and tertiary) by disease

Disease	Decision Support (DS)	Delivery System Design (DSD)	Clinical Information Systems (CIS)	Self Management Support (SMS)	Health Care Organization (HCO)	Community Resources (CR)	Total
Type 2 Diabetes	30	38	14	52	2	2	138
Hypertension	21	19	10	24	1	2	77
Heart disease	7	6	6	10	2		31
COPD	5	11	4	11			31
Arthritis	3	5	2	8			18
Osteoporosis	1	1	2	2			6
Asthma	4		1	4			9
Lipid disorders	3	1	1	1			6
Total	73	81	38	112	5	4	313

**Table 2:** Number of Chronic Care Model elements (primary only, *n* = 1; primary and secondary, *n* = 2; or primary, secondary and tertiary, *n* = 3) by primary outcome

OUTCOME CATEGORY													
	Professional-level			Patient-level									Other
Number of CCM elements	Adherence to guidelines	Change in medication	Quality of care	Adherence to treatment	Service use	PMOD	Risk behavior	Quality of life	Health status	Satisfaction	Functional status	Knowledge level	Costs
1	**2 (4)**	**5 (10)**		1 (5)	3 (9)	**11 (23)**	**4 (5)**	**7 (13)**	**6 (9)**	**1 (2)**	3 (7)	**7 (9)**	**1 (2)**
2	**12 (17)**	**12 (15)**	**1 (1)**	**7 (12)**	5 (11)	**34 (55)**	**9 (16)**	8 (18)	2 (12)	**4 (6)**	**9 (15)**	**11 (15)**	**4 (5)**
3	5 (11)	**5 (5)**		1 (5)	**4 (6)**	**20 (30)**	0 (7)	2 (12)	2 (10)	**4 (6)**	0 (3)	**3 (6)**	**4 (4)**

Numbers in cells: number of studies reporting at least one significant outcome (number of studies reporting at least one outcome for that particular category of outcome measure)

Bold font in cells is where 50% or more of studies reported a significant difference for that category of outcome

*CCM* Chronic Care Model, *PMOD* physiological measure of disease

**Table 3:** Combination of elements of Chronic Care Model and outcome measures

Single or combination CCM elements	OUTCOME CATEGORY												
	Professional-level			Patient-level									Other
	Adherence to guidelines	Change in medication	Quality of care	Adherence to treatment	Service use	PMOD	Risk behaviour	Quality of life	Health status	Satisfaction	Functional status	Knowledge level	Costs
DSD overall o Overall	**3 (5)**	**6 (7)**		1 (5)	**4 (7)**	**18(22)**	0 (4)	6 (13)	3 (8)	**4 (5)**	1 (3)	**4 (6)**	**2 (2)**
DSD						0 (1)			**1 (1)**				
DSD + CIS								**1 (1)**	0 (1)				
DSD + CIS + DS				**1 (1)**									
DSD + CIS + SMS					0 (1)	**1 (1)**		0 (1)				0 (1)	
DSD + DS		**2 (2)**				**4 (4)**			**1 (1)**				
DSD + DS + CIS	**1 (1)**					**1 (1)**			**1 (1)**				
DSD + DS + HCO	**1 (1)**									0 (1)			
DSD + DS + SMS	0 (1)	**1 (1)**			**1 (1)**	**1 (2)**	0 (1)	0 (1)				**1 (1)**	
DSD + SMS		**2 (3)**		0 (2)	**2 (3)**	**4 (5)**	0 (1)	**3 (5)**	0 (1)	**2 (2)**	**1 (2)**	**3 (3)**	
DSD + SMS + CIS	0 (1)			0 (1)	0 (1)	**1 (1)**		0 (2)	0 (1)	**1 (1)**			**1 (1)**
DSD + SMS + DS	**1 (1)**	**1 (1)**		0 (1)	**1 (1)**	**6 (7)**	0 (2)	**2 (3)**	0 (2)	**1 (1)**	0 (1)	0 (1)	**1 (1)**
DS overall	6 (13)	**8 (13)**		1 (3)	**5 (9)**	8 (22)	**2 (3)**	1 (5)	2 (9)	1 (3)	2 (10)	**1 (1)**	**1 (2)**
DS	**2 (3)**	3 (8)		**1 (2)**	**3 (6)**	3 (7)		1 (3)	**2 (2)**	0 (1)	**2 (4)**		0 (1)
DS + CIS	1 (3)	**2 (2)**				0 (3)	**1 (1)**		0 (1)		0 (1)		**1 (1)**
DS + CIS + DSD	**1 (1)**								0 (1)		0 (1)		
DS + CIS + SMS	0 (1)												
DS + DSD	0 (1)	**2 (2)**		0 (1)		**3 (4)**			0 (1)	0 (1)	0 (1)		
DS + DSD + SMS	0 (1)				**1 (1)**	1 (4)		0 (2)	0 (3)	**1 (1)**	0 (1)		
DS + HCO	**1 (1)**										0 (1)		
DS + SMS	**1 (2)**	**1 (1)**			**1 (2)**	1 (4)	**1 (2)**		0 (1)		0 (1)	**1 (1)**	
CIS overall	**4 (5)**	**2 (2)**	**1 (1)**		**1 (2)**	**5 (8)**	0 (1)			**1 (1)**	**4 (5)**		**1 (2)**
CIS + DS	**4 (4)**	**1 (1)**	**1 (1)**		**1 (1)**	1 (3)	0 (1)			**1 (1)**	**4 (4)**		**1 (1)**
CIS + DSD						**1 (1)**							
CIS + SMS	0 (1)				0 (1)	**1 (2)**					0 (1)		0 (1)
CIS + SMS + DSD		**1 (1)**				**2 (2)**							
SMS overall	**5 (7)**	**5 (6)**		6 (13)	1 (7)	**32(52**)	**11 (20)**	**10(25)**	5 (13)	**3 (5)**	**5 (5)**	**16 (23)**	**5 (5)**
SMS		**1 (1)**		0 (3)	0 (3)	8 (14)	**4 (5)**	**6 (10)**	**3 (5)**		**1 (2)**	**7 (9)**	**1 (1)**
SMS + CIS	**1 (1)**			**2 (2)**	0 (1)	2 (5)	**1 (1)**	0 (2)	0 (1)	**1 (1)**	**1 (1)**	**1 (1)**	
SMS + CIS + DSD		**1 (1)**				**1 (1)**	0 (1)						
SMS + CR				**1 (1)**		**1 (1)**							
SMS + CR + DSD						0 (1)							
SMS + DS	**1 (1)**	**1 (1)**			0 (1)	**5 (6)**	**2 (2)**	**1 (2)**				**1 (1)**	**1 (1)**
SMS + DS + CIS	**1 (1)**	**1 (1)**				**2 (2)**		0 (1)	0 (1)				**2 (2)**
SMS + DS + DSD	0 (1)				**1 (1)**	0 (1)	0 (1)			0 (1)			
SMS + DSD	**2 (2)**	**1 (2)**		**3 (5)**	0 (1)	**9 (14)**	**4 (8)**	**3 (8)**	1 (5)	**1 (2)**	**3 (3)**	**5 (9)**	**1 (1)**
SMS + DSD + CIS						1 (1)		0 (1)	**1 (1)**	**1 (1)**		0 (1)	
SMS + DSD + CR						**1 (1)**						**1 (1)**	
SMS + DSD + DS	0 (1)			0 (2)		2 (5)	0 (2)	0 (1)				**1 (1)**	
HCO + CIS	**1 (1)**					**1 (1)**							
HCO + DSD				**1 (1)**		0 (1)							
CR + DSD		0 (1)			**1 (1)**	**1 (1)**							

Numbers in cells: number of studies reporting at least one significant outcome (number of studies reporting at least one outcome)

Bold font in cells is where 50% or more of studies reported a significant difference for that category of outcome

*CCM* Chronic Care Model, *CIS* clinical information systems, *COPD* chronic obstructive pulmonary disease, *CR* community resources, *DS* decision support, *DSD* delivery system design, *HCO* health care organization, *PMOD* physiological measure of disease, *SMS* self-management support

The exception to this was where the primary element was a DSD change accompanied by SMS and DS intervention elements. Table 4 illustrates the proportion of studies with a significant result for professional or patient-level outcome measures (primary outcome and up to six additional outcomes) for each primary CCM intervention element overall and by disease. Self-Management Support interventions resulted in improvements in patient-level outcomes; such as physiological measures of disease, risk behavior, satisfaction and knowledge in more than half the studies analyzed. Delivery System Design interventions showed benefits in both professional and patient level outcomes but this was confined to a narrow range of conditions. Decision Support interventions tended to have impact limited to professional-level outcomes, in particular use of medications. There were small numbers of studies of Clinical Information System interventions as the primary element but the results show benefits for both professional and patient level outcomes.

**Table 4:** Elements of Chronic Care Model and positive outcome measures by disease

Primary CCM element	Disease	OUTCOME CATEGORY												
		Professional			Patient									Other
		Adherence to guidelines	Change in medication	Quality of care	Adherence to treatment	Service use	PMOD	Risk behaviour	Quality of life	Health status	Satisfaction	Functional status	Knowledge level	Costs
DSD	Overall	**3 (5)**	**6 (7)**		1 (5)	**4 (7)**	**18 (22)**	0 (4)	6 (13)	3 (8)	**4 (5)**	1 (3)	**4 (6)**	**2 (2)**
	Arthritis								**1 (2)**	0 (1)		**1 (1)**		
	Asthma													
	COPD	0 (1)				1 (4)	0 (1)	0 (1)	1 (5)	0 (1)			**2 (3)**	
	Diabetes	**3 (3)**	**3 (4)**		0 (2)	**2 (2)**	**12 (14)**	0 (2)	**4 (4)**	**1 (2)**	**3 (4)**	0 (1)	**2 (2)**	**1 (1)**
	Heart disease	0 (1)			0 (2)		0 (1)	0 (1)	0 (1)	1 (3)	**1 (1)**			**1 (1)**
	Hypertension		**3 (3)**			**1 (1)**	**5 (5)**		0 (1)	1 (1)		0 (1)	0 (1)	
	Lipid disorders						**1 (1)**							
	Osteoporosis				**1 (1)**									
DS	Overall	6 (13)	**8 (13)**		1 (3)	**5 (9)**	8 (22)	**2 (3)**	1 (5)	2 (9)	1 (3)	2 (10)	**1 (1)**	**1 (2)**
	Arthritis													
	Asthma	**1 (1)**	**1 (2)**		**1 (2)**	0 (1)	0 (1)		1 (3)	**1 (1)**				
	COPD					0 (1)			0 (1)	**1 (2)**	**1 (1)**	**1 (2)**		
	Diabetes	1 (3)	**3 (4)**			**2 (3)**	4 (11)	**2 (2)**	0 (1)	0 (3)	0 (2)	0 (2)	**1 (1)**	**1 (2)**
	Heart disease	1 (3)	**2 (3)**			**1 (2)**	0 (3)	0 (1)		0 (1)		0 (2)		
	Hypertension	**2 (4)**	**2 (4)**		0 (1)	**2 (2)**	**4 (7)**			0 (2)		1 (3)		
	Lipid disorders	**1 (2)**										0 (1)		
	Osteoporosis													
CIS	Overall	**4 (5)**	**3 (3)**	**1 (1)**		**1 (2)**	**5 (8)**	0 (1)			**1 (1)**	**4 (5)**		**1 (2)**
	Arthritis													
	Asthma	**1 (1)**		**1 (1)**								**1 (1)**		
	COPD													
	Diabetes	**2 (3)**				**1 (1)**	**2 (3)**				**1 (1)**	**2 (3)**		**1 (1)**
	Heart disease		**1 (1)**			0 (1)	**1 (1)**							
	Hypertension	**1 (1)**	**2 (2)**				**2 (4)**	0 (1)				**1 (1)**		0 (1)
	Lipid disorders													
	Osteoporosis													
SMS	Overall	**5 (7)**	**5 (6)**		6 (13)	1 (7)	**32 (52)**	**11 (20)**	10 (25)	5 (13)	**3 (5)**	**5 (6)**	**16 (23)**	**5 (5)**
	Arthritis	**1 (1)**					**1 (1)**		**3 (5)**			**2 (2)**	**2 (3)**	
	Asthma		**1 (1)**			0 (2)	**1 (2)**		1 (3)	**1 (2)**				
	COPD		0 (1)			**1 (2)**		**1 (1)**	**2 (4)**	**1 (1)**	**1 (2)**	**1 (1)**	**3 (5)**	
	Diabetes	**1 (1)**			**4 (8)**	0 (1)	**17 (29)**	**9 (15)**	3 (8)	2 (6)	**1 (2)**	**1 (2)**	**11 (14)**	**1 (1)**
	Heart disease		**1 (1)**			0 (2)	2 (5)		**1 (2)**	1 (3)	**1 (1)**			**1 (1)**
	Hypertension	**3 (5)**	**3 (3)**		1 (3)		**11 (15)**	1 (4)	0 (3)	0 (1)		**1 (1)**	0 (1)	**3 (3)**
	Lipid disorders													
	Osteoporosis				**1 (2)**		**1 (1)**		0 (1)	**1 (1)**				
HCO	Overall	**1 (2)**			1 (1)		1 (3)			0 (1)		0 (1)		
	Arthritis													
	Asthma													
	COPD													
	Diabetes				1 (1)		0 (1)							
	Heart disease	**1 (1)**					**1 (1)**							
	Hypertension	0 (1)					0 (1)			0 (1)		0 (1)		
	Lipid disorders													
	Osteoporosis													
CR	Overall		0 (1)			**1 (1)**	**1 (1)**							
	Arthritis													
	Asthma													
	COPD													
	Diabetes													
	Heart disease													
	Hypertension		0 (1)			**1 (1)**	**1 (1)**							
	Lipid disorders													
	Osteoporosis													

Numbers in cells: number of studies reporting at least one significant outcome (number of studies reporting at least one outcome)

Bold font in cells is where 50% or more of studies reported a significant difference for that category of outcome

*CCM* Chronic Care Model, *CIS* clinical information systems, *COPD* chronic obstructive pulmonary disease, *CR* community resources, *DS* decision support, *DSD* delivery system design, *HCO* health care organization, *PMOD* physiological measure of disease, *SMS* self-management support

## Discussion

Since publication of the 2006 review [15, 16], the number of potentially eligible studies has increased considerably (9589 studies screened between 2006 and 2014 compared to 5160 studies between 1990 and 2006). This likely reflects a growing interest in how to meet the challenge of the increasing prevalence and burden of chronic disease to health systems globally. Consistent with the 2006 review [15, 16], this review showed that SMS was the most commonly tested intervention with the greatest proportion of studies demonstrating a significant result for one or more outcome measure categories for all diseases examined in the review, with the exception of lipid disorders. The effect of SMS was most often to improve physiological measures of disease in patients with T2DM and hypertension, and improve patient knowledge in T2DM and COPD. There was also some evidence of benefit on quality of life for patients with arthritis and COPD. Studies with SMS interventions as the primary element less often examined the impact of the intervention on health professional behavior.

Although the number of studies was small, CIS interventions showed benefit on both professional and patient level outcomes in particular for T2DM and hypertension. This is in contrast to our 2006 review findings and may indicate increasing sophistication of CIS interventions with computerized recall and reminder systems, feedback to clinicians and access to computerized DS tools [23]. The next most common CCM element as a primary intervention that was associated with statistically significant improvements in outcomes was DSD, which benefited physiological measures of disease control and health professional behavior in patients with T2DM diabetes and hypertension, but had limited or no impact in other conditions. The other CCM element showing worthwhile evidence of benefit was DS which had some impact on health professional behavior including changing medication and adherence to guidelines but had limited impact on patient-level measures of disease control.

In contrast to the earlier 2006 review [15, 16], this review suggested that two CCM elements were more likely to result in significant improvements in outcomes compared to one or three, whereas the previous review found that adding multiple elements to the intervention programs did not produce better outcomes. The combination of SMS and DSD interventions was the most frequent combination of elements associated with significant improvements in outcomes, followed by SMS and DS. The results for the SMS intervention on professional and patient outcomes are consistent with those from 2006. Results for DS suggest reduced impact of the interventions on health professionals’ adherence to guidelines, which was unexpected. This could be explained by increasing sophistication of CIS where DS interventions are now more frequently embedded.

### Comparisons to other studies

A number of other reviews have been conducted that examined the impact of interventions based on CCM elements. Using methods for categorizing and mapping interventions based on our 2006 publication, Pasricha et al. [17] examined the effectiveness of DS and CIS interventions on improving the care of people living with HIV. They found evidence of modest improvements, with greater impact on process measures compared to outcome measures. This is consistent with our findings for DS and CIS interventions.

Baptista et al. [24] conducted a systematic review of studies which evaluated interventions based on CCM elements for T2DM. That review was restricted to RCTs with at least 3 month’s follow-up and assessed only primary clinical outcomes (mortality) or intermediate clinical outcomes (glycosylated haemoglobin). They identified 12 studies that met the inclusion criteria. In six of these there was evidence of improvement of clinical outcomes. Baptista et al. [24] concluded that interventions based on isolated components of CCM may not be enough to improve clinical outcomes and suggested that greater benefits could be obtained through interventions combining the CCM’s six elements. In our review, studies with three or more CCM elements in the intervention were not associated with more statistically significant improvements in outcomes than studies with a smaller number of elements. The differences in findings and conclusions may be explained by the wider inclusion criteria for this review and the broader range of outcomes examined.

### Strengths and limitations of this review

The strengths of this systematic review include: the use of pre-published methods [15, 16]; the large number of articles and studies summarized; and the mapping of study interventions to the CCM, a method that is becoming increasingly common [17, 24, 25]. The limitations of this review include the exclusion of studies from developing countries. The countries included in the 2006 review were chosen according to health care systems broadly comparable to Australia, and for comparison the same countries were included in this study. Widening the inclusion criteria would have increased the number of eligible studies and may have been advantageous given that health systems in low and middle income countries are similarly challenged with increasing prevalence of chronic disease. As in our previous review, the scope of chronic disease included did not include cancer or mental health problems. The study duration of included studies ranged from 1 to 72 months and therefore information on long-term impacts is limited. A further limitation was that on this occasion we did not examine the impact of CCM interventions on all the outcomes extracted, but only on the primary outcome (as defined by the study authors) and up to six additional outcomes. This may be a factor in some of the differences observed since the previous review. A strength of our approach is that it provides a high-level overview of the pattern of effectiveness of CCM interventions on a range of outcomes across a number of chronic conditions however our approach makes it difficult to drill down to the impact of individual interventions for individual conditions. and to see the effect size of an intervention on a single outcome measure. Lastly, using *P*-values to indicate which interventions likely resulted in improvements in chronic disease outcomes has drawbacks, including not separating the estimated effect size and the estimated precision of the measure.

## Conclusions

This review demonstrated benefits from implementation of interventions based on CCM elements in primary care. The findings provide further evidence to support the view that self-management education should be an integral part of high-quality primary care [26]. Kadu and Stolle [27] have identified that characteristics of the health care organization and the needs and capacity of health care providers are important influences on implementing the CCM model in primary care. There remains a paucity of research on interventions which aim to address the HCO element of the CCM and its impact on process and patient outcomes. The need for further research in this area is highlighted by both our finding and reviews by Kadu and Stolle [27] and Dauvrin et al. [28] who suggested that the scope of chronic care interventions should be expanded to transform health care organizations and systems. There is also a need for research on CR interventions, particularly given the principles of health promotion to involve communities and the recognition of the importance of environmental factors on health, as emphasized by Barr et al. in their description of an expanded CCM [29].

## Additional files

Additional file 1: Database search terms. (DOCX 24 kb)

Additional file 2: Full text verification template. (DOCX 27 kb)

Additional file 3: Quality assessment template. (DOCX 29 kb)

Additional file 4: Summary of the 165 publications included in the review. (DOCX 139 kb)

## Abbreviations

CCM: Chronic care model
CDM: Chronic disease management
CIS: Clinical information systems
COPD: Chronic obstructive pulmonary disease
CR: Community resources
DS: Decision support
DSD: Delivery system design
EPOC: Effective practice and organisation of care
PRISMA: Preferred reporting items for systematic reviews and meta-analyses
RCT: Randomized controlled trial
SD: Standard devisation
SMS: Self-management support
T2DM: Type 2 diabetes mellitus
WHO: World Health Organization
YLD: Years lived with disability
