# Supplementary material for: A systematic review of chronic disease management interventions in primary care
Source: BMC Fam Pract. 2018 Jan 9;19:11. doi: 10.1186/s12875-017-0692-3 (PMC5759778; doi:10.1186/s12875-017-0692-3)
Supplement: Supplementary file 1 — Database search terms. (DOCX 24 kb) [file 12875_2017_692_MOESM1_ESM.docx]

**Appendix 1. Database search terms**

**EMBASE**

1. exp chronic disease/

2. exp asthma/

3. exp chronic obstructive lung disease/

4. exp diabetes mellitus/

5. exp heart disease/ or exp hypertension/

6. exp hyperlipidemia/

7. exp arthritis/

8. exp osteoporosis/

9. 1 or 2 or 3 or 4 or 5 or 6 or 7 or 8

10. exp Ambulatory Care/

11. exp patient care/

12. exp ambulatory monitoring/ or exp blood glucose monitoring/ or exp blood pressure monitoring/ or exp home monitoring/ or exp self monitoring/

13. exp disease management/

14. exp long term care/

15. exp self care/

16. exp patient compliance/ or exp patient participation/

17. exp health promotion/ or exp patient education/

18. exp doctor nurse relation/ or exp doctor patient relation/ or exp medical decision making/

19. exp information system/

20. exp register/

21. exp "health care cost"/ or exp reimbursement/

22. health care quality/ or exp practice guideline/ or exp outcomes research/

23. (care adj (plan$ or team$)).tw.

24. (share$ adj care).tw.

25. (self adj (monitor$ or manage$)).tw.

26. (disease$ adj register$).tw.

27. ((patient$ or pract$) adj guideline$).tw.

28. (recall adj2 system$).tw.

29. (integrat$ adj2 (care or service)).tw.

30. ((effect? or impact or evaluat$ or introduc$ or compare$) adj2 care program$).tw.

31. ((introduc$ or impact or effect? or implement$ or computer$) adj2 protocol?).tw.

32. ((effect? or impact or evaluat$ or introduc$ or compara$) adj2 prevent$ program$).tw.

33. community matron$.tw.

34. (step$ adj care).tw.

35. (disease adj manag$).tw.

36. 10 or 11 or 12 or 13 or 14 or 15 or 16 or 17 or 18 or 19 or 20 or 21 or 22 or 23 or 24 or 25 or 26 or 27 or 28 or 29 or 30 or 31 or 32 or 33 or 34 or 35

37. 9 and 36

38. exp general practice/ or exp primary medical care/

39. exp general practitioner/

40. (general adj2 practi$).tw.

41. (gps or gp).tw.

42. (family adj2 physician$).tw.

43. exp primary health care/

44. (family adj2 doctor$).tw.

45. (family adj2 pract$).tw.

46. (primary adj2 care).tw.

47. primary health.tw.

48. family medicine.tw.

49. 38 or 39 or 40 or 41 or 42 or 43 or 44 or 45 or 46 or 47 or 48

50. 37 and 49

51. randomized controlled trial/

52. (randomised or randomized).tw.

53. experiment$.tw.

54. (time adj series).tw.

55. (pre test or pretest or post test or posttest).tw.

56. impact.tw.

57. intervention?.tw.

58. chang$.tw.

59. evaluat$.tw.

60. effect?.tw.

61. compar$.tw.

62. 51 or 52 or 53 or 54 or 55 or 56 or 57 or 58 or 59 or 60 or 61

63. nonhuman/

64. 62 not 63

65. 50 and 64

66. exp "arctic and antarctic"/ or exp africa/ or exp asia/ or exp oceanic regions/ or exp mexico/ or exp "south and central america"/

67. exp eastern europe/ or exp baltic states/

68. 66 or 67

69. 65 not 68

70. exp screening/

71. 69 not 70

72. limit 71 to (human and english language and yr="2006 - 2012" and (adult <18 to 64 years> or aged <65+ years>))

73. exp prevention/

74. 72 not 73

**MEDLINE**

1. exp Coronary Disease/

2. Asthma/

3. exp Pulmonary Disease, Chronic Obstructive/

4. exp Diabetes Mellitus, Type 2/

5. exp arthritis, rheumatoid/ or exp osteoarthritis/

6. exp Hypertension/

7. exp Hyperlipidemias/

8. exp Osteoporosis/

9. exp Chronic Disease/

10. 1 or 2 or 3 or 4 or 5 or 6 or 7 or 8 or 9

11. exp Ambulatory Care/

12. exp Patient Care Management/

13. exp Patient Care Planning/

14. exp Patient Care Team/

15. exp "Continuity of Patient Care"/

16. exp Disease Management/

17. exp Comprehensive Health Care/

18. exp GUIDELINE/ or exp PRACTICE GUIDELINE/

19. exp Self Care/

20. exp Motivation/

21. exp Patient Participation/

22. exp patient education as topic/

23. exp Reminder Systems/

24. exp Information Systems/

25. exp Decision Support Systems, Clinical/

26. exp Decision Making, Computer-Assisted/

27. exp Management Information Systems/

28. exp Ambulatory Care Information Systems/

29. exp "Quality of Health Care"/

30. exp REIMBURSEMENT, INCENTIVE/

31. exp Registries/

32. (self adj (monitor$ or manage$)).tw.

33. (care adj (plan$ or team$)).tw.

34. (share$ adj care).tw.

35. (disease$ adj register$).tw.

36. ((patient$ or practic$) adj guideline$).tw.

37. (recall adj2 system$).tw.

38. (integrat$ adj2 (care or service)).tw.

39. ((effect? or impact or evaluat$ or introduc$ or compare$) adj2 care program$).tw.

40. ((effect? or impact or evaluat$ or introduc$ or compara$) adj2 prevent$ program$).tw.

41. ((introduc$ or impact or effect? or implement$ or computer$) adj2 protocol?).tw.

42. community matron$.tw.

43. (step$ adj care).tw.

44. (disease adj manag$).tw.

45. 11 or 12 or 13 or 14 or 15 or 16 or 17 or 18 or 19 or 20 or 21 or 22 or 23 or 24 or 25 or 26 or 27 or 28 or 29 or 30 or 31 or 32 or 33 or 34 or 35 or 36 or 37 or 38 or 39 or 40 or 41 or 42 or 43 or 44

46. 10 and 45

47. exp Primary Health Care/

48. exp Comprehensive Health Care/

49. exp Patient Care Management/

50. exp Family Practice/

51. exp Physicians, Family/

52. exp Community Health Services/

53. (primary adj1 (care or health)).tw.

54. (family adj1 (doct$ or medic$ or pract$ or physic$)).tw.

55. (general adj1 practi$).tw.

56. (gps or gp).tw.

57. 47 or 48 or 49 or 50 or 51 or 52 or 53 or 54 or 55 or 56

58. 46 and 57

59. randomized controlled trial.pt.

60. controlled clinical trial.pt.

61. intervention studies/

62. experiment$.tw.

63. (time adj1 series).tw.

64. random allocation/

65. impact.tw.

66. intervention?.tw.

67. change$.tw.

68. evaluation studies/

69. evaluat$.tw.

70. effect?.tw.

71. comparative studies/

72. animal/

73. human/

74. 72 not 73

75. 59 or 60 or 61 or 62 or 63 or 64 or 65 or 66 or 67 or 68 or 69 or 70 or 71

76. 75 not 74

77. 58 and 76

78. exp africa/ or exp caribbean region/ or exp central america/ or exp latin america/ or exp greenland/ or exp mexico/ or exp south america/ or exp antarctic regions/ or exp arctic regions/ or exp asia/ or exp atlantic islands/ or exp indian ocean islands/ or exp oceania/ or exp "oceans and seas"/ or exp melanesia/ or exp micronesia/ or exp polynesia/

79. 77 not 78

80. limit 79 to (english language and humans and yr="2006 - 2012" and "all adult (19 plus years)")

81. exp Acute Disease/

82. 80 not 81

83. "Retrospective Studies"/

84. 82 not 83

85. exp Mass Screening/

86. 84 not 85

87. exp clinical trial, phase i/ [publication type]

88. exp clinical trial, phase ii/ [publication type]

89. exp clinical trial, phase iii/ [publication type]

90. exp clinical trial, phase iv/ [publication type]

91. 87 or 88 or 89 or 90

92. 86 not 91

93. exp Neoplasms/

94. 92 not 93

95. exp review/ [publication type]

96. 94 not 95

**PsycINFO**

1. exp asthma/ or exp chronic obstructive pulmonary disease/

2. exp heart disorders/

3. exp hypertension/

4. exp arthritis/ or exp rheumatoid arthritis/

5. diabetes/ or exp diabetes mellitus/

6. exp chronic illness/

7. 1 or 2 or 3 or 4 or 5 or 6

8. exp "quality of care"/ or exp "continuum of care"/ or exp health care delivery/ or exp health care services/ or exp managed care/

9. exp interdisciplinary treatment approach/ or exp integrated services/ or exp multimodal treatment approach/

10. exp self monitoring/ or exp monitoring/ or exp self management/

11. exp treatment planning/ or exp case management/ or exp health care delivery/ or exp managed care/ or exp treatment guidelines/

12. exp decision making/ or exp decision support systems/

13. exp health care utilization/ or exp health care ulitisation/

14. exp client education/

15. (self adj (monitor or manage$)).tw.

16. (care adj (plan$ or team$)).tw.

17. (share$ adj care).tw.

18. (disease adj register$).tw.

19. ((patient$ or practic$) adj guideline$).tw.

20. (recall adj2 system$).tw.

21. (integrat$ adj2 (care or service)).tw.

22. ((effect? or impact or evaluat$ or introduc$ or compare$) adj2 care program$).tw.

23. ((effect? or impact or evaluat$ or introduc$ or compara$) adj2 prevent$ program$).tw.

24. ((introduc$ or impact or effect? or implement$ or computer$) adj2 protocol?).tw.

25. community matron.tw.

26. (step$ adj care).tw.

27. (disease adj manag$).tw.

28. 8 or 9 or 10 or 11 or 12 or 13 or 14 or 15 or 16 or 17 or 18 or 19 or 20 or 21 or 22 or 23 or 24 or 25 or 26 or 27

29. 7 and 28

30. exp primary health care/ or exp health care delivery/ or exp integrated services/ or exp managed care/

31. exp family medicine/ or exp family physicians/ or exp general practitioners/

32. community services/ or exp health care services/

33. (primary adj1 (care or health)).tw.

34. (family adj1 (doct$ or medic$ or pract$ or physic$)).tw.

35. (general adj1 practi$).tw.

36. (gps or gp).tw.

37. 30 or 31 or 32 or 33 or 34 or 35 or 36

38. 29 and 37

39. experimental design/ or exp clinical trials/ or exp followup studies/ or exp quasi experimental methods/

40. experiment$.tw.

41. (time adj1 series).tw.

42. impact.tw.

43. intervention?.tw.

44. change$.tw.

45. evaluat$.tw.

46. effect?.tw.

47. exp ANIMALS/

48. 39 or 40 or 41 or 42 or 43 or 44 or 45 or 46

49. 48 not 47

50. 38 and 49

51. exp PAIN/

52. 50 not 51

53. limit 52 to (peer reviewed journal and human and english language and "300 adulthood " and yr="2006 - 2012")

**CINAHL**

| S66 | s64 not s65 | Search modes - Boolean/Phrase |
| --- | --- | --- |
| S65 | africa or antarctic regions or arctic regions or asia or atlantic islands or andorra or armenia or austria or azerbaijan or belgium or europe, eastern or france or "georgia (republic)" or germany or gibraltar or greece or italy or liechtenstein or luxembourg or mediterranean region or monaco or portugal or san marino or spain or switzerland or indian ocean island | Search modes - Boolean/Phrase |
| S64 | s53 and s62 | Limiters - Date Published from: 20060101-20121231; English Language; Human; Age Groups: Adult: 19-44 years, Middle Aged: 45-64 years, Aged: 65+ years, Aged, 80 and over, All Adult  Search modes - Boolean/Phrase |
| S63 | s53 and s62 | Search modes - Boolean/Phrase |
| S62 | S54 OR S55 OR S56 OR S57 OR S58 OR S59 OR S60 OR S61 | Search modes - Boolean/Phrase |
| S61 | time series OR experiment$ OR impact OR intervention? OR evaluate$ OR effect$ | Search modes - Boolean/Phrase |
| S60 | (MH "Comparative Studies") | Search modes - Boolean/Phrase |
| S59 | (MH "Quasi-Experimental Studies+") | Search modes - Boolean/Phrase |
| S58 | (MH "Pretest-Posttest Design") OR (MH "Pretest-Posttest Control Group Design") | Search modes - Boolean/Phrase |
| S57 | (random$ adj1 (allocat$ or assign$)) | Search modes - SmartText Searching |
| S56 | (randomised or randomized) | Search modes - Boolean/Phrase |
| S55 | (controlled adj (study or trial)) | Search modes - SmartText Searching |
| S54 | (MH "Clinical Trials") OR (MH "Randomized Controlled Trials") | Search modes - Boolean/Phrase |
| S53 | s43 and s52 | Search modes - Boolean/Phrase |
| S52 | s44 or s45 or s46 or s47 or s48 or s49 or s50 or s51 | Search modes - Boolean/Phrase |
| S51 | family medicine | Search modes - Boolean/Phrase |
| S50 | (primary adj2 care) OR (primary adj2 practi$) OR primary health | Search modes - Boolean/Phrase |
| S49 | (MH "Primary Health Care") | Search modes - Boolean/Phrase |
| S48 | (family adj2 physician$) | Search modes - SmartText Searching |
| S47 | (MH "Physicians, Family") | Search modes - Boolean/Phrase |
| S46 | (gps or gp) | Search modes - Boolean/Phrase |
| S45 | (general adj2 practic$) | Search modes - SmartText Searching |
| S44 | (MH "Family Practice") | Search modes - Boolean/Phrase |
| S43 | s11 and s42 | Search modes - Boolean/Phrase |
| S42 | S12 OR S13 OR S14 OR S15 OR S16 OR S17 OR S18 OR S19 OR S20 OR S21 OR S22 OR S23 OR S24 OR S25 OR S26 OR S27 OR S28 OR S29 OR S30 OR S31 OR S32 OR S33 OR S34 OR S35 OR S36 OR S37 OR S38 OR S39 OR S40 OR S41 | Search modes - Boolean/Phrase |
| S41 | (disease adj manage$) | Search modes - SmartText Searching |
| S40 | (step$ adj care) | Search modes - SmartText Searching |
| S39 | (collaborativ$ adj care) | Search modes - SmartText Searching |
| S38 | community matron | Search modes - Boolean/Phrase |
| S37 | (introduc$ adj2 protocol?) OR (impact adj2 protocol?) OR (effect? adj2 protocol?) OR (implement$ adj2 protocol?) OR (computer$ adj2 protocol?) | Search modes - SmartText Searching |
| S36 | (effect? adj2 prevent$ program$) OR (impact adj2 prevent$ program$) OR (evaluat$ adj2 prevent$ program$) OR (introduc$ adj2 prevent$ program$) OR (compar$ adj2 prevent$ program$) | Search modes - SmartText Searching |
| S35 | (effect? adj2 care program$) OR (impact adj2 care program$) OR (evaluat$ adj2 care program$) OR (introduc$ adj2 care program$) OR (compar$ adj2 care program$) | Search modes - SmartText Searching |
| S34 | (integrate$ adj2 service) OR (integrate$ adj2 care) | Search modes - SmartText Searching |
| S33 | (integrate$ adj2 service) | Search modes - SmartText Searching |
| S32 | (share$ adj care) | Search modes - SmartText Searching |
| S31 | (recall adj2 system$) | Search modes - SmartText Searching |
| S30 | ((patient$ or practic$) adj guideline$) | Search modes - SmartText Searching |
| S29 | (disease adj register$) | Search modes - SmartText Searching |
| S28 | (care adj (plan$ or team$)) | Search modes - SmartText Searching |
| S27 | (self adj (monitor or manage$)) | Search modes - SmartText Searching |
| S26 | (MH "Motivation+") OR (MH "Motivational Interviewing") | Search modes - Boolean/Phrase |
| S25 | (MH "Consumer Participation") | Search modes - Boolean/Phrase |
| S24 | (MH "Quality of Health Care+") | Search modes - Boolean/Phrase |
| S23 | (MH "Reminder Systems") OR (MH "Decision Support Systems, Clinical") OR (MH "Clinical Information Systems+") | Search modes - Boolean/Phrase |
| S22 | (MH "Patient Education+") OR (MH "Diabetes Education") | Search modes - Boolean/Phrase |
| S21 | (MH "Practice Guidelines") | Search modes - Boolean/Phrase |
| S20 | (MH "Managed Care Programs+") OR (MH "Managed Care Information Systems+") OR (MH "Self Care+") | Search modes - Boolean/Phrase |
| S19 | (MH "Protocols+") | Search modes - Boolean/Phrase |
| S18 | (MH "Primary Health Care") | Search modes - Boolean/Phrase |
| S17 | (MH "Patient Centered Care") | Search modes - Boolean/Phrase |
| S16 | (MH "Continuity of Patient Care+") OR (MH "Patient Care Conferences+") OR (MH "Patient Care Plans+") | Search modes - Boolean/Phrase |
| S15 | (MH "Case Management") OR (MH "Disease Management") | Search modes - Boolean/Phrase |
| S14 | (MH "Long Term Care") | Search modes - Boolean/Phrase |
| S13 | (MH "Cardiovascular Care") | Search modes - Boolean/Phrase |
| S12 | (MH "Ambulatory Care") OR (MH "Ambulatory Care Information Systems") OR (MH "Multidisciplinary Care Team+") | Search modes - Boolean/Phrase |
| S11 | S1 OR S2 OR S3 OR S4 OR S5 OR S6 OR S7 OR S8 OR S9 OR S10 | Search modes - Boolean/Phrase |
| S10 | (MH "Chronic Disease") | Search modes - Boolean/Phrase |
| S9 | (MH "Osteoporosis+") | Search modes - Boolean/Phrase |
| S8 | (MH "Hyperlipidemia+") | Search modes - Boolean/Phrase |
| S7 | (MH "Hypertension+") | Search modes - Boolean/Phrase |
| S6 | (MH "Diabetes Mellitus, Type 2") OR (MH "Diabetes Mellitus+") | Search modes - Boolean/Phrase |
| S5 | MH Osteoarthritis+ | Search modes - Boolean/Phrase |
| S4 | MH Arthritis, Rheumatoid+ | Search modes - Boolean/Phrase |
| S3 | MH Lung Diseases, Obstructive+ | Search modes - Boolean/Phrase |
| S2 | MH Asthma+ | Search modes - Boolean/Phrase |
| S1 | MH Heart Diseases+ | Search modes - Boolean/Phrase |
