# Supplementary material for: A systematic review of chronic disease management interventions in primary care
Source: BMC Fam Pract. 2018 Jan 9;19:11. doi: 10.1186/s12875-017-0692-3 (PMC5759778; doi:10.1186/s12875-017-0692-3)
Supplement: Supplementary file 2 — Full text verification template. (DOCX 27 kb) [file 12875_2017_692_MOESM2_ESM.docx]

**Appendix 2. Full text verification template**

| Endnote Record Number | | |  |  |  |  |
| --- | --- | --- | --- | --- | --- | --- |
| Author and year | |  | | | | |
| Journal |  | | | | | |
| Title |  | | | | | |
| Name/code of reviewer | | |  | | | |

**INSTRUCTIONS: Please tick the appropriate box(es)**

1. **General**

| **Published in English** | | | Yes  No  → ***Do not continue*** | | | |
| --- | --- | --- | --- | --- | --- | --- |
| **Published 2006 - 2014** | | | Yes  No  → ***Do not continue*** | | | |
| **Countries** | | Australia | |  |  | ***You should answer ‘Yes’ to at least one of these countries. Otherwise, please do not continue*** |
|  | | Canada | |  |  |  |
|  | | US | |  |  |  |
|  | | New Zealand | |  |  |  |
|  | | Netherlands | |  |  |  |
|  | | United Kingdom* | |  |  |  |
|  | | Scandinavia** | |  |  |  |
|  | ** England, Scotland, Wales, Northern Ireland* | | | | | |
|  | *** Sweden, Norway, Denmark, Finland, Iceland* | | | | | |

1. **Types of studies**

**Study design**

| RCT | |  | | | | | |
| --- | --- | --- | --- | --- | --- | --- | --- |
| CCT | |  | | | | | |
| CBA | | → If ‘Yes’, | | | | | |
|  | Contemporaneous data collection? | | | | Done  Not clear  Not done | | |
|  | Appropriate choice of control site? | | | | Done  Not clear  Not done | |  |
| ITS | | | → If ‘Yes’, | | | | |
|  | Clearly defined point in time when the intervention occurred? | | | | Done  Not clear  Not done |  |  |
|  | At least 3 data points before and 3 after the intervention? | | | | Done  Not clear  Not done |  |  |
| Other | | (please specify) | |  | | |  |

***If you score ‘Not done’ for any of above criteria, the study should not be included***

**Methodological inclusion criteria**

Objective measurement of outcomes?

*Refers to objective measurement of performance/behaviour of providers/patient outcome(s) in a clinical - not test - situation. Outcome measures such as provider satisfaction with work or patient satisfaction with care may be included if they are assessed using a tool with known reliability and validity.*

Done  Not clear  Not done  → ***Do not continue***

Relevant and interpretable data presented or obtainable?

Done  Not clear  Not done  → ***Do not continue***

1. **Setting**

|  | Hospital | → ***Do not continue*** |
| --- | --- | --- |
|  | Primary care, including family practice |  |
|  | Managed care organization |  |
|  | Community-based, including pharmacy |  |

1. **Types of participants**

**Patients**

|  | **Sex** | Male only |  | | Female only | |  | Both |  |
| --- | --- | --- | --- | --- | --- | --- | --- | --- | --- |
|  | **Age** | <18 years |  | | → ***Do not continue*** | | ≥18 years | |  |
|  | **Condition** | Asthma |  | | | Lipid disorders | | |  |
|  |  | Heart disease*** |  | | | COPD | | |  |
|  |  | Hypertension |  | | | Arthritis (OA or RA) | | |  |
|  |  | Diabetes |  | | | Osteoporosis | | |  |
|  |  | Others (specify) | | → ***Do not continue*** | | | | | |

***including heart failure and myocardial infarction

**Health care professionals**

|  | Doctors |  | Allied health professionals |  |
| --- | --- | --- | --- | --- |
|  | Nurses |  | Lay health workers |  |
|  | Pharmacists |  |  |  |

1. **Types of intervention**

*The intervention to the patient must be delivered by non-hospital health professionals (including doctors, nurses, pharmacists, allied health professionals or lay health workers) in an included setting (see above).*

|  | Professional intervention | Yes  No  → ***Do not continue*** |
| --- | --- | --- |
|  | Patient intervention | Yes  No  → ***Do not continue*** |
|  | Financial intervention | Yes  No  → ***Do not continue*** |
|  | Organisational intervention | Yes  No  → ***Do not continue*** |

1. **Types of outcome measure**

|  | Health professional performance | Yes  No  → ***Do not continue*** |
| --- | --- | --- |
|  | Patient outcomes | Yes  No  → ***Do not continue*** |
|  | Self-report measures with known validity and reliability | Yes  No  → ***Do not continue*** |
|  | Economic measures | Yes  No  → ***Do not continue*** |

BA = controlled before and after study, CCT = controlled clinical trial, COPD = chronic obstructive pulmonary disease, ITS = interrupted time series, OA = osteoarthritis, RA = rheumatoid arthritis, RCT = randomized controlled trial.
