# Supplementary material for: A systematic review of chronic disease management interventions in primary care
Source: BMC Fam Pract. 2018 Jan 9;19:11. doi: 10.1186/s12875-017-0692-3 (PMC5759778; doi:10.1186/s12875-017-0692-3)
Supplement: Supplementary file 3 — Quality assessment template. (DOCX 29 kb) [file 12875_2017_692_MOESM3_ESM.docx]

**Appendix 3. Quality assessment template**

***RCT & CCT***

| Endnote Record Number | | |  |  |  |  | |
| --- | --- | --- | --- | --- | --- | --- | --- |
| Author and year | |  | | | | | |
| Journal |  | | | | | | |
| Title |  | | | | | | |
| Name/code of reviewer | | |  | | | | |
| TOTAL SCORE | | | | | | |  |

**Scoring: DONE=2; NOT CLEAR=1; NOT DONE=0**

1. ***Concealment of allocation (protection against selection bias)***

**DONE _2_ NOT CLEAR _1_ NOT DONE _0_**

*DONE = Randomization process is described explicitly, e.g. random number, coin flips, centralised randomization scheme, an on-site computer system or sealed opaque envelopes.*

*NOT CLEAR = The unit of allocation is not described explicitly OR the unit of allocation was by patient or episode of care and the authors report using a ‘list’ or ‘table’, ‘envelopes’ or ‘sealed envelopes’ for allocation.*

*NOT DONE = Allocation using date of birth, date of admission, hospital numbers or alternation*.

1. ***Adequate follow-up (protection against exclusion bias)***

**DONE _2_ NOT CLEAR _1_ NOT DONE _0_**

*DONE = Outcome measures obtained for 80-100% of subjects randomized.*

*NOT CLEAR = Not specified in the paper.*

*NOT DONE = Outcome measures obtained for less than 80% of subjects randomized.*

1. ***Blinded assessment of primary outcome(s) (protection against detection bias)***

**DONE _2_ NOT CLEAR _1_ NOT DONE _0_**

*DONE = Primary outcome(s) were assessed blindly OR the outcome variables are objective, e.g. length of hospital stay.*

*NOT CLEAR = Not specified in the paper.*

*NOT DONE = The outcome(s) were not assessed blindly.*

***Primary outcome(s) are those variables that correspond to the primary hypothesis or question as defined by the authors. In the event that some of the primary outcome variables were assessed in a blind fashion and others were not, score each separately and label each outcome variable clearly.***

1. ***Baseline measurement***

**DONE _2_ NOT CLEAR _1_ NOT DONE _0_**

*DONE = Outcomes were measured prior to the intervention, and no substantial differences were present across study groups.*

*NOT CLEAR = Not reported, or if it is unclear whether baseline measures are substantially different across study groups.*

*NOT DONE = There are differences at baseline in main outcome measures likely to undermine the post intervention differences (e.g. are differences between the groups before the intervention similar to those found post intervention?).*

1. ***Reliable primary outcome measure(s)***

**DONE _2_ NOT CLEAR _1_ NOT DONE _0_**

*DONE = Two or more raters with at least 90% agreement or kappa ≥0.8 OR the outcome is obtained from some automated system e.g. length of hospital stay, drug levels as assessed by a standardised test.*

*NOT CLEAR = reliability is not reported for outcome measures that are obtained by chart extraction or collected by an individual.*

*NOT DONE = Agreement is less than 90% or kappa is less than 0.8.*

***In the event that some outcome variables were assessed in a reliable fashion and others were not, score each separately on the back of the form and label each outcome variable clearly.***

1. ***Protection against contamination***

**DONE _2_ NOT CLEAR _1_ NOT DONE _0_**

*DONE = Allocation was by community, institution or practice and it is unlikely that the control received the intervention.*

*NOT CLEAR = Professionals were allocated within a clinic or practice and it is possible that communication between experimental and group professionals could have occurred.*

*NOT DONE = It is likely that the control group received the intervention (e.g. cross-over trials or if patients rather than professionals were randomized).*

1. ***Methods of statistical analysis***

**DONE _2_ NOT CLEAR _1_ NOT DONE _0_**

*The study should include a statement describing or giving references for all statistical procedures used.*

***CBA***

| Endnote Record Number | | |  |  |  |  | |
| --- | --- | --- | --- | --- | --- | --- | --- |
| Author and year | |  | | | | | |
| Journal |  | | | | | | |
| Title |  | | | | | | |
| Name/code of reviewer | | |  | | | | |
| TOTAL SCORE | | | | | | |  |

**Scoring: DONE=2; NOT CLEAR=1; NOT DONE=0**

1. ***Baseline measurement***

**DONE _2_ NOT CLEAR _1_ NOT DONE _0_**

*DONE =* *Outcomes were measured prior to the intervention, and no substantial differences were present across study groups.*

*NOT CLEAR =* N*ot reported, or if it is unclear whether baseline measures are substantially different across study groups.*

*NOT DONE = There are differences at baseline in main outcome measures likely to undermine the post intervention differences (e.g. differences between the groups before the intervention similar to those found post intervention).*

1. ***Characteristics for studies using second site as control***

**DONE _2_ NOT CLEAR _1_ NOT DONE _0_**

*DONE =* C*haracteristics of study and control providers are reported and similar.*

*NOT CLEAR =* *Not clear in the paper e.g. characteristics are mentioned in the text but no data are presented.*

*NOT DONE = There is no report of characteristics either in the text or a table OR if baseline characteristics are reported and there are differences between study and control providers.*

1. ***Blinded assessment of primary outcome(s) (protection against detection bias)***

**DONE _2_ NOT CLEAR _1_ NOT DONE _0_**

*DONE =* P*rimary outcome(s) were assessed blindly OR the outcome variables are objective, e.g. length of hospital stay.*

*NOT CLEAR =* *Not specified in the paper.*

*NOT DONE = The outcome(s) were not assessed blindly.*

***Primary outcome(s) are those variables that correspond to the primary hypothesis or question as defined by the authors. In the event that some of the primary outcome variables were assessed in a blind fashion and others were not, score each separately and label each outcome variable clearly.***

1. ***Protection against contamination (studies using second site as control)***

**DONE _2_ NOT CLEAR _1_ NOT DONE _0_**

*DONE = Allocation was by community, institution or practice and it is unlikely that the control received the intervention.*

*NOT CLEAR = Professionals were allocated within a clinic or practice and it is possible that communication between experimental and group professionals could have occurred.*

*NOT DONE = It is likely that the control group received the intervention (e.g. cross-over trials or if patients rather than professionals were randomized).*

1. ***Reliable primary outcome measure(s)***

**DONE _2_ NOT CLEAR _1_ NOT DONE _0_**

*DONE = Two or more raters with at least 90% agreement or kappa ≥0.8 OR the outcome is obtained from some automated system e.g. length of hospital stay, drug levels as assessed by a standardised test.*

*NOT CLEAR = reliability is not reported for outcome measures that are obtained by chart extraction or collected by an individual.*

*NOT DONE = Agreement is less than 90% or kappa is less than 0.8.*

***In the event that some outcome variables were assessed in a reliable fashion and others were not, score each separately on the back of the form and label each outcome variable clearly.***

1. ***Adequate follow-up (protection against exclusion bias)***

**DONE _2_ NOT CLEAR _1_ NOT DONE _0_**

*DONE =* O*utcome measures obtained for 80-100% of subjects randomized.*

*NOT CLEAR =* *Not specified in the paper.*

*NOT DONE = Outcome measures obtained for less than 80% of subjects randomized*.

1. ***Methods of statistical analysis***

**DONE _2_ NOT CLEAR _1_ NOT DONE _0_**

*The study should include a statement describing or giving references for all statistical procedures used.*

***ITS***

| Endnote Record Number | | |  |  |  |  | |
| --- | --- | --- | --- | --- | --- | --- | --- |
| Author and year | |  | | | | | |
| Journal |  | | | | | | |
| Title |  | | | | | | |
| Name/code of reviewer | | |  | | | | |
| TOTAL SCORE | | | | | | |  |

**Scoring: DONE=2; NOT CLEAR=1; NOT DONE=0**

1. ***Protection against secular changes (The intervention is independent of other changes)***

**DONE _2_ NOT CLEAR _1_ NOT DONE _0_**

*DONE =* *The intervention occurred independently of other changes over time.*

*NOT CLEAR =* *Not specified (will be treated as NOT DONE if information cannot be obtained from the authors).*

*NOT DONE = Reported that intervention was not independent of other changes in time.*

1. ***Data were analysed appropriately***

**DONE _2_ NOT CLEAR _1_ NOT DONE _0_**

*DONE = If ARIMA models were used OR time series regression models were used to analyse the data and serial correlation was adjusted/tested for.*

*NOT CLEAR = Not specified (will be treated as NOT DONE if information cannot be obtained from the authors).*

*NOT DONE = Neither of the conditions above not met.*

1. ***Reason for the number of points pre and post intervention given***

**DONE _2_ NOT CLEAR _1_ NOT DONE _0_**

*DONE = Rationale for the number of points stated (eg monthly data for 12 months post-intervention was used because the anticipated effect was expected to decay) OR sample size calculation performed.*

*NOT CLEAR = Not specified (will be treated as NOT DONE if information cannot be obtained from the authors).*

*NOT DONE = Neither of the conditions above not met.*

1. ***Shape of the intervention effect was specified***

**DONE _2_ NOT CLEAR _1_ NOT DONE _0_**

*DONE = Rational explanation for the shape of intervention effect was given by the author(s).*

*NOT CLEAR = Not specified (will be treated as NOT DONE if information cannot be obtained from the authors).*

*NOT DONE = Neither of the conditions above not met.*

1. ***Intervention unlikely to affect data collection (protection against detection bias)***

**DONE _2_ NOT CLEAR _1_ NOT DONE _0_**

*DONE = Reported that intervention itself was unlikely to affect data collection (e.g sources and methods of data collection were the same before and after the intervention).*

*NOT CLEAR = Not reported (will be treated as NOT DONE if information cannot be obtained from the authors).*

*NOT DONE = If the intervention itself was likely to affect data collection (e.g. any change in source or method of data collection reported).*

1. ***Blinded assessment of primary outcome(s) (protection against detection bias)***

**DONE _2_ NOT CLEAR _1_ NOT DONE _0_**

*DONE = Primary outcome(s) were assessed blindly OR the outcome variables are objective, e.g. length of hospital stay.*

*NOT CLEAR = Not specified in the paper (will be treated as NOT DONE if information cannot be obtained from the authors).*

*NOT DONE = The outcome(s) were not assessed blindly.*

***Primary outcome(s) are those variables that correspond to the primary hypothesis or question as defined by the authors. In the event that some of the primary outcome variables were assessed in a blind fashion and others were not, score each separately and label each outcome variable clearly.***

1. ***Completeness of data set***

**DONE _2_ NOT CLEAR _1_ NOT DONE _0_**

*DONE = data set covers 80-100% of total number of participants or episodes of care.*

*NOT CLEAR = Not specified in the paper (will be treated as NOT DONE if information cannot be obtained from the authors).*

*NOT DONE = Data set covers less than 80% of total number of participants or episodes of care.*

1. ***Reliable primary outcome measure(s)***

**DONE _2_ NOT CLEAR _1_ NOT DONE _0_**

*DONE = Two or more raters with at least 90% agreement or kappa ≥0.8 OR the outcome is obtained from some automated system e.g. length of hospital stay, drug levels as assessed by a standardised test.*

*NOT CLEAR = reliability is not reported for outcome measures that are obtained by chart extraction or collected by an individual.*

*NOT DONE = Agreement is less than 90% or kappa is less than 0.8.*

***In the event that some outcome variables were assessed in a reliable fashion and others were not, score each separately.***

CBA = controlled before and after study, CCT = controlled clinical trial, ITS = interrupted time series, RCT = randomized controlled trial.
