# Supplementary material for: A systematic review of chronic disease management interventions in primary care
Source: BMC Fam Pract. 2018 Jan 9;19:11. doi: 10.1186/s12875-017-0692-3 (PMC5759778; doi:10.1186/s12875-017-0692-3)
Supplement: Supplementary file 4 — Summary of the 165 publications included in the review. (DOCX 139 kb) [file 12875_2017_692_MOESM4_ESM.docx]

**Appendix 4: Summary of the 165 publications included in the review**

Note where information on participant age and sex are not shown these were not reported in the publication

| {Armour, 2007 #12} | **Study design:** RCT **CCM elements:** DS **Chronic condition:** Asthma  **Country:** Australia **Setting:** Community-based care **Duration of intervention in (months):** 6.0  **Number of patients:** 396.0 **Number of health professionals:** **Study Aim:** The impact of the Pharmacy Asthma Care Program (PACP) on asthma control was assessed using a multi-site randomised intervention versus control repeated measures study design. | **Group 1 Intervention:**  Usual care  **Group 2 Intervention:** Pharmacist instituted PACP in collaboration with GPs. Ongoing cycle of assessment, management and review in collaboration with GPs. Asthma education manual and training (workshop). Pharmacies were provided with equipment (e.g. spirometers). Pharmacists were offered $200 for their participation on a per patient completed basis | **Mean age group 1:** 50 (SD 16.10)  **Mean age group 2:** 47.5 (SD 17.10)  **Mean age group 3:** (SD )  **Mean age group 4:** (SD )  **% male group 1:** 39.5  **% male group 2:** 32.5  **Ethnicity group 1:** Not reported | **Primary outcome:**  The intervention resulted in improved asthma control: patients receiving the intervention were 2.7 times more likely to improve from "severe" to "not severe" than control patients (OR 2.68, 95% CI: 1.64 to 4.37) SIG  **p =** 0.001 |
| --- | --- | --- | --- | --- |
| {Bebb, 2007 #197} | **Study design:** RCT **CCM elements:** DS **Chronic condition:** Hypertension  **Country:** UK **Setting:** Primary care **Duration of intervention in (months):** 24.0  **Number of patients:** 1534.0 **Number of health professionals:** **Study Aim:** To evaluate the effectiveness of a BP treatment algorithm in primary care patients with type 2 diabetes. | **Group 1 Intervention:**  Usual care  **Group 2 Intervention:** A treatment algorithm designed so that practice nurses and GPs would increase antihypertensive therapy until goal BP reached | **Mean age group 1:** 64 (SD 10)  **Mean age group 2:** 64.3 (SD 9.9)  **% male group 1:** 60.1  **% male group 2:** 58.2    **Ethnicity group 1:** Non-Caucasian | **Primary outcome:**  BP NS  **p =** |
| {Bertoni, 2009 #19} | **Study design:** RCT **CCM elements:** DS **Chronic condition:** Lipid disorders  **Country:** USA **Setting:** Primary care **Duration of intervention in (months):** 35.0  **Number of patients: Number of health professionals:** **Study Aim:** This study reports the effect of a multifactorial intervention on screening of lipid levels and appropriate management of lipid level test results. | **Group 1 Intervention:**  Usual care  **Group 2 Intervention:** Use of hypertension guidelines. One hour intervention specific academic detailing (AD) visit every 6 months. The second AD visit provided practice specific feedback regarding their screen rate for lipid level and appropriate management. | **Mean age group 1:** 45 (SD )  **Mean age group 2:** 47.6 (SD )  **% male group 1:** 44.0  **% male group 2:** 41.0  **Ethnicity group 1:** Non-Hispanic Caucasian | **Primary outcome:**  Appropriate management of lipid levels decreased slightly in ATP-III practices and more markedly in control practices. The net change in appropriate management favoured the intervention (+9.7%, 95% CI: 2.8% to 16.6%) SIG  **p =** 0.010 |
| {Bonds, 2009 #27} | **Study design:** RCT **CCM elements:** DS+SMS **Chronic condition:** Hypertension  **Country:** USA **Setting:** Primary care **Duration of intervention in (months):** 48.0  **Number of patients: Number of health professionals:** **Study Aim:** We conducted a randomized trial in primary care practices of a multifactorial intervention targeted to improve providers' adherence to hypertension guidelines. | **Group 1 Intervention:**  Attention control with similar magnitude but targeted a different guideline  **Group 2 Intervention:** Educational session providing overview of both interventions and control guidelines. Academic detailing sessions were held every 6 months (note: educational material was distributed to professionals and patients during these visits). Automated blood pressure machines provided. Feedback of the pre-intervention hypertension diagnosis and control levels to the practice (note: only for patients had received screening lipid panel in the pre-intervention period) | **Mean age group 1:** 49 (SD 0.9)  **Mean age group 2:** 49.9 (SD 1.4)  **% male group 1:** 41.2  **% male group 2:** 40.4  **Ethnicity group 1:** Non-Hispanic Caucasian | **Primary outcome:**  Percentage of patients at goal NS  **p =** |
| {Davies, 2008 #37} | **Study design:** RCT **CCM elements:** SMS **Chronic condition:** Diabetes  **Country:** UK **Setting:** Primary care **Duration of intervention in (months):** 12.0  **Number of patients:** 824.0 **Number of health professionals:** **Study Aim:** To evaluate the effectiveness of a structured group education programme on biomedical, psychosocial, and lifestyle measures in people with newly diagnosed type 2 diabetes. | **Group 1 Intervention:**  Usual care  **Group 2 Intervention:** A structured group education programme for six hours delivered in the community by two trained healthcare professional educators. Diabetes and self-management for ongoing and newly diagnosed (DESMOND). | **Mean age group 1:** 60 (Range 29-87)  **Mean age group 2:** 59.0 (Range 28-87 )  **% male group 1:** 57.0  **% male group 2:** 53.0  **Ethnicity group 1:** Not reported | **Primary outcome:**  HbA1c NS  **p =** |
| {Estrada, 2011 #55} | **Study design:** RCT **CCM elements:** DS+CIS **Chronic condition:** Diabetes  **Country:** USA **Setting:** Primary care **Duration of intervention in (months):** 24.0  **Number of patients: Number of health professionals:** 205.0 **Study Aim:** To determine the effectiveness of a provider-based education and implementation intervention for improving diabetes control. | **Group 1 Intervention:**  Usual care  **Group 2 Intervention:** Website focused on helping physicians to achieve A1c,BP and LDL control in their DM patients. The website included case-based learning, personalized audit and feedback, and tools designed to facilitate the provision of high-quality care. Intervention arm physicians received email reminders every 1-3 weeks about website updates. | **Mean age group 1:** 61 (SD 13.8)  **Mean age group 2:** 58.7 (SD 13.6)  **% male group 1:** 51.0  **% male group 2:** 49.0  **Ethnicity group 1:** African American | **Primary outcome:**  HbA1c NS  **p =** |
| {Graves, 2009 #68} | **Study design:** Economic evaluation of RCT **CCM elements:** SMS DSD **Chronic condition:**  **Country:** Australia **Setting: Primary Care Duration of intervention in (months):** 12  **Number of patients:** 434 **Number of health professionals:** N/A **Study Aim:** The cost-effectiveness of a Telephone Counselling intervention to improve physical activity and diet, targeting adults with established chronic diseases in a low socio-economic area of a major Australian city was examined. | **Group 1 Intervention:**  Usual care  **Group 2 Intervention:** Telephone counselling intervention for physical activity and diet by trained counsellors. Participants received an intervention workbook along with a pedometer, a self-monitoring form, and an exercise band. | **Mean age group 1:** 58.2 (SD 11.8)  **Mean age group 2:** N/A (SD )  **% male group 1:** 39%  **% male group 2:** N/A  **Ethnicity group 1:** Not reported | **Primary outcome:**  Costs and health benefits compared NS  **p =** |
| {Heisler, 2010 #76} | **Study design:** RCT **CCM elements:** DSD+SMS **Chronic condition:** Diabetes  **Country:** USA **Setting:** Managed care organisation **Duration of intervention in (months):** 6.0  **Number of patients:** 244.0 **Number of health professionals:** **Study Aim:** To compare a reciprocal peer-support (RPS) program with nurse care management (NCM). | **Group 1 Intervention:**  Initial group session to set DM-related goals, receive peer communication skills training and be paired with age-matched peer patient. Peer partners were encouraged to call each other at least once a week. At baseline and 6-months, all participants had their HbA1c and BP checked and were informed of the results. Care managers completed a 4-hour training course in motivational interviewing  **Group 2 Intervention:** Patients in NCM group attended a 1.5 hr session to review their labortatory blood results. At baseline and 6-months, all participants had their HbA1c and BP checked and were informed of the results. Self-management educational material, motivation counselling | **Mean age group 1:** 62 (SD 6.1)  **Mean age group 2:** 62.3 (SD 6.3)  **% male group 1:** 100.0  **% male group 2:** 100.0  **Ethnicity group 1:** Non-Hipanic Caucasian | **Primary outcome:**  HbA1c SIG  **p =** 0.004 |
| {Khan, 2010 #86} | **Study design:** RCT **CCM elements:** CIS+DS **Chronic condition:** Diabetes  **Country:** USA **Setting:** Primary care **Duration of intervention in (months):** 32.0  **Number of patients:** 7412.0 **Number of health professionals:** **Study Aim:** To describe the effect of the Vermont Diabetes Information System (VDIS) on hospital and emergency room use | **Group 1 Intervention:**  Usual care  **Group 2 Intervention:** VIDS receives lab results from clinical labs maintains a registery and produces reports to primary care physicians and patients. Guidelines and recommendations to physicians. Alert letters for the patients when the results are above target. Population-level reports are sent to physicians with a report card indicating population-level performance | **Mean age group 1:** 62 (SD )  **Mean age group 2:** 63.5 (SD )  **Mean age group 3:** (SD )  **Mean age group 4:** (SD )  **% male group 1:** 48.1  **% male group 2:** 49.8  **Ethnicity group 1:** Caucasian | **Primary outcome:**  Hospital charges (Lower for intervention group) SIG  **p =** 0.019 |
| {Lorig, 2007 #96} | **Study design:** RCT **CCM elements:** SMS **Chronic condition:** Diabetes  **Country:** USA **Setting:** Community based care **Duration of intervention in (months):** 18.0  **Number of patients:** 567.0 **Number of health professionals:** **Study Aim:** To determine 1) whether participants in the Spanish Diabetes Self Management Program (SDSMP), when compared at 6 months to randomized control subjects, would demonstrate improvements in health status, health behaviors, and self-efficacy; and 2) whether SDSMP participants receiving monthly automated telephone reinforcement would maintain improvements at 18 months better than those not receiving reinforcement. | **Group 1 Intervention:**  Spanish Diabetes self-management program only  **Group 2 Intervention:** Spanish diabetes self management program plus monthly automated telephone messages | **Mean age group 1:** 53 (SD 13.4)  **Mean age group 2:** 52.9 (SD 13.2)  **% male group 1:** 32.8  **% male group 2:** 42.9  **Ethnicity group 1:** Hispanic/Latino | **Primary outcome:**  HbA1c SIG  **p =** 0.040 |
| {MacLean, 2009 #98} | **Study design:** **CCM elements:** **Chronic condition:** Diabetes  **Country:** USA **Setting: Primary Care Duration of intervention in (months):** 12  **Number of patients: Number of health professionals:** **Study Aim:** To evaluate the impact of a registry and decision support system on processes of care, and physiologic control. | **Group 1 Intervention:**  Usual care  **Group 2 Intervention:** Reminders of overdue diabetes tests Alert of abnormal results Quarterly population reports and peer comparisons | **Mean age group 1:** not reported **Mean age group 2: not reported** (SD )  **% male group 1:** not reported  **% male group 2:** Not reported  **Ethnicity group 1:** Not reported | **Primary outcome:**  HbA1c NS  **p =** |
| {Stone, 2010 #292}{Stone, 2010 #292} | **Study design:** RCT **CCM elements:** CIS+SMS+DSD **Chronic condition:** Diabetes  **Country:** USA **Setting:** Community-based care **Duration of intervention in (months):** 6.0  **Number of patients:** 150.0 **Number of health professionals:** **Study Aim:** To compare the short-term efficacy of home telemonitoring coupled with active medication management by a nurse practitioner with a monthly care coordination telephone call on glycemic control in veterans with type 2 diabetes and entry A1C > or =7.5%. | **Group 1 Intervention:**  2-h educational session for diabetes management and nutrition. Monthly telephone calls from the study diabetes nurse educator regarding general health conditions, status of glycemic control, BP and weight. Issues requiring active intervention were referred to their PCP. Participants also could initiate contact with the study diabetes nurse educator to discuss concerns related to diabetes management  **Group 2 Intervention:** Participants in ACM-HT group received a 6-month diabetes management support intervention using the the Viterion 100 Monitor home telemonitoring device. 2-h educational session for diabetes management and nutrition. The nurse practitioner reviewed SMBG, blood pressure, weight and risk stratifications generated by the Viterion and contacted participants as necessary. The nurse practitioner provided timely telephone follow-up, including further self management education for participants who generated "high risk" reports | **Mean age group 1:** 33% >=65  **Mean age group 2:** 33% >=65  **% male group 1:** 97%  **% male group 2:** 100%  **Ethnicity group 1:** Not reported | **Primary outcome:**  HbA1c SIG  **p =** 0.001 |
| {Tobe, 2006 #163} | **Study design:** RCT **CCM elements:** DS+DSD **Chronic condition:** Hypertension  **Country:** Canada **Setting:** Community-based care **Duration of intervention in (months):** 12.0  **Number of patients:** 99.0 **Number of health professionals:** **Study Aim:** A randomized controlled trial to assess whether a community-based treatment strategy implemented by home care nurses would be effective in controlling hypertension in First Nations people with existing hypertension and type 2 diabetes. | **Group 1 Intervention:**  Control group, treatment decisions were made by subject's primary care physician  **Group 2 Intervention:** In the intervention group, a home care nurse followed a predefined treatment algorithm of pharmacologic antihypertensive therapy. All participants were seen by a home care nurse in the medical clinic on their reserve baseline, at 6 and at 3,6,9 and 12 months after enrolement. All patients received healthy lifestyle classes. A letter summarizing the patient's current medications, blood pressure and updated laboratory results was completed and forwarded to the primary care physician. | **Mean age group 1:** 56 (SD 11.5)  **Mean age group 2:** 55.4 (SD 12.9)  **% male group 1:** 39.0  **% male group 2:** 38.0  **Ethnicity group 1:** Indigenous | **Primary outcome:**  SBP NS  **p =** |
| {Zwar, 2012 #193} | **Study design:** RCT **CCM elements:** DSD+DS+SMS **Chronic condition:** COPD  **Country:** Australia **Setting:** Community-based care **Duration of intervention in (months):** 12.0  **Number of patients:** 44.0 **Number of health professionals:** **Study Aim:** To evaluate a partnership model of care for patients with a diagnosis of chronic obstructive pulmonary disease (COPD). | **Group 1 Intervention:**  Usual care  **Group 2 Intervention:** Two registered nurses worked in patnership with GPs to implement the intervention. Care plan developed to provide management of COPD according to Australian and New Zealand guidelines. The nurse training covered ther principles and practice of motivational interviewing and self-management. | **Mean age group 1:** 64 (SD 10.3)  **Mean age group 2:** 65.8 (SD 10.3)  **% male group 1:** 48.8  **% male group 2:** 47.0  **Ethnicity group 1:** Not reported | **Primary outcome:**  The primary outcome was disease-related quality of life measured using the St George’s Respiratory Questionnaire (SGRQ) at 12-month follow-up. NS  **p =** |
| {Wang, 2012 #180} | **Study design:** RCT **CCM elements:** CIS+SMS **Chronic condition:** Hypertension  **Country:** USA **Setting:** Community-based care **Duration of intervention in (months):** 18.0  **Number of patients:** 591.0 **Number of health professionals:** **Study Aim:** This study evaluated the costs of the Hypertension Intervention Nurse Telemedicine Study (HINTS), aimed to improve BP control in veterans. | **Group 1 Intervention:**  Usual care  **Group 2 Intervention:** Home BP monitor and telemedicine. If BP adequately controlled then 6-monthly nurse phone calls to reinforce behaviour. Behavioural intervention - If BP control inadequate with home monitoring then PN phone patient to deliver tailored, patient-specific and pre-scripted modules to improve management and behaviours.  **Group 3 Intervention:** Home BP monitor and telemedicine. If BP adequately controlled then 6-monthly nurse phone calls to reinforce behaviour. If control inadequate the PN notified the GP and presented medication recommendation based on standardised EB protocol. The PN communicated GP recommendations to patient  **Group 4 Intervention:** Home BP monitor and telemedicine. If BP adequately controlled then 6-monthly nurse phone calls to reinforce behaviour. If inadequate control then patient received both medication management and behavioural management intervention | **Mean age group 1:** 64 (SD 10)  **Mean age group 2:** 63.0 (SD 11)  **Mean age group 3:** 64.0 (SD 10.0)  **Mean age group 4:** 63.0 (SD 10.0)  **% male group 1:** 96.0  **% male group 2:** 92.0  **% male group 3:** 93.0  **% male group 4:** 86.0  **Ethnicity group 1:** Caucasian | **Primary outcome:**  BP NS  **p =** NS |
| {Shah, 2014 #139} | **Study design:** RCT **CCM elements:** DS **Chronic condition:** Diabetes  **Country:** Canada **Setting:** Primary care **Duration of intervention in (months):** 10.0  **Number of patients:** 933789.0 **Number of health professionals:** **Study Aim:** The objective of this pragmatic cluster randomized trial was to evaluate the effectiveness of an educational toolkit focusing on cardiovascular disease screening and risk reduction in people with diabetes | **Group 1 Intervention:**  Usual care  **Group 2 Intervention:** Educational toolkit for cardiovascular disease screening and risk reduction for health professionals | **Mean age group 1:** 64 (SD 12.4)  **Mean age group 2:** 64.3 (SD 12.4)  **% male group 1:** 52.6  **% male group 2:** 52.8  **Ethnicity group 1:** Not reported | **Primary outcome:**  Mortality NS  **p =** |
| {Siminerio, 2013 #142} | **Study design:** RCT **CCM elements:** DSD+SMS **Chronic condition:** Diabetes  **Country:** USA **Setting:** Primary care **Duration of intervention in (months):** 6.0  **Number of patients:** 141.0 **Number of health professionals:** **Study Aim:** The purpose of this comparative effectiveness study is to compare diabetes self-management support (DSMS) approaches and determine who can be most effective in helping patients maintain/improve clinical outcomes, self-care behaviors, distress, and satisfaction following diabetes self-management education (DSME) delivered in primary care. | **Group 1 Intervention:**  Usual care  **Group 2 Intervention:** Educator self management support **Group 3 Intervention:** Peer self-management support **Group 4 Intervention:** Primary care self management support | **Mean age group 1:** 60 (SD 12)  **Mean age group 2:** 60.0 (SD 10)  **Mean age group 3:** 64.0 (SD 10.0)  **Mean age group 4:** 60.0 (SD 13.4)  **% male group 1:** 46.9  **% male group 2:** 42.1  **% male group 3:** 47.2  **% male group 4:** 40.0  **Ethnicity group 1:** Caucasian | **Primary outcome:**  HbA1c NS  **p =** |
| {Smidth, 2013 #146} | **Study design:** RCT **CCM elements:** SMS+DS+DSD **Chronic condition:** COPD  **Country:** Denmark **Setting:** Primary care **Duration of intervention in (months):** 12.0  **Number of patients:** 744.0 **Number of health professionals:** **Study Aim:** The aim of this paper is to present the effect of the previously developed active implementation model for a disease management plan for COPD on patients’ self assessment of their care | **Group 1 Intervention:**  Usual care  **Group 2 Intervention:** Disease management plan for COPD based on GOLD guidelines | **Mean age group 1:** 67 (SD )  **Mean age group 2:** 68.3 (SD )  **% male group 1:** 48.1  **% male group 2:** 48.3  **Ethnicity group 1:** Not reported | **Primary outcome:**  PACIC (Patient Assessment of Chronic Illness Care) NS  **p =** |
| {Smidth, 2013 #145} | **Study design:** **CCM elements:** SMS DS DSD **Chronic condition:**  **Country:** Denmark **Setting: Primary Care Duration of intervention in (months):** 12  **Number of patients: Number of health professionals:** **Study Aim:** The objective of this paper was to determine the effect on healthcare-utilization of an active implementation model for a disease-management-program for patients with one of the major multimorbidity diseases, COPD. | **Group 1 Intervention:**  Usual care  **Group 2 Intervention:** Disease management plan for COPD based on GOLD guidelines  **Group 3 Intervention:** External control | **Mean age group 1:** 63.9 (SD )  **Mean age group 2:** 63.3 (SD )  **Mean age group 3: 63.3**  **% male group 1:** 45.5  **% male group 2:** 46.3  **% male group 3:** 45.0  **Ethnicity group 1:** Not reported | **Primary outcome:**  Planned preventative consultation SIG  **p =** 0.010 |
| {Steventon, 2014 #152} | **Study design:** RCT **CCM elements:** DSD+SMS+CIS **Chronic condition:** Diabetes  **Country:** UK **Setting:** Primary care **Duration of intervention in (months):** 12.0  **Number of patients:** 3230.0 **Number of health professionals:** **Study Aim:** The Whole Systems Demonstrator was a large, pragmatic, cluster randomised trial that compared telehealth with usual care among 3,230 patients with long-term conditions in three areas of England. Telehealth involved the regular transmission of physiological information such as blood glucose to health professionals working remotely. We examined whether telehealth led to changes in glycosylated haemoglobin (HbA1c) among the subset of patients with type 2 diabetes. | **Group 1 Intervention:**  Usual care  **Group 2 Intervention:** Telehealth equipment to monitor diabetes or COPD. Patient took measurements which were uploaded to centre where clinical staff repsonded to results | **Mean age group 1:** 66 (SD 11.9)  **Mean age group 2:** 63.9 (SD 13)  **% male group 1:** 64.3  **% male group 2:** 53.3  **Ethnicity group 1:** Caucasian | **Primary outcome:**  HbA1c SIG  **p =** 0.013 |
| {Stoddart, 2013 #153} | **Study design:** **CCM elements:** DSD SMS CIS **Chronic condition:**  **Country:** Scotland **Setting: Primary Care Duration of intervention in (months):** 6  **Number of patients:** 401 **Number of health professionals:** **Study Aim:** To compare the costs and cost-effectiveness of managing patients with uncontrolled blood pressure (BP) using telemonitoring versus usual care from the perspective of the National Health Service (NHS) | **Group 1 Intervention:**  Six month telemonitoring sevrice for BP management. BP measurements uploaded and reviewed by clinical staff and text dcision support **Group 2 Intervention:** Usual care | **Mean age group 1:** 60.5 (SD 11.8 )  **Mean age group 2:** 60.8 (SD 10.7)  **% male group 1:** 58.5  **% male group 2:** 59.7  **Ethnicity group 1:** Not reported | **Primary outcome:**  Total healthcare costs SIG  **p =** 0.010 |
| {Stuurman-Bieze, 2014 #157} | **Study design:** ITS **CCM elements:** DSD+CIS+DS **Chronic condition:** Osteoporosis  **Country:** Netherlands **Setting:** Community **Duration of intervention in (months):** 12.0  **Number of patients:** 937.0 **Number of health professionals:** **Study Aim:** The aim of this study was to determine the effects of a community pharmacists’ intervention program on the 1-year discontinuation and nonadherence rates of patients initiating osteoporosis medication. | **Group 1 Intervention:**  Usual care  **Group 2 Intervention:** Medication Monitoring and Optimisation (MeMO) intervention of continous monitoring, tailored counselling with community pharmacist | **Mean age group 1:** 67 (SD 13.9)  **Mean age group 2:** 67.0 (SD 15.2)  **% male group 1:** 21.5  **% male group 2:** 23.0    **Ethnicity group 1:** Not reported | **Primary outcome:**  Medication discontinuation SIG  **p =** 0.010 |
| {Thom, 2014 #161} | **Study design:** RCT **CCM elements:** DSD+SMS **Chronic condition:** Diabetes  **Country:** USA **Setting:** Primary care **Duration of intervention in (months):** 12.0  **Number of patients:** 441.0 **Number of health professionals:** **Study Aim:** To assess the impact of health coaching on patients’ in their primary care provider. | **Group 1 Intervention:**  Usual care  **Group 2 Intervention:** Health coaching face to face and by telephone for management of diabetes | **Mean age group 1:** 53 (SD 11.5)  **Mean age group 2:** 52.6 (SD 10.7)  **% male group 1:** 47.8  **% male group 2:** 41.5  **Ethnicity group 1:** Hispanic/Latino | **Primary outcome:**  TIPS (Trust in Physician Scale) SIG  **p =** 0.047 |
| {Ulrik, 2012 #165} | **Study design:** CCT **CCM elements:** DS **Chronic condition:** COPD  **Country:** Denmark **Setting:** Primary care **Duration of intervention in (months):**  **Number of patients: Number of health professionals:** 1925 **Study Aim:** To determine whether participating in a standardised educational program delivered in the GP’s own practice is associated with adherence to COPD guidelines | **Group 1 Intervention:**  Educational program for GP practices, 3-hr teaching and 5 visits from pharmaceutical representative **Group 2 Intervention:** Usual care | **Mean age group 1:** (SD )  **Mean age group 2:** (SD )  **% male group 1:**  **% male group 2:**  **Ethnicity group 1:** Not reported | **Primary outcome:**  Preventative consultations NS  **p =** |
| {Vagholkar, 2014 #166} | **Study design:** RCT **CCM elements:** DS **Chronic condition:** Hypertension  **Country:** Australia **Setting:** Primary care **Duration of intervention in (months):** 12  **Number of patients:** 1074.0 **Number of health professionals:** 36 **Study Aim:** This randomized controlled trial investigated the impact of CVD absolute risk assessment in family practice on management of cardiovascular risk, including prescription of antihypertensive and lipid-lowering medication. | **Group 1 Intervention:**  Usual care  **Group 2 Intervention:** Health professional education on CVD absolute risk, use of electronic risk calculator | **Mean age group 1:** 57 (SD 6.9)  **Mean age group 2:** 56.2 (SD 6.6)  **% male group 1:** 38.5  **% male group 2:** 45.0  **Ethnicity group 1:** Caucasian | **Primary outcome:**  Antihypertensives NS  **p =** |
| {Van den Donk, 2013 #169} | **Study design:** ITS **CCM elements:** DS **Chronic condition:** COPD  **Country:** Netherlands **Setting:** Community **Duration of intervention in (months):** 1.0  **Number of patients:** 63.0 **Number of health professionals:** **Study Aim:** The study aimed to examine the effects of intensive treatment (IT) vs routine care (RC) on patient-reported outcomes after 5 years in screen-detected diabetic patients | **Group 1 Intervention:**  Usual care  **Group 2 Intervention:** Intensive treatment using treatment targets and algorithms for diabetes, hypertension and cholesterol | **Mean age group 1:** 69 (SD 9.9)  **Mean age group 2:** 68.9 (SD 9.9)  **% male group 1:** 51.0  **% male group 2:** 51.0  **Ethnicity group 1:** Not reported | **Primary outcome:**  Quality of life measured with Euroquol 5 Dimensions NS  **p =** |
| {van der Heijden, 2014 #170} | **Study design:** CCT **CCM elements:** DS+CIS **Chronic condition:** Diabetes  **Country:** Netherlands **Setting:** Managed care **Duration of intervention in (months):** 12.0  **Number of patients:** 783.0 **Number of health professionals:** **Study Aim:** The aim of this study is to evaluate the care process and costs of managed, protocolized and usual care for type 2 diabetes patients from a societal perspective | **Group 1 Intervention:**  Usual care  **Group 2 Intervention:** Managed care **Group 3 Intervention:** Protocolised care | **Mean age group 1:** 51 (SD 7)  **Mean age group 2:** 52.1 (SD 7.4)  **Mean age group 3:** 53.8 (SD 7.5)  **% male group 1:** 51.1  **% male group 2:** 52.1  **% male group 3:** 53.8  **Ethnicity group 1:** Not reported | **Primary outcome:**  Feet examination SIG  **p =** 0.049 |
| {Vitiello, 2013 #175} | **Study design:** RCT **CCM elements:** SMS+DSD **Chronic condition:** Arthritis  **Country:** USA **Setting:** Primary care **Duration of intervention in (months):** 9.0  **Number of patients:** 367.0 **Number of health professionals:** **Study Aim:** To assess whether older persons with osteoarthritis (OA) pain and insomnia receiving cognitive–behavioral therapy for pain and insomnia (CBT-PI), a cognitive–behavioral pain coping skills intervention (CBT-P),and an education-only control (EOC) differed in sleep and pain outcomes | **Group 1 Intervention:**  Education only  **Group 2 Intervention:** CBT for pain and insomnia  **Group 3 Intervention:** CBT pain coping skills | **Mean age group 1:** 73 (SD 8)  **Mean age group 2:** 73.0 (SD 8.4)  **Mean age group 3:** 73.2 (SD 8.1)  **% male group 1:** 24.4  **% male group 2:** 19.7  **% male group 3:** 20.5  **Ethnicity group 1:** Caucasian | **Primary outcome:**  Insomnia severity SIG  **p =** 0.010 |
| {Walters, 2013 #179} | **Study design:** RCT **CCM elements:** DSD+SMS **Chronic condition:** COPD  **Country:** Australia **Setting:** Primary care **Duration of intervention in (months):** 12.0  **Number of patients:** 182.0 **Number of health professionals:** **Study Aim:** To assess benefits of telephone-delivered health mentoring in community-based chronic obstructive pulmonary disease (COPD). | **Group 1 Intervention:**  Usual care plus non-interventional telephone calls  **Group 2 Intervention:** Health mentor telephone calls to manage illness and behaviour change by trained community nurses | **Mean age group 1:** 67 (SD 7.6)  **Mean age group 2:** 68.2 (SD 7.9)  **% male group 1:** 47.0  **% male group 2:** 49.0    **Ethnicity group 1:** Not reported | **Primary outcome:**  SF-36 NS  **p =** |
| {Welschen, 2012 #184} | **Study design:** RCT **CCM elements:** SMS+DSD **Chronic condition:** Diabetes  **Country:** Netherlands **Setting:** Managed care **Duration of intervention in (months):** 12.0  **Number of patients:** 154.0 **Number of health professionals:** **Study Aim:** Effects of a cognitive behavioural treatment (CBT) in type 2 diabetes patients were studied in a randomised controlled trial | **Group 1 Intervention:**  Managed care  **Group 2 Intervention:** Managed care plus CBT | **Mean age group 1:** 61 (SD 8.800)  **Mean age group 2:** 60.5 (SD 9.4)  **% male group 1:** 64.2  **% male group 2:** 59.5  **Ethnicity group 1:** Caucasian | **Primary outcome:**  Coronary heart disease risk NS  **p =** |
| {Wermeling, 2013 #186} | **Study design:** RCT **CCM elements:** CIS **Chronic condition:** Diabetes  **Country:** Netherlands **Setting:** Primary care **Duration of intervention in (months):** 18.0  **Number of patients:** 2215.0 **Number of health professionals:** 233.0 **Study Aim:** The aim of the study was to determine the satisfaction of well-controlled type 2 diabetes patients with either three-monthly or six-monthly diabetes monitoring and their future preference | **Group 1 Intervention:**  3 month monitoring  **Group 2 Intervention:** 6 month monitoring | **Mean age group 1:** 65 (SD 8.800)  **Mean age group 2:** 64.4 (SD 8.800)  **% male group 1:** 60.8  **% male group 2:** 58.5  **Ethnicity group 1:** Caucasian | **Primary outcome:**  Satisfaction with monitoring SIG  **p =** 0.010 |
| {Wermeling, 2014 #185} | **Study design:** **CCM elements:** DSD **Chronic condition:** Diabetes  **Country:** Netherlands **Setting:** Primary Care **Duration of intervention in (months):** 18  **Number of patients: Number of health professionals:** **Study Aim:** To investigate effectiveness and cost-effectiveness of 6-monthly monitoring compared with 3-monthly monitoring of well-controlled type 2 diabetes patients in primary care | **Group 1 Intervention:**  3 month monitoring  **Group 2 Intervention:** 6 month monitoring | **Mean age group 1:** 64.7 (SD 8.8)  **Mean age group 2:** 64.4 (SD 8.8)  **% male group 1:** 60.8  **% male group 2:** 58.5  **Ethnicity group 1:** Not reported | **Primary outcome:**  HbA1c NS  **p =** |
| {Williams, 2013 #188} | **Study design:** RCT **CCM elements:** SMS+DS **Chronic condition:** Diabetes, Osteoarthritis  **Country:** Australia **Setting:** Primary care **Duration of intervention in (months):** 4.0  **Number of patients:** 247.0 **Number of health professionals:** **Study Aim:** This paper presents the evaluation of “Moving On”, a generic self-management program for people with a chronic illness developed by Arthritis NSW. The program aims to help participants identify their need for behaviour change and acquire the knowledge and skills to implement changes that promote their health and quality of life | **Group 1 Intervention:**  Light physical activity control  **Group 2 Intervention:** Arthritis self-management program | **Mean age group 1:** (SD )  **Mean age group 2:** (SD )  **% male group 1:** 34.9  **% male group 2:** 34.9  **Ethnicity group 1:** Not reported | **Primary outcome:**  Self efficacy NS  **p =** |
| {Wilson, 2014 #189} | **Study design:** RCT **CCM elements:** DSD+SMS+DS **Chronic condition:** Diabetes  **Country:** UK **Setting:** Primary care **Duration of intervention in (months):** 18.0  **Number of patients:** 1997.0 **Number of health professionals:** **Study Aim:** The objective of the current study is to evaluate the clinical and cost effectiveness of the ICCDs based in three primary care trusts (PCTs) in England in a cluster randomised controlled trial. | **Group 1 Intervention:**  Usual care  **Group 2 Intervention:** Access to Intermediate Care Clinics for Diabetes with specialist diabetes nurse, special interest GP, guidelines, self-management | **Mean age group 1:** (SD )  **Mean age group 2:** (SD )  **% male group 1:** 58.1  **% male group 2:** 58.4  **Ethnicity group 1:** Caucasian | **Primary outcome:**  Combined score NS  **p =** |
| {Bennett, 2012 #198} | **Study design:** RCT **CCM elements:** SMS+DSD **Chronic condition:** Hypertension  **Country:** USA **Setting:** Primary care **Duration of intervention in (months):** 24.0  **Number of patients:** 365.0 **Number of health professionals:** **Study Aim:** We conducted a pragmatic randomized controlled trial26-28 to evaluate the effectiveness of a behavioral intervention that emphasized weight loss and hypertension medication adherence among primary care patients in the community health center setting. | **Group 1 Intervention:**  Usual care  **Group 2 Intervention:** Behavioral intervention that promoted weight loss and hypertension self-management using eHealth components. The intervention included tailored behavior change goals, self-monitoring, and skill training, available via a website or interactive voice response. 18 telephone counseling calls; primary care provider endorsement; 12 optional group support sessions; and links with community resources **.** | **Mean age group 1:** 55 (SD 11)  **Mean age group 2:** 54.6 (SD 10.8)  **% male group 1:** 34.1  **% male group 2:** 28.9  **Ethnicity group 1:** African American | **Primary outcome:**  Weight Adjusted diff between mean weight change = -1.07 (-1.94 to -0.22)  **p =** not reported |
| {Silverman, 2012 #293} | **Study design:** RCT **CCM elements:** SMS **Chronic condition:** Osteoporosis  **Country:** USA **Setting:** Community-based care **Duration of intervention in (months):** 12.0  **Number of patients:** 240 **Number of health professionals:** **Study Aim:** We examined whether reporting of bone turnover marker results, education about osteoporosis, or a combination of both would increase persistence to oral bisphosphonates. | **Group 1 Intervention:**  Usual care  **Group 2 Intervention:** Bone marker results at baseline, 3 months and 12 months **Group 3 Intervention:** Educational material every month and a membership in the National Osteoporosis Association **Group 4 Intervention:** Bone marker results at baseline, 3 months and 12 months and educational material every month and a membership in the National Osteoporosis Association | **Mean age group 1:** 69 (SD 1.2)  **Mean age group 2:** 67.7 (SD 1.2)  **Mean age group 3:** 66.2 (SD 1.0)  **Mean age group 4:** 66.1 (SD 1.2)  **% female group 1:** 100%  **% female group 2:** 100%  **% female group 3:** 100%  **% female group 4:** 100%  **Ethnicity group 1:** Not reported | **Primary outcome:**  Overall persistence (oral biphosphonate) NS  **p =** |
| {Sperl-Hillen, 2011 #272} | **Study design:** RCT **CCM elements:** SMS **Chronic condition:** Diabetes  **Country:** USA **Setting:** Primary care **Duration of intervention in (months):** 7.0  **Number of patients:** 623.0 **Number of health professionals:** **Study Aim:** We conducted the present study, called the Journey for Control of Diabetes Interactive Dialogue to Educate and Activate (IDEA) study, to determine if group education (GE) using this approach improves glucose control and psychosocial and behavioral outcomes compared with usual care (UC, no assigned education) and with individual education (IE, the conventional approach) for patients with established type 2 diabetes and suboptimal control (HbA1c concentration, ≥7%). | **Group 1 Intervention:**  Usual care  **Group 2 Intervention:** 3 individual 1-hour sessions with the certified diabetes eduacators. As needed to meet national standards for diabetes self-management. Educators in both care systems received expert training on how to facilitate GE sessions though the creator of the Conversation Map program, Healthy Interaction Inc **Group 3 Intervention:** four 2-hour sessions in groups | **Mean age group 1:** 63 (SD 11.5)  **Mean age group 2:** 61.6 (SD 10.9)  **Mean age group 3:** 61.2 (SD 11.8)  **Mean age group 4:** (SD )  **% male group 1:** 53.7  **% male group 2:** 49.0  **% male group 3:** 50.4  **Ethnicity group 1:** Caucasian | **Primary outcome:**  HbA1c SIG  **p =** 0.010 |
| {Frosch, 2011 #61} | **Study design:** RCT **CCM elements:** SMS **Chronic condition:** Diabetes  **Country:** USA **Setting:** Primary care **Duration of intervention in (months):** 6.0  **Number of patients:** 201.0 **Number of health professionals:** **Study Aim:** This study compares the efficacy of an intervention package with controls for diabetes control. | **Group 1 Intervention:**  Participants assigned to the control condition received a 20-page brochure entitled "4 steps to control your diabetes for life". Participants received a call 1 week after enrollment to remind them to review the materials provided  **Group 2 Intervention:** Participants received a 24-minute-long DVD program with accompanying booklet entitled Living with diabetes: Making lifestyle changes to last a lifetime. Up to 5 sessions of telephone coaching with a bilingual nurse educator trained in patient-centered approached to diabetes management and motivational enhancement. Participants received a call 1 week after enrollment to remind them to review the intervention materials provided | **Mean age group 1:** 54 (SD 8.9)  **Mean age group 2:** 56.7 (SD 8.300)  **% male group 1:** 57.0  **% male group 2:** 46.0  **Ethnicity group 1:** Hispanic/Latino | **Primary outcome:**  HbA1c NS  **p =** |
| {Allen, 2011 #9} | **Study design:** RCT **CCM elements:** SMS+DSD **Chronic condition:** Heart disease  **Country:** USA **Setting:** Community-based care **Duration of intervention in (months):** 12.0  **Number of patients:** 525.0 **Number of health professionals:** **Study Aim:** This report describes the results of a randomized, controlled clinical trial evaluating the effectiveness of a comprehensive program of cardiovascular disease risk reduction delivered by nurse practitioner /community health worker (NP/CHW) teams versus enhanced usual care (EUC) to improve lipids, blood pressure, glycated hemoglobin (HbA1c), and patient perceptions of the quality of their chronic illness care in patients in urban community health centers. | **Group 1 Intervention:**  Enhanced usual care. Patients and their providers in the EUC group received the results of baseline lipids, BP, and HbA1c along with the recommended goal levels and a pamphlet on controlling risk factors published by the American Heart Association.  **Group 2 Intervention:** The NP and CHW worked as a team. The NP functioned as a case coordinator for each participant. She tailored the intervention plan, conducted the interventions as lifestyle modification, counceling, medication titration The NP consulted physician, and supervised CHW methods to optimize therapy, and strategies to improve medication adherence. Specified algorithms for drug treatments were developed for this study based on current guidelines and standards of care. The CHW problem solved anticipated barriers to tratment adherence, assisted patients in designing a set of reminders, prompts, etc to assist in following complex regimens | **Mean age group 1:** 55 (SD 11.5)  **Mean age group 2:** 54.3 (SD 12)  **% male group 1:** 29.2  **% male group 2:** 28.3  **Ethnicity group 1:** African American | **Primary outcome:**  Lipids SIG  **p =** 0.001 |
| {Allaire, 2011 #8} | **Study design:** CCT **CCM elements:** DS **Chronic condition:** Hypertension  **Country:** USA **Setting:** Primary care **Duration of intervention in (months):** 24.0  **Number of patients:** 16366.0 **Number of health professionals:** **Study Aim:** A continuing medical education (CME) program trained primary care providers in evidence-based guidelines for hypertension prevention and control. This study evaluated its effectiveness in reducing patients' blood pressure for the sessions occurring from 2003 to 2007 | **Group 1 Intervention:**  Usual care  **Group 2 Intervention:** Continous Medical Education Program (CME) | **Mean age group 1:** 52 (SD )  **Mean age group 2:** 52.5 (SD )  **% male group 1:** 44.5  **% male group 2:** 44.2    **Ethnicity group 1:** African American | **Primary outcome:**  SBP SIG Difference in SBP is -1.99 (SE 0.38) Estimate significant at 95% confidence level  **p =** |
| {ter Bogt, 2011 #159} | **Study design:** RCT **CCM elements:** SMS+DSD+DS **Chronic condition:** Hypertension  **Country:** Netherlands **Setting:** Primary care **Duration of intervention in (months):** 12.0  **Number of patients:** 457.0 **Number of health professionals:** **Study Aim:** The Groningen Overweight and Lifestyle (GOAL) study primarily aims at preventing weight gain by nurse practitioners (NP) guided by a standardized computerized software program. Since favourable changes in physical activity (PA) and diet may improve health independently of weight (loss), insight into effects on lifestyle habits is essential. We examined the 1-year effects of lifestyle counselling by NP on PA and diet, compared with usual care from the general practitioner (GP-UC). | **Group 1 Intervention:**  Participants in the control group were offered one visit with their GP to discuss results from the screening and thereafter received Usual care  **Group 2 Intervention:** Physical activity and diet; guide also given to GP and GP to deliver counselling. GOAL study primarily aims at preventing weight gain by nurse practitioners (NP) guided by a standardized computerized software program In the first year, the intervention of the NP consisted of four individual visits and one feedback session by telephone. | **Mean age group 1:** 57 (SD 7.7)  **Mean age group 2:** 55.2 (SD 7.7)  **% male group 1:** 45.3  **% male group 2:** 48.5  **Ethnicity group 1:** Not reported | **Primary outcome:**  Changes in dietary intake NS  **p =** |
| {Bosworth, 2011 #28} | **Study design:** RCT **CCM elements:** SMS **Chronic condition:** Hypertension  **Country:** USA **Setting:** Managed care organisation **Duration of intervention in (months):** 24.0  **Number of patients:** 636 **Number of health professionals:** **Study Aim:** The goal of this post hoc analysis was to examine the effectiveness of 2 patient-directed interventions designed to improve blood pressure control within white and non-white subgroups (308 white and 328 non-white). | **Group 1 Intervention:**  Usual care  **Group 2 Intervention:** Tailored behavioral self-management intervention administered via telephone by a nurse every other month. The nurse underwent training in aspects of motivational interviewing and in the specific procedures developed for this study. **Group 3 Intervention:** Home BP monitor Patients were asked to take their BP 3 times per week, on 3 separate days at the same time of the day, and to record their values . Patients were asked to email their log in every 2 months to the study coordinator. **Group 4 Intervention:** Both behavioural intervention and home blood pressure monitoring | **Mean age group 1:** 63 (SD 12)  **Mean age group 2:** 59.0 (SD 12)  **Mean age group 3:**  **% male group 1:**  **% male group 2:**  **% male group 3:**  **Ethnicity group 1:** White and non-white | **Primary outcome:**  SBP decreases of 5.3 to 5.7 mm Hg in non-white partipcants in all intervention groups compared to usual care  **p =** < 0.05 |
| {Rosal, 2011 #132} | **Study design:** RCT **CCM elements:** SMS+CIS **Chronic condition:** Diabetes  **Country:** USA **Setting:** Community-based care **Duration of intervention in (months):** 12.0  **Number of patients:** 252.0 **Number of health professionals:** **Study Aim:** To test whether a theory-based, literacy, and culturally tailored self-management intervention, Latinos en Control, improves glycemic control among low-income Latinos with type 2 diabetes. | **Group 1 Intervention:**  Usual care  **Group 2 Intervention:** Group-based intervention consisted of 12 weekly and 8 monthly sessions and targeted knowledge attitudes, and self-management behaviors. Patients received both brief personalized counseling, with feedback regarding their logs and meter data. Participants received reminder calls on the evening before each session. Providers of patients who exceeded safety threshold received an e-mail notification that contained a graph of the patient's glucose values downloaded from their meter. | **Mean age group 1:** 55% aged >=55  **Mean age group 2:** 52.5% aged >=55  **% male group 1:** 25.0  **% male group 2:** 21.8  **Ethnicity group 1:** Hispanic/Latino | **Primary outcome:**  HbA1c NS  **p =** |
| {Madsen, 2010 #99} | **Study design:** RCT **CCM elements:** SMS **Chronic condition:** Hypertension  **Country:** Denmark **Setting:** Primary care **Duration of intervention in (months):** 6.0  **Number of patients:** 236.0 **Number of health professionals:** **Study Aim:** The purpose of the present study was to compare the costs of home blood pressure (BP) telemonitoring (HBPM) with the costs of conventional office BP monitoring. | **Group 1 Intervention:**  Usual care  **Group 2 Intervention:** Patients were provided BP equipment. BP measurements were transferred to a central server. Each patient could assess their own BP on a website about lifestyle modification and medication adherence. Patients could communicate with their GP by e-mail. For patients with no internet access, the PDA could record and send spoken messages to the general practitioner, who could respond by written messages to the PDA | **Mean age group 1:** 57 (SD 12)  **Mean age group 2:** 55.0 (SD 12)  **% male group 1:** 50.0  **% male group 2:** 49.0  **Ethnicity group 1:** Not reported | **Primary outcome:**  For systolic ABP, the difference in cost-effectiveness ratio between groups was 256 Danish kroner (DKK)/mmHg (95% UI-860 to 4544) NS  **p =** |
| {Heinrich, 2010 #75} | **Study design:** RCT **CCM elements:** DS+SMS **Chronic condition:** Diabetes  **Country:** Netherlands **Setting:** Primary care **Duration of intervention in (months):** 24.0  **Number of patients:** 584.0 **Number of health professionals:** 33.0 **Study Aim:** The present study assessed the effects of a Motivational Interviewing (MI) based counselling training for nurses on clinical, behavioural and process outcomes among diabetes type 2 patients. | **Group 1 Intervention:**  Usual care and access to website  **Group 2 Intervention:** Two 5-h sessions were organized to train nurses in the an adapted for of MI and the use of the education tool Three months after intervention, nurses received written feedback on two audio-taped consultations. Nurses were supposed to apply the new couselling style during standard quarterly consultations with their patients. During the two years of the study, nurses and patients from the experimental and control group had access to a web-based education programme | **Mean age group 1:** 59 (SD 5.3)  **Mean age group 2:** 59.0 (SD 5.3)  **% male group 1:** 55.1  **% male group 2:** 55.1  **Ethnicity group 1:** Not reported | **Primary outcome:**  Vegetable intake NS  **p =** |
| {Eakin, 2010 #48} | **Study design:** RCT **CCM elements:** SMS DSD  **Chronic condition:** Diabetes or hypertension  **Country:** Australia **Setting:** Primary Care **Duration of intervention in (months):** 18.0  **Number of patients:** 434 **Number of health professionals:** **Study Aim:** To examine the maintenance of behavioral changes 6 months following a telephone-delivered physical activity and diet intervention. | **Group 1 Intervention:**  Usual care  **Group 2 Intervention:** Telephone delivered physical activity and dietry intervention | **Mean age group 1:** 57.8 (SD 11.9)  **Mean age group 2:** 58.7 (SD 11.7)  **% male group 1:** 40.3%  **% male group 2:** 37.7%  **Ethnicity group 1 & 2:** Caucasian | **Primary outcome:**  Mean physical activity 6 months post study NS  **p =** |
| {Weber, 2010 #182} | **Study design:** RCT **CCM elements:** DSD, SMS **Chronic condition:** Hypertension  **Country:** USA **Setting:** Primary Care **Duration of intervention in (months):** 9  **Number of patients:** 179 **Number of health professionals:** **Study Aim:** Pharmacist-physician comanagement of hypertension has been shown to improve office blood pressures (BPs). We sought to describe the effect of such a model on 24-hour ambulatory BPs. | **Group 1 Intervention:**  Usual care  **Group 2 Intervention:** Pharmacist-physician collaborative management of hypertension workshop and, two follow-up telephone calls two and four weeks after the workshop. Pharmacists helped patients identify barriers to BP control, counselled on lifestyle and and dietary modifications and adjusted antihypertensive therapy in collaboration with patient's primary care provider | **Mean age group 1:** 61.9 (SD 11.3)  **Mean age group 2:** 59.6 (SD 13)  **% male group 1:** 46.2  **% male group 2:** 41.6  **Ethnicity group 1:** 94.9% Caucasian  **Ethnicity group 2:** 88.1% Caucasian | **Primary outcome:**  Ambulatory BP profile SIG  **p =** 0.001 |
| {van de Steeg-van Gompel, 2010 #168} | **Study design:** RCT **CCM elements:** DS+DSD+SMS **Chronic condition:** Hypertension  **Country:** Netherlands **Setting:** Community-based care **Duration of intervention in (months):** 6.0  **Number of patients: Number of health professionals:** **Study Aim:** The aim of the study was to compare the effectiveness of intensive support for implementation of this Medication Event Monitoring System (MEMS) tool in community pharmacies compared with minimal implementation support. | **Group 1 Intervention:**  Control pharmacies received a manual  **Group 2 Intervention:** Educational manual, two interactive educational meetings; and at least two reminder and support telephone calls by a research assistant. The GP decided whether any medication changes were to be made on the basis of the electronically measured medication adherence Medication Event Monitoring System (MEMS) | **Mean age group 1:** 66 (SD 10.6)  **Mean age group 2:** 63.2 (SD 9.199)  **% male group 1:** 41.7  **% male group 2:** 54.0  **Ethnicity group 1:** Not reported | **Primary outcome:**  The number of patients included for electronic monitoring NS  **p =** |
| {Dyson, 2010 #46} | **Study design:** RCT **CCM elements:** SMS **Chronic condition:** Diabetes  **Country:** UK **Setting:** Community-based care **Duration of intervention in (months):** 6.0  **Number of patients:** 42.0 **Number of health professionals:** **Study Aim:** To develop a video-based lifestyle education programme for people newly diagnosed with type 2 diabetes and to evaluate changes in knowledge, biomedical indices and quality of life. | **Group 1 Intervention:**  Usual care  **Group 2 Intervention:** 3 lifestyle videos. All subjects in the study received usual medical care from their primary care physician, including education about lifestyle management of type 2 diabetes from a practice nurse | **Mean age group 1:** 63 (SD 9.5)  **Mean age group 2:** 58.6 (SD 9.199)  **% male group 1:** 47.6  **% male group 2:** 38.1  **Ethnicity group 1:** Not reported | **Primary outcome:**  The primary outcome was change in diabetes knowledge SIG  **p =** 0.000 |
| {Abramson, 2010 #195} | **Study design:** RCT **CCM elements:** DS **Chronic condition:** Asthma  **Country:** Australia **Setting:** Primary care **Duration of intervention in (months):** 12.0  **Number of patients:** 305.0 **Number of health professionals:** **Study Aim:** To determine whether spirometry with regular medical review improves the quality of life or other health outcomes among patients with asthma or chronic obstructive pulmonary disease (COPD) managed in general practice. | **Group 1 Intervention:**  Spirometry and regular follow-up  **Group 2 Intervention:** Spirometry only **Group 3 Intervention:** Usual care | **Mean age group 1:** 54 (SD )  **Mean age group 2:** 60 (SD )  **Mean age group 3:** 58 (SD )  **% male group 1:** 29  **% male group 2:** 38  **% male group 3:** 34  **Ethnicity group 1:** Not reported | **Primary outcome:**  SF36 NS  **p =** |
| {Mancuso, 2010 #102} | **Study design:** RCT **CCM elements:** SMS **Chronic condition:** Asthma  **Country:** USA **Setting:** Primary care **Duration of intervention in (months):** 27.0  **Number of patients:** 180.0 **Number of health professionals:** **Study Aim:** To improve asthma knowledge and self-efficacy and to assess effects in patients with depressive symptoms. | **Group 1 Intervention:**  At enrollment, controls received 3 brochures from the American Lung Association providing facts about asthma, how to modify the home to minimize triggers, and how to use peak flow meter.  **Group 2 Intervention:** Patient made a contract to adopt a behaviour ro imporve asthma, a workoo and weekly reinforcement | **Mean age group 1:** 43 (SD 13)  **Mean age group 2:** 42.0 (SD 14)  **% male group 1:** 16.0  **% male group 2:** 17.0  **Ethnicity group 1:** Caucasian | **Primary outcome:**  Asthma quality of life (Asthma Quality of Life Questionnaire) NS  **p =** |
| {Simon, 2010 #143} | **Study design:** RCT **CCM elements:** CIS+SMS **Chronic condition:** Diabetes  **Country:** USA **Setting:** Community-based care **Duration of intervention in (months):**  **Number of patients:** 1200.0 **Number of health professionals:** **Study Aim:** The study's objective was to assess the effects of automated telephone outreach with speech recognition (ATO-SR) on diabetes-related testing. | **Group 1 Intervention:**  Usual care  **Group 2 Intervention:** The automated system offered a live telephone call back to assist in scheduling tests and also offered to send participants 1)a voucher to allow provider to waive co-payment for a detailed eye exam. 2)Educational nutrition video 3)a cookbook 4)a pill box | **Mean age group 1:** 52 (SD )  **Mean age group 2:** 50.0 (SD )  **% male group 1:** 41.0  **% male group 2:** 64.0  **Ethnicity group 1:** Not reported | **Primary outcome:**  The primary outcome was retinopathy testing NS  **p =** |
| {Baig, 2010 #14} | **Study design:** RCT **CCM elements:** CR+DSD **Chronic condition:** Hypertension  **Country:** USA **Setting:** Community-based care **Duration of intervention in (months):** 4.0  **Number of patients:** 100.0 **Number of health professionals:** **Study Aim:** To measure the effect of faith community nurse referrals versus telephone-assisted physician appointments on blood pressure control among persons with elevated blood pressure at health fairs. | **Group 1 Intervention:**  Telephone assistance with making a physician appointment  **Group 2 Intervention:** Referal to a faith community nurse. Nurses followed standard protocol for referring participants with hypertension. Patients were introduced to nurse, given a letter with her contact information and encouraged to see her at least once during the next two weeks | **Mean age group 1:** 57 (SD 13)  **Mean age group 2:** 58.0 (SD 9)  **% male group 1:** 36.0  **% male group 2:** 28.0  **Ethnicity group 1:** Hispanic/Latino | **Primary outcome:**  SBP SIG  **p =** 0.040 |
| {Bennett, 2010 #18} | **Study design:** RCT **CCM elements:** SMS **Chronic condition:** Hypertension  **Country:** USA **Setting:** Community-based care **Duration of intervention in (months):** 3.0  **Number of patients:** 101.0 **Number of health professionals:** **Study Aim:** We conducted a 12-week randomized controlled trial to evaluate the short-term efficacy of a web-based weight loss intervention among 101 primary care patients with obesity and hypertension. | **Group 1 Intervention:**  A copy of the 'Aim for a Healthy Weight" materials, published by the National Heart Lung and Blood Institiute  **Group 2 Intervention:** Web-based intevention. iOTA provides participants with a series of tailored obesogenic behavior changes goals that are subjected to regular self monitoring. A health coach helped them to select four behavior change goals using algorithm that prioritized bahaviors in need of change, for which the participant had high self-efficacy for change A health coach conducted two, 20-min motivational coaching sessions in person (baseline and week 6), and two, 20-min biweekly sessions via telephone | **Mean age group 1:** 55 (SD 8.9)  **Mean age group 2:** 54.4 (SD 7.4)  **% male group 1:** 46.0  **% male group 2:** 58.8  **Ethnicity group 1:** Caucasian | **Primary outcome:**  Weight Difference between conditions -2.56 (95% CI -3.60, -1.53) SIG  **p =** Not reported |
| {Rinfret, 2009 #131} | **Study design:** RCT **CCM elements:** CIS+SMS+DSD **Chronic condition:** Hypertension  **Country:** Canada **Setting:** Primary care **Duration of intervention in (months):** 12.0  **Number of patients:** 223.0 **Number of health professionals:** **Study Aim:** Our multidisciplinary research group developed an information technology (IT)-supported management program to facilitate BP and adherence data circulation between patients and primary care healthcare providers, without the need for high intensity and costly personnel interventions. We tested the hypothesis that this multifaceted program would improve mean 24-hour BP levels. | **Group 1 Intervention:**  Usual care  **Group 2 Intervention:** Multidisciplinary information technology-supported program. Data faxed monthly to physicians, pharmacists and study nurses. Educational booklets, a digital home BP monitor, a log book and access to a telephone-linked IT-supported management program. Nurses could contact subjects to inquire about BP and adherence difficulties and to deal with them or refer subjects to their physician or pharmacist as specified in the study algorithm. | **Mean age group 1:** 57 (SD 13)  **Mean age group 2:** 55.0 (SD 11)  **% male group 1:** 54.5  **% male group 2:** 54.1  **Ethnicity group 1:** Not reported | **Primary outcome:**  SBP SIG  **p =** 0.001 |
| {Cavanaugh, 2009 #32} | **Study design:** RCT **CCM elements:** SMS+DSD **Chronic condition:** Diabetes  **Country:** USA **Setting:** Primary care **Duration of intervention in (months):** 6.0  **Number of patients:** 198.0 **Number of health professionals:** **Study Aim:** This study evaluated the impact of providing literacy- and numeracysensitive diabetes care within an enhanced diabetes care program on A1C and other diabetes outcomes. | **Group 1 Intervention:**  Control - enhanced diabetes care program  **Group 2 Intervention:** Enhanced diabetes care program plus a focus on addressing health lietarcy and numeracy. Use of the Diabetes Lieteracy and Numeracy Education Toolkit (DLNET) to promote self-management | **Mean age group 1:** 53 (SD )  **Mean age group 2:** 52.0 (SD )  **% male group 1:** 35.0  **% male group 2:** 37.0  **Ethnicity group 1:** Not reported | **Primary outcome:**  HbA1c NS  **p =** |
| {Gary, 2009 #62} | **Study design:** RCT **CCM elements:** DS+DSD+SMS **Chronic condition:** Diabetes  **Country:** USA **Setting:** Managed care organisation **Duration of intervention in (months):** 24.0  **Number of patients:** 542.0 **Number of health professionals:** **Study Aim:** To assess a culturally tailored intervention conducted by an Nurse Case Manager (NCM)/CHW team on frequency of ER visits in urban African Americans with type 2 DM. | **Group 1 Intervention:**  Mailings and telephone calls every 6 months to remind participants about preventive screening  **Group 2 Intervention:** NCM and CHW visits. CHW worker conducted home visits, during which they conducted a random blood glucose test, monitored BP and gave praticipants immediate feedback on the results. They provided health education, problem solving and follow-up | **Mean age group 1:** 56 (SD 11)  **Mean age group 2:** 59.0 (SD 11)  **% male group 1:** 26.0  **% male group 2:** 27.0  **Ethnicity group 1:** African American | **Primary outcome:**  HbA1c NS  **p =** |
| {Gutschall, 2009 #71} | **Study design:** RCT **CCM elements:** SMS **Chronic condition:** Diabetes  **Country:** USA **Setting:** Primary care **Duration of intervention in (months):** 2.0  **Number of patients:** 109.0 **Number of health professionals:** **Study Aim:** The purpose of the present study was to evaluate the change in outcomes following a behavioural intervention which promoted lower-GI foods among adults with diabetes. | **Group 1 Intervention:**  Delayed control group (starting after 9 weeks).Nine weekly group sessions lasting 1.5 to 2 h heach.  **Group 2 Intervention:** Nine weekly group sessions lasting 1.5 to 2 h each. The 9-week curriculum progressed through the topics of self-monitoring food intake and blood glucose, monitoring portion sizes of food to control carbohydrate intake, goal-setting prinicples (and other topics) | **Mean age group 1:** 60 (SD 7.3)  **Mean age group 2:** 58.6 (SD 7.7)  **% male group 1:** 47.9  **% male group 2:** 40.0  **Ethnicity group 1:** Caucasian | **Primary outcome:**  During the initial 9-week treatment-control period, the mean change in GL (glycaemic load) for the immediate group was not significantly different from the delayed group NS  **p =** |
| {Olson, 2009 #268} | **Study design:** RCT **CCM elements:** CIS+SMS **Chronic condition:** Heart disease  **Country:** USA **Setting:** Managed care organisation **Duration of intervention in (months):** 24.0  **Number of patients:** 421.0 **Number of health professionals:** **Study Aim:** To evaluate whether patients with coronary artery disease (CAD) discharged from the Clinical Pharmacy Cardiac Risk Service (CPCRS) would maintain their lipid goals with use of an electronic laboratory reminder system. | **Group 1 Intervention:**  Usual care  **Group 2 Intervention:** Patients continued to receive care from Clinical Pharmacy Cardiac Risk Service (CPCRS). Clinical pharmacy specialists telephone patients to review the results of their annual fasting lipid profile, BP, and medication and adherence; and making medication adjustments to maintain treatment goals. The specialists ordered follow-up laboratory tests through the electronic medical record. All patients were mailed letters informing them or their results, and received laboratory reminder letters | **Mean age group 1:** 71 (SD 9)  **Mean age group 2:** 72.0 (SD 10)  **% male group 1:** 77.0  **% male group 2:** 71.0    **Ethnicity group 1:** Not reported | **Primary outcome:**  Lipids NS **p =** |
| {Powers, 2009 #128} | **Study design:** RCT **CCM elements:** SMS+DSD+CIS **Chronic condition:** Diabetes  **Country:** USA **Setting:** Primary care **Duration of intervention in (months):** 24.0  **Number of patients:** 588.0 **Number of health professionals:** **Study Aim:** We evaluated the effect of a tailored hypertension self-management intervention on the unintended targets of glycosylated hemoglobin (HbA1c) and low-density lipoprotein cholesterol (LDL-C). | **Group 1 Intervention:**  Usual care  **Group 2 Intervention:** Hypertension self-management intervention. The nurse telephoned patients within 1 week of randomization and then every 2 months over 24 months to deliver the intervention. At each call, the nurse delivered scripted information drawn from 9 educational and behavioural modules. | **Mean age group 1:** 64 (SD 10.8)  **Mean age group 2:** 63.0 (SD 10.8)  **% male group 1:** 99.0  **% male group 2:** 98.0  **Ethnicity group 1:** Not reported | **Primary outcome:**  HbA1c SIG  **p =** 0.030 |
| {Eakin, 2009 #47} | **Study design:** RCT **CCM elements:** SMS+DSD **Chronic condition:** Diabetes  **Country:** Australia **Setting:** Primary care **Duration of intervention in (months):** 12.0  **Number of patients:** 434.0 **Number of health professionals:** **Study Aim:** To examine the maintenance of behavioral changes of a telephone-delivered physical activity and diet intervention. | **Group 1 Intervention:**  Usual care  **Group 2 Intervention:** Telephone counselling intervention for physical activity and diet by trained counsellors. Participants received an intervention workbook along with a pedometer, a self-monitoring form, and an exercise band. | **Mean age group 1:** 58 (SD 11.9)  **Mean age group 2:** 58.7 (SD 11.7)  **% male group 1:** 40.3  **% male group 2:** 39.7  **Ethnicity group 1:** Caucasian | **Primary outcome:**  Mean physical activity 12 months NS  **p =** |
| {McLean, 2008 #106} | **Study design:** RCT **CCM elements:** DSD+SMS+DS **Chronic condition:** Hypertension  **Country:** Canada **Setting:** Community-based care **Duration of intervention in (months):** 6.0  **Number of patients:** 227.0 **Number of health professionals:** **Study Aim:** This study aimed to determine the efficacy of a community-based multidisciplinary intervention on BP control in patients with diabetes mellitus. | **Group 1 Intervention:**  Usual care. Patients in UC group received the same BP wallet card with their BP measures documented, a pamphlet on diabetes and general diabetes couseling from a nurse or a pharmacist. UC patients received telephone follow-up at 12 wks and no other follow-up until 24 wks  **Group 2 Intervention:** Cardiovascular risk reduction couseling was provided by a nurse-pharmacist team using a hypertension education brochure and cardiovascular risk reduction couseling brochure | **Mean age group 1:** 64 (SD 12.7)  **Mean age group 2:** 66.2 (SD 11.3)  **% male group 1:** 54.5  **% male group 2:** 65.2    **Ethnicity group 1:** Not reported | **Primary outcome:**  SBP SIG  **p =** 0.008 |
| {Amoako, 2008 #10} | **Study design:** RCT **CCM elements:** SMS **Chronic condition:** Diabetes  **Country:** USA **Setting:** Primary care **Duration of intervention in (months):** 2.0  **Number of patients:** 68.0 **Number of health professionals:** **Study Aim:** This study evaluated a telephone intervention for African American women to reduce uncertainty (through problem-solving strategies, information, cognitive reframing, and improved patient-provider communication) and measure its effects on diabetes self-care and psychosocial adjustment. | **Group 1 Intervention:**  Usual care  **Group 2 Intervention:** The intervention was designed to address issues and concerns related to diabetes self-care. Theories of counseling and behavior change guided the interactive process used in delivery of the intervention Participants were provided with problem-solving strategies, information, and resources aimed at increasing diabetes knowledge and self-care skills, as well as strategies to improve patient-provider communication | **Mean age group 1 & 2:** 61 (SD 9.5)  **% female group 1:** 100%  **% female group 2:** 100%  **Ethnicity group 1:** African American | **Primary outcome:**  The experimental group reported increased participation in exercise (self-care component) SIG  **p =** 0.001 |
| {Jerant, 2008 #82} | **Study design:** RCT **CCM elements:** SMS+DSD+DS **Chronic condition:** Arthritis  **Country:** USA **Setting:** Community-based care **Duration of intervention in (months):** 6.0  **Number of patients:** 415.0 **Number of health professionals:** **Study Aim:** We examined the effects of a home-based variant of the Chronic Disease Self-Management Program on self-efficacy, and explored the moderating effects of perceived control over self-management (PCSM). | **Group 1 Intervention:**  Usual care  **Group 2 Intervention:** The original CDSMP is privided by pairs of non-healthcare professionals, called facilitators, who have prsonal experience in chronic health conditions. The CDSMP aims to bloster patient self-efficacy for self-managing their chronic medical conditions, regardless of specific diagnosis **Group 3 Intervention:** HIOH intervention was essentially identical to DSMP in content. However, it differed from the CDSMP in terms of delivery process, since one trained layperson provided the intervention to one participant, and setting, since it was provided via telephone | **Mean age group 1:** 60 (SD 11.7)  **Mean age group 2:** 59.8 (SD 11.2)  **Mean age group 3:** 61.2 (SD 11.6)  **% male group 1:** 25.0  **% male group 2:** 22.0  **% male group 3:** 22.0  **Ethnicity group 1:** Non-Hispanic Caucasian | **Primary outcome:**  Only the home intervention had a significant self-efficacy-enhancing effect (Wald test, x2=13.8, p=0.008; effect size=0.3). SIG  **p =** 0.008 |
| {Thiebaud, 2008 #160} | **Study design:** CBA **CCM elements:** SMS+DSD **Chronic condition:** Diabetes  **Country:** USA **Setting:** Community-based care **Duration of intervention in (months):** 24.0  **Number of patients:** 5169.0 **Number of health professionals:** **Study Aim:** The purpose of this study was to evaluate the effect of telephonic care management within a diabetes disease management program on adherence to treatment with hypoglycemic agents, ACE inhibitors (ACEIs), angiotensin receptor blockers (ARBs), statins, and recommended laboratory tests in a Medicaid population. | **Group 1 Intervention:**  Usual care  **Group 2 Intervention:** Telephone disease management model that reinforced goals already established between the health professional and the patient. Delivered by nurse care managers responsible for tailoring treatment plans to each patient. Nurses contacted the provider's office directly with information that might result in a new medication of test order. Care managers also had an indirect influence on adherence to guidelines by educating and empowering patients to partner with their provider | **Mean age group 1:** 51 (SD )  **Mean age group 2:** 52.8 (SD )  **% male group 1:** 30.0  **% male group 2:** 21.7  **Ethnicity group 1:** Caucasian | **Primary outcome:**  Non users' increased their overall hypoglycemic use by 0.7 scripts SIG  **p =** 0.001 |
| {Goodman, 2008 #67} | **Study design:** RCT **CCM elements:** SMS+DS **Chronic condition:** Heart disease  **Country:** UK **Setting:** Community-based care **Duration of intervention in (months):** 9.0  **Number of patients:** 188.0 **Number of health professionals:** **Study Aim:** The 'Fit For Surgery' programme was based on previous studies suggesting improvement in risk factors contributing to coronary disease while patients wait for cardiac surgery. We evaluated our nurse-led programme in a randomised controlled trial with 188 patients. | **Group 1 Intervention:**  Usual care  **Group 2 Intervention:** Nurse-led programmme of support and lifestyle management for patients awaiting cardiac surgery. The patients also had a copy of the manual evaluated in the pilot study and the nurses guided them through the sections covering risk factors, preparation for surgery and what to do if they encounter chest pain. Nurses were trained in motivational interviewing techniques | **Mean age group 1:** 66 (SD )  **Mean age group 2:** 63.7 (SD )  **% male group 1:** 86.0  **% male group 2:** 76.6  **Ethnicity group 1:** Not reported | **Primary outcome:**  HAD (Hospital Anxiety and Depression scale) NS  **p =** |
| {Green, 2008 #69} | **Study design:** RCT **CCM elements:** SMS+CIS+DSD **Chronic condition:** Hypertension  **Country:** USA **Setting:** Community-based care **Duration of intervention in (months):** 12.0  **Number of patients:** 778.0 **Number of health professionals:** **Study Aim:** To determine if a new model of care that uses patient Web services, home blood pressure (BP) monitoring, and pharmacist-assisted care improves BP control. | **Group 1 Intervention:**  Usual care  **Group 2 Intervention:** Patients were given a home BP monitor and trained to use it. Also they received training on how to use the Website **Group 3 Intervention:** Those assigned to home BP monitoring and Web training plus pharmacist care were told a pharmacist would be assisting them to improve their BP control via home BP monitoring and Web communications Clinical pharmacists with experience and time separate from front-line customer service to assist with team-based care management activities. | **Mean age group 1:** 59 (SD 8.5)  **Mean age group 2:** 59.5 (SD 8.300)  **Mean age group 3:** 59.3 (SD 8.6)  **Mean age group 4:** (SD )  **% male group 1:** 45.3  **% male group 2:** 54.1  **% male group 3:** 44.1  **Ethnicity group 1:** Caucasian | **Primary outcome:**  BP SIG  **p =** 0.001 |
| {Madsen, 2008 #100} | **Study design:** RCT **CCM elements:** SMS **Chronic condition:** Hypertension  **Country:** Denmark **Setting:** Primary Care **Duration of intervention in (months):** 6  **Number of patients:** 236 **Number of health professionals:** **Study Aim:** To compare the effectiveness of antihypertensive treatment based on telemonitoring of home blood pressure and conventional monitoring of office blood pressure. | **Group 1 Intervention:**  Usual care  **Group 2 Intervention:** Home BP monitoring and upload of results, and management by GP via telemedicine | **Mean age group 1:** 56.7 (SD 11.6)  **Mean age group 2:** 55 (SD 11.7)  **% male group 1:** 53.0  **% male group 2:** 48.7    **Ethnicity group 1:** Not reported | **Primary outcome:**  Systolic daytime ambulatory BP monitoring change NS  **p =** 0.139 |
| {Zautra, 2008 #192} | **Study design:** RCT **CCM elements:** SMS **Chronic condition:** Arthritis  **Country:** USA **Setting:** Community-based care **Duration of intervention in (months):** 6  **Number of patients:** 144.0 **Number of health professionals:** **Study Aim:** This research examined whether cognitive behavioral therapy and mindfulness interventions that target responses to chronic stress, pain, and depression reduce pain and improve the quality of everyday life for adults with rheumatoid arthritis (RA). | **Group 1 Intervention:**  Presentations in which general information about RA and related thems were presented  **Group 2 Intervention:** CBT for pain **Group 3 Intervention:** Mindfulness meditation and emotion regulation therapy **Group 4 Intervention:** | **Mean age group 1:** 51.9 (SD 13.5)  **Mean age group 2:** 53.6 (SD 12.1)  **Mean age group 3:** 51.7 (SD 14)  **% male group 1:** 22.7  **% male group 2:** 30.8  **% male group 3:** 42.6  **Ethnicity group 1:** Most Caucasian | **Primary outcome:**  Daily pain NS  **p =** |
| {Carter, 2008 #31} | **Study design:** RCT **CCM elements:** DS+DSD **Chronic condition:** Hypertension  **Country:** USA **Setting:** Primary care **Duration of intervention in (months):** 9.0  **Number of patients:** 179.0 **Number of health professionals:** 32.0 **Study Aim:** The purpose of the present study was to evaluate a physician/pharmacist collaborative model to improve BP control. | **Group 1 Intervention:**  Usual care  **Group 2 Intervention:** Physician/pharmacist collaboration to improve blood pressure. The pharmacist assessed the patient's regimen, suggested a goal BP value, and provided recommendations to improve BP control. The pharmacist educated all patients using written information from NHLBI and/or taught them to perform home monitoring. Pharmacists were encouraged to attend each clinic visit, and they were encouraged to initiate additional visits or telephone contacts if BP remained uncontrolled. The results of these interviews served as the basis for patient-specific recommendations and feedback for the physician **.** | **Mean age group 1:** 62 (SD 11.3)  **Mean age group 2:** 59.6 (SD 13 .7)  **% male group 1:** 46.2  **% male group 2:** 41.6  **Ethnicity group 1:** Caucasian | **Primary outcome:**  BP control 89.1% controlled in intervention and 52.9% controlled in control group (adjusted OR 8.9 (95% CI3.8, 20.7)  **p =** 0.001 |
| {Christian, 2008 #34} | **Study design:** RCT **CCM elements:** SMS+DS **Chronic condition:** Diabetes  **Country:** USA **Setting:** Primary care **Duration of intervention in (months):** 12.0  **Number of patients:** 310.0 **Number of health professionals:** **Study Aim:** Our objective was to test the effect of physicians providing brief health lifestyle counseling to patients with type 2 diabetes mellitus during usual care visits. | **Group 1 Intervention:**  Printed health education materials  **Group 2 Intervention:** The purposes of feedback were 1) enhance participant's motivation to increase physical activity and reduce caloric intake 2) indentify barriers to lifestyle change 3) povide tailored couseling suggestions. Prior to baseline clinic visit, intervention patients read their report and listed 2 or 3 dietary and/or physical activity self-management goals they wanted to achieve. The computer expert system generated a 4-5 page individualized tailored report, which provided feedback addressing participant-identified barriers to improving their physical activity and diet | **Mean age group 1:** 53 (SD 10.7)  **Mean age group 2:** 53.0 (SD 11.3)  **% male group 1:** 32.0  **% male group 2:** 35.0  **Ethnicity group 1:** Hispanic/Latino | **Primary outcome:**  Weight NS  **p =** |
| {Cook, 2007 #36} | **Study design:** CBA **CCM elements:** SMS+CIS **Chronic condition:** Osteoporosis  **Country:** USA **Setting:** Community-based care **Duration of intervention in (months):** 4.0  **Number of patients:** 402.0 **Number of health professionals:** **Study Aim:** This study was a nonrandomized trial of a telehealth program that used motivational interviewing and cognitive- behavioral techniques to improve osteoporosis medication adherence. | **Group 1 Intervention:**  All participants received the intervention. To evaluate program effectiveness, participants' adherence rates were compared with national baseline data on osteoporosis medication adherence. ScriptAssist telephonic couseling program. All interventions were delivered telephonically by 1 of 4 registered nurses at the ScriptAssisst  **Group 2 Intervention:** Comparison to results of 4 published trials | **Mean age group 1:** 67 (SD 12.5)  **Mean age group 2:** (SD )  **% male group 1:** 4.0  **% male group 2:**  **Ethnicity group 1:** Not reported | **Primary outcome:**  Pharmacy measure of adherence SIG  **p =** 0.009 |
| {Artinian, 2007 #13} | **Study design:** RCT **CCM elements:** SMS+CIS **Chronic condition:** Hypertension  **Country:** USA **Setting:** Community-based care **Duration of intervention in (months):** 12.0  **Number of patients:** 387.0 **Number of health professionals:** **Study Aim:** To test the hypothesis that individuals who participate in usual care (UC) plus blood pressure (BP) telemonitoring (TM) will have a greater reduction in BP from baseline to 12-month follow-up than would individuals who receive UC only. | **Group 1 Intervention:**  Usual care  **Group 2 Intervention:** During a prescheduled appointment, the intervention nurse delivered the BP monitor and TM link device (device that links BP monitor to the telephone)to the participants home. TM participants were asked to telephonically send their BP reading to the intervention nurse and their care providers once a week during the first 3 months of the study, then once a month between the 4- and 12-month follow-ups. | **Mean age group 1:** 60 (SD 12.3)  **Mean age group 2:** 59.1 (SD 13)  **% male group 1:** 30.1  **% male group 2:** 41.2  **Ethnicity group 1:** Not reported | **Primary outcome:**  SBP SIG  **p =** 0.040 |
| {Zauszniewski, 2007 #191} | **Study design:** RCT **CCM elements:** SMS **Chronic condition:** Arthritis  **Country:** USA **Setting:** Community-based care **Duration of intervention in (months):** 3.0  **Number of patients:** 176.0 **Number of health professionals:** **Study Aim:** This clinical trial examined changes in affect, behavior, and cognition in 176 chronically ill elders who were randomly assigned to Resourcefulness Training (RT), Acceptance Training (AT), or Diversional Activities (DA). | **Group 1 Intervention:**  Diversional Activities (DA)  **Group 2 Intervention:** Resoucefulness Training (RT) **Group 3 Intervention:** Acceptance Training (AT) | **Mean age group 1:** 83 (SD )  **Mean age group 2:** 83.0 (SD )  **Mean age group 3:** 83.0 (SD )  **Mean age group 4:** (SD )  **% male group 1:** 21.0  **% male group 2:** 21.0  **% male group 3:** 21.0  **Ethnicity group 1:** Caucasian | **Primary outcome:**  Affect SIG  **p =** 0.030 |
| {Dolovich, 2007 #44} | **Study design:** RCT **CCM elements:** DS+SMS **Chronic condition:** Asthma  **Country:** Canada **Setting:** Community-based care **Duration of intervention in (months):** 1.0  **Number of patients: Number of health professionals:** 64.0 **Study Aim:** To determine if an educational program designed for community pharmacists to help patients self manage their asthma could improve pharmacists abilities to facilitate asthma treatment plans | **Group 1 Intervention:**  Control group. Pharmacists received a delayed asthma education program (AEP)(i.e. received the intervention after primary study outcomes were measured).  **Group 2 Intervention:** Pharmacists receive asthma education program (AEP). The AEP cosists of a one-day workshop and two follow-up telephone calls. A tool-kit of resources. Skill-building topics focused on how to introduce the concept of asthma self-management with patients, a structured interview process for assessing patients and how to develop a patient's asthma action plan. | **Mean age group 1:** 42.1 (SD 9.8)  **Mean age group 2:** 42.8 (SD 13.6)  **% male group 1:** 35.7  **% male group 2:** 41.9  **Ethnicity group 1:** Not reported | **Primary outcome:**  The number of appropriate (defined a priori) action plans facilitated by the pharmacist was the primary outcome SIG  **p =** 0.000 |
| {Community Pharmacy Medicines Management Project Evaluation Team, 2007 #2} | **Study design:** RCT **CCM elements:** DSD+SMS+CIS **Chronic condition:** Heart disease  **Country:** UK **Setting:** Community-based care **Duration of intervention in (months):** 12.0  **Number of patients:** 1614.0 **Number of health professionals:** 226.0 **Study Aim:** This paper presents the results of a RCT to test the hypothesis that a comprehensive MEDMAN service would (i) increase the proportion of patients receiving treatment according to the National Service Framework(NSF) in England and Wales; (ii) improve overall patient health status; and (iii) be cost effective | **Group 1 Intervention:**  Usual care  **Group 2 Intervention:** The medicine management service was delivered from community pharmacists who had received training designed and delivered by the Centre for Pharmacy Post-Graduate Education. The intervention comprised an initial consultation informed by the extracted medical data supplied by the researchers. Further consultations were provided according to pharmacist-determined patient needs. | **Mean age group 1:** 69 (SD 9.1)  **Mean age group 2:** 68.7 (SD 9.199)  **% male group 1:** 70.6  **% male group 2:** 67.4  **Ethnicity group 1:** Not reported | **Primary outcome:**  Appropriate treatment [derived from the National Service Framework (NSF)], NS  **p =** |
| {Landon, 2007 #92} | **Study design:** CCT **CCM elements:** CIS+DS **Chronic condition:** Asthma  **Country:** USA **Setting:** Primary care **Duration of intervention in (months):**  **Number of patients:** 9658.0 **Number of health professionals:** **Study Aim:** To date, however, there has been no controlled evaluation of the effect of the Health Disparities Collaboratives on the quality of care. We report the results of a controlled, national evaluation of these collaboratives for the care of patients with three prevalent chronic medical conditions | **Group 1 Intervention:**  External control centers and Internal control centers (each intervention center also acted as internal control for another condition)  **Group 2 Intervention:** The Health Disparities Collaboratives. Briefly, each collaborative generally includes 20 or more community health centers and consists of a prework period, a kickoff meeting, and subsequent 2-day learning sessions. Regional and state infrastructure to provide technical assistance and information systems support to community health centers participating in the collaborative | **Mean age group 1:** 33 (SD )  **Mean age group 2:** 28.4 (SD )  **% male group 1:** 34.9  **% male group 2:** 36.5  **Ethnicity group 1:** Caucasian | **Primary outcome:**  Composite measure of quality of care (All three conditions) SIG  **p =** 0.001 |
| {Veenhof, 2006 #173} | **Study design:** RCT **CCM elements:** SMS **Chronic condition:** Osteoarthritis  **Country:** Netherlands **Setting:** Primary care **Duration of intervention in (months):** 16.0  **Number of patients:** 200.0 **Number of health professionals:** 87.0 **Study Aim:** To determine the effectiveness of a behavioral graded activity program (BGA) compared with usual care (UC;exercise therapy and advice) according to the Dutch guidelines for physiotherapy in patients with osteoarthritis (OA) of the hip and/or knee. The BGA intervention is intended to increase activity in the long term and consists of an exercise program with booster sessions, using operant treatment principles | **Group 1 Intervention:**  Physiotherapists in in both treatment groups received education on the allocated treatment  **Group 2 Intervention:** A behavioral treatment integrating the concepts of operant conditioning with execise therapy comprising booster sessions | **Mean age group 1:** 65 (SD 8.300)  **Mean age group 2:** 65.1 (SD 7.4)  **% male group 1:** 21.0  **% male group 2:** 25.0  **Ethnicity group 1:** Not reported | **Primary outcome:**  Pain assessed through visual analog scale (VAS) and through Western Ontario and Mcmaster Universitities Osteo arthritis Index (WOMAC) NS  **p =** |
| {Fretheim, 2006 #59} | **Study design:** RCT **CCM elements:** DS+CIS+DSD **Chronic condition:** Hypertension  **Country:** Norway **Setting:** Primary care **Duration of intervention in (months):** 12.0  **Number of patients:** 146.0 **Number of health professionals:** 501.0 **Study Aim:** We evaluated the effects of a tailored intervention to support the implementation of systematically developed guidelines for the use of antihypertensive and cholesterol-lowering drugs for the primary prevention of cardiovascular disease. | **Group 1 Intervention:**  Passive dissemination of guidelines  **Group 2 Intervention:** The intervention was initiated through an educational outreach visit by pharmacists. A software package was installed that extracted data and provided physicians with data on their performance of risk estimation, choice of antihypertensive drugs, achievement of treatment goals. The software package also included computerized reminders for an elevated BP or cholesterol or LDL cholestrol. Reminders for cardiovascular risk assessment | **Mean age group 1:** 61 (SD 13.5)  **Mean age group 2:** 61.2 (SD 13.5)  **% male group 1:** 48.3  **% male group 2:** 45.8  **Ethnicity group 1:** Not reported | **Primary outcome:**  Prescribing of thiazide for hypertension SIG  **p =** 0.001 |
| {Thom, 2006 #162} | **Study design:** RCT **CCM elements:** DS **Chronic condition:** Diabetes  **Country:** USA **Setting:** Primary care **Duration of intervention in (months):** 6.0  **Number of patients:** 429.0 **Number of health professionals:** 53.0 **Study Aim:** To develop and evaluate a brief cross-cultural curriculum for resident and practicing physicians based on a model of culturally competent physician behaviors, and to evaluate the training plus feedback compared to feedback alone with respect to changes in patient-reported physician behaviors, patient satisfaction,patient trust in his or her physician, and disease-specific patient health outcomes | **Group 1 Intervention:**  Feedback was provided to each physician via written report with and interpretation of the aggregated Patient-Reported  Physician Cultural Competence (PRPCC) scores with patients  **Group 2 Intervention:** Cultural competency training was provided to physicians at 2 sites. Teaching techniques included didactic presentations, group discussion, role-playing with learners critique, group exercises, use of trigger tapes, and handouts. Feedback was provided to each physician via written report with and interpretation of the aggregated PRPCC scores with patients | **Mean age group 1:** 62 (SD 11.4)  **Mean age group 2:** 54.9 (SD 11.6)  **% male group 1:** 36.8  **% male group 2:** 51.2  **Ethnicity group 1:** Hispanic/Latino | **Primary outcome:**  The primary outcome at 6 months was change in the Patient-Reported Physician Cultural Competence (PRPCC) score NS  **p =** |
| {Deakin, 2006 #41} | **Study design:** RCT **CCM elements:** SMS+DSD+CR **Chronic condition:** Diabetes  **Country:** UK **Setting:** Primary care **Duration of intervention in (months):** 14.0  **Number of patients:** 314.0 **Number of health professionals:** **Study Aim:** To develop a patient-centred, group-based self-management programme(X-PERT), based on theories of empowerment and discovery learning, and to assess the effectiveness of the programme on clinical, lifestyle and psychosocial outcomes | **Group 1 Intervention:**  In addition to routine care, the control group received diabetes education and review with prearranged individual appointments with a dietician, practice nurse and a general practitioner  **Group 2 Intervention:** The X-PERT Programme involved six weekly sessions, each lasting 2 h. The programme aimed to develop skills and build confidence, to enable patients to make informed decisions regarding their diabetes self-care If participants failed to attend a session they received a telephone reminder | **Mean age group 1:** 62 (SD 11)  **Mean age group 2:** 61.3 (SD 9.699)  **% male group 1:**  **% male group 2:**  **Ethnicity group 1:** Not reported | **Primary outcome:**  HbA1c SIG  **p =** 0.001 |
| {McGeoch, 2006 #104} | **Study design:** RCT **CCM elements:** SMS+DSD **Chronic condition:** COPD  **Country:** New Zealand **Setting:** Primary care **Duration of intervention in (months):** 12.0  **Number of patients:** 159.0 **Number of health professionals:** **Study Aim:** The objective of this study was to assess whether self-management plans administered in primary care have beneficial effects on quality of life, self-care behaviour and health outcomes in the long term for patients with COPD. | **Group 1 Intervention:**  Usual care  **Group 2 Intervention:** Standardized self-management plan education was deliveredin an individual session of 1-h duration from a practice nurse or respiratory educator in association with their general practitioner. Patients were instructed to make early contact with their general practice during exacerbations | **Mean age group 1:** 71 (SD 9.9)  **Mean age group 2:** 69.8 (SD 11.6)  **% male group 1:** 67.0  **% male group 2:** 52.0  **Ethnicity group 1:** Caucasian | **Primary outcome:**  The primary outcome was St. George's Respiratory Questionnaire NS  **p =** |
| {Davis, 2006 #38} | **Study design:** RCT **CCM elements:** SMS **Chronic condition:** COPD  **Country:** USA **Setting:** Community-based care **Duration of intervention in (months):** 2.0  **Number of patients:** 115.0 **Number of health professionals:** **Study Aim:** The purposes of this study were to 1) determine the effect of intervention on self-efficacy and 2) determine the relationship between domain-specific self-efficacy, walking performance, and symptom severity in patients with chronic obstructive pulmonary disease (COPD). | **Group 1 Intervention:**  The Dyspnea Self-Management Program (DM) was composed of 3 hours of dyspnea self-management educationand an individualized home walking prescription  **Group 2 Intervention:** DM-exposure included all components of DM and an additional four treadmill exercise sessions for 30 minutes once every 8 weeks **Group 3 Intervention:** DM-training included all components of DM and an additional twenty-four treadmill exercise sessions for 30 minutes three times a week for 8 weeks | **Mean age group 1:** 66 (SD 9)  **Mean age group 2:** 67.0 (SD 8)  **Mean age group 3:** 66.0 (SD 6.0)  **% male group 1:** 53.0  **% male group 2:** 44.0  **% male group 3:** 35.0  **Ethnicity group 1:** Not reported | **Primary outcome:**  Self-efficacy for walking NS  **p =** |
| {Gleeson-Kreig, 2006 #65} | **Study design:** RCT **CCM elements:** SMS **Chronic condition:** Diabetes  **Country:** USA **Setting:** Community-based care **Duration of intervention in (months):** 2.0  **Number of patients:** 58.0 **Number of health professionals:** **Study Aim:** The purpose of this study was to test the effect of keeping daily activity records on physical activity levels and self-efficacy for physical activity in adults with type 2 diabetes, and to examine the feasibility and acceptability of this intervention from the perspective of the participants | **Group 1 Intervention:**  Usual care  **Group 2 Intervention:** Self-monitoring of physical activity. The researcher explained how to keep daily activity records on the calender supplied to participants in the intervention group. In lieu of a stipend, individuals were given a pedometer, valued at $15 | **Mean age group 1:** 53 (SD )  **Mean age group 2:** 53.0 (SD )  **% male group 1:** 46.4  **% male group 2:** 50.0  **Ethnicity group 1:** Not reported | **Primary outcome:**  Self-efficacy SIG  **p =** 0.010 |
| {Lester, 2006 #93} | **Study design:** RCT **CCM elements:** DS+CIS **Chronic condition:** Heart disease  **Country:** USA **Setting:** Primary care **Duration of intervention in (months):** 12.0  **Number of patients:** 235.0 **Number of health professionals:** 14.0 **Study Aim:** We report the impact of a computer-assisted physician-directed intervention to improve secondary prevention of hyperlipidemia | **Group 1 Intervention:**  Usual care  **Group 2 Intervention:** The intervention consisted of a single 'FastTrack' e-mail sent to enrolled physicians on the first day of the study. Physicians also received a customized e-mail for each patient randomized to intervention status. Best practice decision support "One-click" In response to the clinical information provided, study physicians selected via single-click 1 or 3 mutually exclusive actions | **Mean age group 1:** 62 (SD 13.3)  **Mean age group 2:** 64.3 (SD 14.5)  **% male group 1:** 48.7  **% male group 2:** 51.7  **Ethnicity group 1:** Non-Caucasian | **Primary outcome:**  Change in hyperlipidemia prescriptions NS  **p =** |
| {Fritz, 2006 #60} | **Study design:** CBA **CCM elements:** SMS **Chronic condition:** Diabetes  **Country:** Sweden **Setting:** Community-based care **Duration of intervention in (months):** 4.0  **Number of patients:** 52.0 **Number of health professionals:** **Study Aim:** To assess the effects of regular walking on metabolic control and cardiovascular risk factors in type 2 diabetes | **Group 1 Intervention:**  Usual care  **Group 2 Intervention:** The patients in the intervention group were instructed to increase their exercise by 45 min of brisk walking, three times weekly, during 4 months | **Mean age group 1:** 60 (SD 7.3)  **Mean age group 2:** 59.3 (SD 6.2)  **% male group 1:** 57.7  **% male group 2:** 42.3  **Ethnicity group 1:** Not reported | **Primary outcome:**  HbA1c NS  **p =** |
| {Buist, 2006 #30} | **Study design:** RCT **CCM elements:** SMS **Chronic condition:** Asthma  **Country:** USA **Setting:** Managed care organisation **Duration of intervention in (months):** 24.0  **Number of patients:** 296.0 **Number of health professionals:** **Study Aim:** To determine whether peak flow monitoring has value above and beyond symptom monitoring when used as part of asthma management plan | **Group 1 Intervention:**  Use of symptoms for asthma monitoring  **Group 2 Intervention:** Use of peak flow rate for asthma monitoring | **Mean age group 1:** 66 (SD 9.199)  **Mean age group 2:** 66.0 (SD 9.6)  **% male group 1:** 48.0  **% male group 2:** 48.0    **Ethnicity group 1:** Non-Hispanic Caucasian | **Primary outcome:**  Health care utilization (HCU) NS  **p =** |
| {Houweling, 2011 #79} | **Study design:** RCT **CCM elements:** DSD+DS **Chronic condition:** Diabetes  **Country:** Netherlands **Setting:** Primary care **Duration of intervention in (months):** 14.0  **Number of patients:** 230.0 **Number of health professionals:** **Study Aim:** To determine whether the management of type 2 diabetes mellitus in a primary care setting can be safely transferred to practice nurses. | **Group 1 Intervention:**  Usual care  **Group 2 Intervention:** Practice nurses treated glucose levels, BP and lipid profile according to specified protocol. At the beginning of the intervention, the PNs received one week of training on a detailed treatment and management protocol aimed at optimising glucose, BP and lipid profile regulation and eye and foot care in patients with diabetes | **Mean age group 1:** 70 (SD 10.6)  **Mean age group 2:** 67.1 (SD 11)  **% male group 1:** 42.3  **% male group 2:** 52.9  **Ethnicity group 1:** Not reported | **Primary outcome:**  HbA1c NS (Statistical non-significance is a positive result in this study)  **p =** |
| {Smith, 2008 #147} | **Study design:** RCT **CCM elements:** DS+CIS **Chronic condition:** Diabetes  **Country:** USA **Setting:** Primary care **Duration of intervention in (months):** 21.0  **Number of patients:** 639.0 **Number of health professionals:** 97.0 **Study Aim:** To assess the effect of specialist telemedicine intervention for improving diabetes care using the chronic care model (CCM) | **Group 1 Intervention:**  Control physicians received e-mail with periodic generic information about cardiovascular risk reduction in diabetes  **Group 2 Intervention:** Specialist telemedicine intervention. Speciality advice and evidence -based messages regarding medication management for cardiovascular risk | **Median age group 1:** 60 (27-90)  **Median age group 2:** 62 (22-92)  **% male group 1:** 50.0  **% male group 2:** 45.0  **Ethnicity group 1:** Not reported | **Primary outcome:**  Diabetes care process (diabetes test completion) NS  **p =** |
| {Junghans, 2007 #83} | **Study design:** RCT **CCM elements:** DS+HCO **Chronic condition:** Heart disease  **Country:** UK **Setting:** Primary care **Duration of intervention in (months):**  **Number of patients: Number of health professionals:** 363.0 **Study Aim:** To determine the effect of patient-specific ratings vs conventional guidelines on appropriate investigation of angina. | **Group 1 Intervention:**  Conventional guidelines from the American Heart Association and the European Society of Cardiology  **Group 2 Intervention:** Patient-specific ratings (online prompt stating whether the specific vignette was considered appropriate of inappropriate for investigation, with access to detailed information on how rating was derived) | **Mean age group 1:** (SD )  **Mean age group 2:** (SD )  **% male group 1:**  **% male group 2:**  **Ethnicity group 1:** Not reported | **Primary outcome:**  Decisions for exercise electrocardiography were more appropriate with patient-specific ratings compared with conventioal guidelines SIG  **p =** 0.001 |
| {Efraimsson, 2008 #52} | **Study design:** RCT **CCM elements:** SMS+DSD **Chronic condition:** COPD  **Country:** Sweden **Setting:** Primary care **Duration of intervention in (months):** 4.0  **Number of patients:** 52.0 **Number of health professionals:** **Study Aim:** The aim was to examine the effects of a structured educational intervention programme at a nurse-led primary health care clinic (PHCC) on quality of life(QoL), knowledge about COPD and smoking cessation in patients with COPD. | **Group 1 Intervention:**  Usual care  **Group 2 Intervention:** Patients in the intervention group were offered two visits to a nurse specialised in COPD care. The purpose of these visits was to increase the patients' self-care and their knowledge about COPD. The educational visits were based on motivational dialogue tailored for each patient based on severity of illness, age, intellectual capacity and life style. When needed, a dietician, a medical social worker, a physical therapist and an occupational therapist were consulted | **Mean age group 1:** 67 (SD 10.4)  **Mean age group 2:** 66.0 (SD 9.4)  **% male group 1:** 50.0  **% male group 2:** 50.0  **Ethnicity group 1:** Not reported | **Primary outcome:**  Quality of life SIG  **p =** 0.000 |
| {van der Meer, 2009 #273} | **Study design:** RCT **CCM elements:** SMS **Chronic condition:** Asthma  **Country:** Netherlands **Setting:** Primary care **Duration of intervention in (months):** 12.0  **Number of patients:** 200.0 **Number of health professionals:** **Study Aim:** To evaluate the effectiveness of Internet-based asthma self-management. | **Group 1 Intervention:**  Education about core information on asthma, action of medications, and inhaler technique instructions to all patients  **Group 2 Intervention:** Self-management education consisted of both Web-based and face-to-face, group-based education In addition to weekly assessments, patients could optionally report daily symptoms and lung function and were able to contact the asthma nurse through the Web or by telephone. | **Mean age group 1:** 37 (SD )  **Mean age group 2:** 36.0 (SD )  **% male group 1:** 29.0  **% male group 2:** 32.0  **Ethnicity group 1:** Not reported | **Primary outcome:**  Asthma-related quaiity of life at 12 months SIG  **p =** 0.001 |
| {Adolfsson, 2007 #6} | **Study design:** RCT **CCM elements:** SMS **Chronic condition:** Diabetes  **Country:** Sweden **Setting:** Primary care **Duration of intervention in (months):** 12.0  **Number of patients:** 101.0 **Number of health professionals:** **Study Aim:** The aim of the study was to evaluate the impact of empowerment group education on type 2 diabetes patients' confidence in diabetes knowledge, self-efficacy, satisfaction with daily life, BMI and glycaemic control compared with the impact of routine diabetes care on the same factors at a 1-year follow-up. | **Group 1 Intervention:**  Usual care  **Group 2 Intervention:** Empwoerment educationin a group by GPs and diabetes specialist nurses | **Mean age group 1:** 64 (SD 9)  **Mean age group 2:** 62.4 (SD 8.9)  **% male group 1:** 61.0  **% male group 2:** 57.0  **Ethnicity group 1:** Not reported | **Primary outcome:**  Diabetes knowledge SIG  **p =** 0.049 |
| {Nielsen, 2006 #115} | **Study design:** RCT **CCM elements:** SMS+DS **Chronic condition:** Diabetes  **Country:** Denmark **Setting:** Primary care **Duration of intervention in (months):** 72.0  **Number of patients:** 484.0 **Number of health professionals:** **Study Aim:** To explore the relationship between HbA1c, sex, treatment allocation, and their interactions with behavioral and attitudinal characteristics in patients with type 2 diabetes. | **Group 1 Intervention:**  Usual care  **Group 2 Intervention:** Structured personal diabetes care, regular visits and goal setting for risk factors. GPs used guidelines | **Mean age group 1:** 63 (SD )  **Mean age group 2:** 63.7 (SD )  **% male group 1:** 48.8  **% male group 2:** 52.3    **Ethnicity group 1:** Not reported | **Primary outcome:**  HbA1c SIG  **p =** 0.010 |
| {D'Eramo Melkus, 2010 #291} | **Study design:** RCT **CCM elements:** SMS+CIS **Chronic condition:** Diabetes  **Country:** USA **Setting:** Primary care **Duration of intervention in (months):** 24.0  **Number of patients:** 109.0 **Number of health professionals:** **Study Aim:** An 11-week culturally relevant group diabetes self-management training (DSMT), coping skills training (CST), and diabetes care intervention was compared to a 10-week usual diabetes education and diabetes care intervention on physiological and psychosocial outcomes in 109 Black women (aged 48 +/- 10 years) with type 2 diabetes in primary care (PC). | **Group 1 Intervention:**  Education only  **Group 2 Intervention:** Culturally relevant diabetes self-management training and coping skills intervention | **Mean age group 1:** 45 (SD 10)  **Mean age group 2:** 47.0 (SD 9)  **% male group 1:** 0.0  **% male group 2:** 0.0  **Ethnicity group 1:** African American | **Primary outcome:**  HbA1c NS  **p =** |
| {Eaton, 2011 #50} | **Study design:** RCT **CCM elements:** DS+CIS+SMS **Chronic condition:** Lipid disorders  **Country:** USA **Setting:** Primary care **Duration of intervention in (months):** 12.0  **Number of patients: 4105 Number of health professionals:** 15 practices **Study Aim:** To determine whether an intervention based on patient activation and a physician decision support tool was more effective than usual care for improving adherence to National Cholesterol Education Program guidelines. | **Group 1 Intervention:**  Usual care  **Group 2 Intervention:** Academic detailing regarding cholesterol guidelines. Patient education toolkit, electronic decision support tool | **Mean age group 1:** (SD )  **Mean age group 2:** (SD )  **% male group 1:** 46.7  **% male group 2:** 46.4    **Ethnicity group 1:** Caucasian | **Primary outcome:**  Percentage screened for cholesterol NS  **p =** |
| {Marra, 2012 #103} | **Study design:** RCT **CCM elements:** DSD+SMS+DS **Chronic condition:** Arthritis  **Country:** Canada **Setting:** Community based care **Duration of intervention in (months):** 6.0  **Number of patients: Number of health professionals:** 139.0 **Study Aim:** In the PhIT-OA, we evaluated whether pharmacists could address the gaps in OA patient care as measured using quality of care indicators and health-related quality of life markers. | **Group 1 Intervention:**  Usual care  **Group 2 Intervention:** Knee exercises | **Mean age group 1:** 61 (SD 7.2)  **Mean age group 2:** 62.7 (SD 9.199)  **% male group 1:** 44.0  **% male group 2:** 42.0  **Ethnicity group 1:** Caucasian | **Primary outcome:**  Quality indicator pass rate SIG  **p =** 0.010 |
| {Billue, 2012 #21} | **Study design:** RCT **CCM elements:** DS **Chronic condition:** Diabetes  **Country:** USA **Setting:** Primary care **Duration of intervention in (months):** 24.0  **Number of patients: Number of health professionals:** 205.0 **Study Aim:** To determine the effectiveness of a provider-based intervention to improve medication intensification among patients with diabetes. | **Group 1 Intervention:**  Diabetes guidelines and patient education materials  **Group 2 Intervention:** Multicomponent interactive intervention including web-based continuing medical education (CME), performance feedback and quality improvement tools. **Group 3 Intervention: Group 4 Intervention:** | **Mean age group 1:** 61 (SD 13.79)  **Mean age group 2:** 58.7 (SD 13.59)  **% male group 1:** 50.5  **% male group 2:** 48.9  **Ethnicity group 1:** Not reported | **Primary outcome:**  Medication intensification NS  **p =** |
| {Persell, 2012 #269} | **Study design:** ITS **CCM elements:** CIS **Chronic condition:** Heart disease  **Country:** USA **Setting:** Primary care **Duration of intervention in (months):** 36.0  **Number of patients: Number of health professionals:** **Study Aim:** To evaluate the effects of a multifaceted quality improvement intervention during 2 time periods on 4 coronary artery disease [CAD] measures in 4 primary care practices. | **Group 1 Intervention:**  Multifaceted quality improvement intervention during 2 time periods on 4 coronary artery disease [CAD] measures. Electronic reminders prompted physicians to order indicated medications or record contraindications and refusals (exceptions). In the second phase, physicians also received reports about their performance (including lists of patients not satisfying these measures), and financial incentives were announced | **Mean age group 1:** 65 (SD )  **% male group 1:** 68.5  **Ethnicity group 1:** Caucasian | **Primary outcome:**  Antiplatelet medication for heart disease SIG  **p =** 0.010 |
| {Ladhani, 2012 #91} | **Study design:** RCT **CCM elements:** DSD **Chronic condition:** Heart disease  **Country:** Canada **Setting:** Primary care **Duration of intervention in (months):** 12.0  **Number of patients:** 195.0 **Number of health professionals:** **Study Aim:** To determine the impact of adding pharmacists to primary care teams on predicted 10-year risk of cardiovascular events in patients with Type 2 diabetes without established cardiovascular disease. | **Group 1 Intervention:**  Usual care  **Group 2 Intervention:** Pharmacists working with GP to manage diabetes | **Mean age group 1:** 57 (SD )  **Mean age group 2:** 56.5 (SD )  **% male group 1:** 57.8  **% male group 2:** 42.2    **Ethnicity group 1:** Not reported | **Primary outcome:**  UKPDS risk at 1 year SIG  **p =** 0.032 |
| {Turner, 2012 #164} | **Study design:** RCT **CCM elements:** SMS+DSD **Chronic condition:** Hypertension  **Country:** USA **Setting:** Primary care **Duration of intervention in (months):** 6.0  **Number of patients:** 280.0 **Number of health professionals:** **Study Aim:** We evaluated a peer and practice team intervention on reduction in 4-year coronary heart disease risk and systolic blood pressure. | **Group 1 Intervention:**  Usul care  **Group 2 Intervention:** 3-monthly calls from trained peer with well controlled BP, two practice visits to review CVD risk | **Mean age group 1:** 62 (SD 8.34)  **Mean age group 2:** 61.2 (SD 9.289)  **% male group 1:** 39.0  **% male group 2:** 30.0  **Ethnicity group 1:** African American | **Primary outcome:**  4 year coronary heart disease risk NS  **p =** |
| {Lin, 2012 #95} | **Study design:** RCT **CCM elements:** DSD+SMS **Chronic condition:** Diabetes  **Country:** USA **Setting:** Primary care **Duration of intervention in (months):** 24.0  **Number of patients:** 214.0 **Number of health professionals:** **Study Aim:** In a collaborative, team-based, care management program for complex patients (TEAMcare), we assessed patient and physician behaviors (medication adherence, self-monitoring, and treatment adjustment) in achieving better outcomes for diabetes, coronary heart disease, and depression. | **Group 1 Intervention:**  Usual care  **Group 2 Intervention:** In the TEAMcare program, a nurse care manager collaborated closely with primary care physicians, patients, and consultants to deliver a treat-to-target approach across multiple conditions. Measures included medication initiation, adjustment, adherence, and disease self-monitoring. | **Mean age group 1:** 57 (SD 11.3)  **Mean age group 2:** 56.8 (SD 11.3)  **% male group 1:** 47.6  **% male group 2:** 47.6  **Ethnicity group 1:** Not reported | **Primary outcome:**  Pharmacoinitiation antidepressants SIG  **p =** 0.010 |
| {Lowrie, 2011 #97} | **Study design:** RCT **CCM elements:** DS+SMS **Chronic condition:** Heart disease  **Country:** UK **Setting:** Primary care **Duration of intervention in (months):** 56.0  **Number of patients:** 2164.0 **Number of health professionals:** **Study Aim:** To test the hypothesis that a low-cost, low-intensity pharmacist intervention to optimize medical treatments, particularly ACE inhibitors, ARBs, and β-blockers, in patients identified in primary care with left ventricular systolic dysfunction would reduce the composite outcome of hospital admission for worsening heart failure or death, as well as other clinically important outcomes. | **Group 1 Intervention:**  Usual care  **Group 2 Intervention:** Pharmacists working with GP to optimsie medical treatemt for heart failure | **Mean age group 1:** 71 (SD 10.1)  **Mean age group 2:** 70.6 (SD 10.3)  **% male group 1:** 31.0  **% male group 2:** 29.0  **Ethnicity group 1:** Not reported | **Primary outcome:**  Composite death any cause or hospital admission for heart failure NS  **p =** |
| {Appel, 2011 #11} | **Study design:** RCT **CCM elements:** SMS+DSD+DS **Chronic condition:** Hypertension  **Country:** USA **Setting:** Community based care **Duration of intervention in (months):** 24.0  **Number of patients:** 415.0 **Number of health professionals:** **Study Aim:** We conducted a randomized, controlled trial to determine the effectiveness of two behavioral weight-loss interventions — including one without in-person contact — in obese patients with at least one cardiovascular risk factor. | **Group 1 Intervention:**  Usual care  **Group 2 Intervention:** Web based weight management **Group 3 Intervention:** In-person weight management | **Mean age group 1:** 53 (SD 10.1)  **Mean age group 2:** 55.8 (SD 9.699)  **Mean age group 3:** 53.3 (SD 10.5)  **% male group 1:** 36.2  **% male group 2:** 36.7  **% male group 3:** 36.2  **Ethnicity group 1:** Caucasian | **Primary outcome:**  Weight SIG  **p =** 0.010 |
| {Holbrook, 2011 #77} | **Study design:** RCT **CCM elements:** CIS+DS **Chronic condition:** Hypertension  **Country:** Canada **Setting:** Primary care **Duration of intervention in (months):** 12.0  **Number of patients:** 1102.0 **Number of health professionals:** **Study Aim:** We undertook a pragmatic randomized controlled trial of a CDSS-anchored intervention in primary care to assess its effects on relevant patient and physician outcomes. | **Group 1 Intervention:**  Usual care  **Group 2 Intervention:** Computerization of Medical Practices for the Enhancement of Therapeutic Effectiveness (COMPETE III) CDSS - Personally tailored electronic vascular risk monitoring and treatment advice shared between the physician and patient, risk calculation, and a clinical resource. | **Mean age group 1:** 69 (SD 8.800)  **Mean age group 2:** 69.3 (SD 8.6)  **% male group 1:** 46.5  **% male group 2:** 46.8  **Ethnicity group 1:** Not reported | **Primary outcome:**  Process outcomes score SIG  **p =** 0.010 |
| {Oei, 2011 #117} | **Study design:** **CCM elements:** DS **Chronic condition:**  **Country:** **Setting: Duration of intervention in (months):**  **Number of patients:** 219 **Number of health professionals:** **Study Aim:** Although guidelines for asthma emphasize the importance of spirometry for continuity and evaluation of care, it is underused in general practice. The objective of this study was to investigate the effect of spirometry and medical review on asthma control in general practice over 12 months. | **Group 1 Intervention:**  Usual care  **Group 2 Intervention:** 3 monthly spirometry and medical review **Group 3 Intervention:** Spirometry before and after the study | **Mean age group 1:** 48 (SD 17)  **Mean age group 2:** 46 (SD 17)  **Mean age group 3:** 48 (SD 19)  **% male group 1:** 28  **% male group 2:** 30  **% male group 3:** 29  **Ethnicity group 1:** Not reported | **Primary outcome:**  Asthma control 12 months NS  **p =** |
| {Katon, 2010 #85} | **Study design:** RCT **CCM elements:** DSD+SMS+DS **Chronic condition:** Diabetes  **Country:** USA **Setting:** Primary care **Duration of intervention in (months):** 12.0  **Number of patients:** 214.0 **Number of health professionals:** **Study Aim:** We conducted a study to determine whether coordinated care management of multiple conditions improves disease control in these patients. | **Group 1 Intervention:**  Usual care  **Group 2 Intervention:** Nurse to provide guideline based care for depression. GP supervised the nurse. The gola was to reduce risk factors | **Mean age group 1:** 56 (SD 12.1)  **Mean age group 2:** 57.4 (SD 10.5)  **% male group 1:** 44.0  **% male group 2:** 52.0  **Ethnicity group 1:** Caucasian | **Primary outcome:**  HbA1c SIG  **p =** 0.010 |
| {Evans, 2010 #56} | **Study design:** RCT **CCM elements:** SMS+DSD **Chronic condition:** Heart disease  **Country:** Canada **Setting:** Primary care **Duration of intervention in (months):** 6.0  **Number of patients:** 167.0 **Number of health professionals:** **Study Aim:** To evaluate whether a simple pharmacist protocol, consisting of patient screening and cardiovascular risk stratification, identification and reminders about uncontrolled risk factors, and drug adherence support, can significantly reduce cardiovascular risk. | **Group 1 Intervention:**  Pharmacists assessment and then usual care  **Group 2 Intervention:** Pharmacists provided ongoing counselling to patients | **Mean age group 1:** 60 (SD 10.19)  **Mean age group 2:** 60.3 (SD 10.05)  **% male group 1:** 78.4  **% male group 2:** 83.0  **Ethnicity group 1:** Not reported | **Primary outcome:**  Framingham risk score (% 10y risk) NS  **p =** |
| {Edelman, 2010 #51} | **Study design:** RCT **CCM elements:** DSD+SMS **Chronic condition:** Diabetes  **Country:** USA **Setting:** Primary care **Duration of intervention in (months):** 12.0  **Number of patients:** 239.0 **Number of health professionals:** **Study Aim:** To test the effectiveness of GMCs in the management of comorbid diabetes and hypertension. | **Group 1 Intervention:**  Usual care  **Group 2 Intervention:** Group medical clinics with 7-8 patients and care team. Group education and pharmacists and GP managed medications | **Mean age group 1:** 61 (SD 10)  **Mean age group 2:** 63.0 (SD 9.4)  **% male group 1:** 96.2  **% male group 2:** 95.5    **Ethnicity group 1:** African American | **Primary outcome:**  SBP SIG  **p =** 0.011 |
| {Hansson, 2010 #73} | **Study design:** RCT **CCM elements:** SMS+DSD **Chronic condition:** Osteoarthritis  **Country:** Sweden **Setting:** Primary care **Duration of intervention in (months):** 6.0  **Number of patients:** 114.0 **Number of health professionals:** **Study Aim:** The aim of this study was to evaluate the effects of this education programme for patients with OA in primary health care in terms of self-efficacy, function and self-perceived health. | **Group 1 Intervention:**  Usual care  **Group 2 Intervention:** Patient education programme for osteoarthritis (PEPOA), 5 group education sessions delivered by allied health, specialist and nurse | **Mean age group 1:** 63 (SD 9.51)  **Mean age group 2:** 62.0 (SD 9.43)  **% male group 1 & 2:** 14.9  **Ethnicity group 1:** Not reported | **Primary outcome:**  EQ5D regular (QOL) NS  **p =** |
| {Sperl-Hillen, 2010 #150} | **Study design:** RCT **CCM elements:** DS **Chronic condition:** Diabetes  **Country:** USA **Setting:** Primary care **Duration of intervention in (months):** 12.0  **Number of patients:** 3417.0 **Number of health professionals:** 40.0 **Study Aim:** Our objective was to assess the impact of an individualized simulated learning intervention on diabetes care provided by PCPs. | **Group 1 Intervention:**  Usual care  **Group 2 Intervention:** Simulated patient education for physicans with diabetes cases | **Mean age group 1:** 57 (SD 10.4)  **Mean age group 2:** 55.9 (SD 10.9)  **% male group 1:** 46.0  **% male group 2:** 55.4  **Ethnicity group 1:** Caucasian | **Primary outcome:**  HbA1c SIG  **p =** 0.034 |
| {Jameson, 2010 #205} | **Study design:** RCT **CCM elements:** DSD+SMS+DS **Chronic condition:** Diabetes  **Country:** USA **Setting:** Primary care **Duration of intervention in (months):** 12.0  **Number of patients:** 103.0 **Number of health professionals:** **Study Aim:** To investigate the effect of pharmacist management of poorly controlled diabetes mellitus in a community-based primary care group. | **Group 1 Intervention:**  Targeted patient outreach and registries  **Group 2 Intervention:** Targeted patient outreach and registries and medication management by the pharmacist, patient education | **Mean age group 1:** 50 (SD 10.9)  **Mean age group 2:** 49.3 (SD 10.8)  **% male group 1:** 49.0  **% male group 2:** 48.9  **Ethnicity group 1:** Caucasian | **Primary outcome:**  HbA1c NS  **p =** |
| {McManus, 2010 #107} | **Study design:** RCT **CCM elements:** SMS+DS **Chronic condition:** Hypertension  **Country:** UK **Setting:** Primary care **Duration of intervention in (months):** 12.0  **Number of patients:** 527.0 **Number of health professionals:** **Study Aim:** This study assessed whether self-management by people with poorly controlled hypertension resulted in better blood pressure control compared with usual care. | **Group 1 Intervention:**  Usual care  **Group 2 Intervention:** Patient self-management of hypertension (TASMINH2) with telehealth monitoring of BP, self-titration of medication. Traffic light system to guide treatment changes | **Mean age group 1:** 66 (SD 8.800)  **Mean age group 2:** 66.6 (SD 8.800)  **% male group 1:** 47.0  **% male group 2:** 47.0  **Ethnicity group 1:** Caucasian | **Primary outcome:**  SBP SIG  **p =** 0.010 |
| {Svetkey, 2009 #158} | **Study design:** RCT **CCM elements:** SMS+DS **Chronic condition:** Hypertension  **Country:** USA **Setting:** Primary care **Duration of intervention in (months):** 6.0  **Number of patients:** 574.0 **Number of health professionals:** 32.0 **Study Aim:** Hypertension improvement project: randomized trial of quality improvement for physicians and lifestyle modification for patients. | **Group 1 Intervention:**  Usual care (Patient and physician control)  **Group 2 Intervention: Group 3 Intervention:**  Patient intervention (20 weekly group sessions on lifestyle risk factor modification), Physician control  **Group 4 Intervention:**  Patient intervention (20 weekly group sessions on lifestyle risk factor modification), Physician intervention Online training, treatment algorithm, clinical performance measures | **Mean age group 1:** 60.5 (SD 11.4)  **Mean age group 2:** 61.6 (SD 12.2)  **Mean age group 3:** 59.0 (SD 12.3)  **Mean age group 4:** 60.7 (SD 11)  **% male group 1:** 53.9  **% male group 2:** 60.8  **% male group 3:** 52.9  **% male group 4: 62.1**  **Ethnicity group 1:** Caucasian 93% | **Primary outcome:**  BP SIG  **p =** 0.010 |
| {Murphy, 2009 #113} | **Study design:** RCT **CCM elements:** DS+SMS **Chronic condition:** Heart disease  **Country:** UK **Setting:** Primary care **Duration of intervention in (months):** 18.0  **Number of patients:** 903.0 **Number of health professionals:** **Study Aim:** To test the effectiveness of a complex intervention designed, within a theoretical framework, to improve outcomes for patients with coronary heart disease. | **Group 1 Intervention:**  Usual care  **Group 2 Intervention:** Tailored care plans for practices (practice based training in prescribing and behaviour change, administrative support, quarterly newsletter), and tailored care plans for patients (motivational interviewing, goal identification, and target setting for lifestyle change) with reviews every four months at the practices. | **Mean age group 1:** 67 (SD 9.9)  **Mean age group 2:** 68.5 (SD 9.300)  **% male group 1:** 70.0  **% male group 2:** 70.0  **Ethnicity group 1:** Not reported | **Primary outcome:**  SBP NS  **p =** |
| {Soran, 2008 #148} | **Study design:** RCT **CCM elements:** SMS+CIS **Chronic condition:** Heart disease  **Country:** USA **Setting:** Primary care **Duration of intervention in (months):** 6.0  **Number of patients:** 315.0 **Number of health professionals:** **Study Aim:** The present study was designed to assess the impact of a computer-based home disease management program (Alere DayLink HF Monitoring System [HFMS]) on the clinical outcomes of Medicare beneficiaries with HF who were elderly, women, and non-white males who received the care from a community-based primary care practitioner. | **Group 1 Intervention:**  Usual care  **Group 2 Intervention:** HFMS consists of a home-based disease management program to monitor and to detect early signs and symptoms of heart failure using telecommunication equipment; such early detection could allow practitioners to focus their clinical resources on patients needing interventions. | **Mean age group 1:** 76 (SD 6.8)  **Mean age group 2:** 76.9 (SD 7.1)  **% male group 1:** 39.4  **% male group 2:** 31.2  **Ethnicity group 1:** African American 52.3% | **Primary outcome:**  Treatment failure (CV death or HF hospitalisation) NS  **p =** |
| {Cleveringa, 2008 #35} | **Study design:** RCT **CCM elements:** DSD+DS+CIS **Chronic condition:** Diabetes  **Country:** Netherlands **Setting:** Primary care **Duration of intervention in (months):** 12.0  **Number of patients:** 3391.0 **Number of health professionals:** **Study Aim:** We studied the effect of the Diabetes Care Protocol on A1C and cardiovascular risk factors in type 2 diabetic patients in primary care. | **Group 1 Intervention:**  Usual care  **Group 2 Intervention:** The Diabetes Care Protocol combines task delegation (a practice nurse), computerized decision support, and feedback every 3 months. | **Mean age group 1:** 65 (SD 11)  **Mean age group 2:** 65.2 (SD 11.3)  **% male group 1:** 49.8  **% male group 2:** 48.2  **Ethnicity group 1:** Caucasian | **Primary outcome:**  HbA1c NS  **p =** |
| {Peterson, 2008 #126} | **Study design:** RCT **CCM elements:** CIS+DS **Chronic condition:** Diabetes  **Country:** USA **Setting:** Primary care **Duration of intervention in (months):** 12.0  **Number of patients:** 8405 **Number of health professionals:** 238 **Study Aim:** The purpose of this study was to determine whether implementation of a multicomponent organizational intervention can produce significant change in diabetes care and outcomes in community primary care practices. | **Group 1 Intervention:**  Usual care  **Group 2 Intervention:** TRANSLATE intervention included implementation of an electronic diabetes registry, visit reminders, and patient-specific physician alerts. A site coordinator facilitated previsit planning and a monthly review of performance with a local physician champion | **Mean age group 1:** 63 (SD 0.92)  **Mean age group 2:** 62.4 (SD 0.91)  **% male group 1:** 49.5  **% male group 2:** 51.0    **Ethnicity group 1:** Not reported | **Primary outcome:**  Composite outcome (SBP, LDL, HbA1c) SIG  **p =** 0.010 |
| {van Bruggen, 2008 #167} | **Study design:** RCT **CCM elements:** DS+DSD **Chronic condition:** Diabetes  **Country:** Netherlands **Setting:** Primary care **Duration of intervention in (months):** 12.0  **Number of patients:** 1640.0 **Number of health professionals:** **Study Aim:** To assess the effects of a facilitator enhanced multifaceted intervention to implement a locally adapted guideline on the shared care for people with type 2 diabetes. | **Group 1 Intervention:**  Usual care  **Group 2 Intervention:** Nurse facilitators enhanced guideline implementation by analysing barriers to change, introducing structured care, training practice staff and giving performance feedback. Targets for HbA1c%, systolic blood pressure as well as indications for angiotensin converting enzyme/angiotensin receptor blocking agent prescription differed from the national guidelines. | **Mean age group 1:** 67 (SD 11.9)  **Mean age group 2:** 67.1 (SD 11.4)  **% male group 1:** 50.4  **% male group 2:** 46.8  **Ethnicity group 1:** Not reported | **Primary outcome:**  HbA1c NS  **p =** |
| {Hörnsten, 2008 #78} | **Study design:** RCT **CCM elements:** SMS+DS **Chronic condition:** Diabetes  **Country:** Sweden **Setting:** Primary care **Duration of intervention in (months):** 60.0  **Number of patients:** 102.0 **Number of health professionals:** **Study Aim:** This paper reports a 5-year follow-up from a study aimed at evaluating whether an intervention which focused on patients' personal understanding of their illness was more effective than conventional diabetes care with regard to metabolic control among patients with type 2 diabetes mellitus (DM2). | **Group 1 Intervention:**  Usual care  **Group 2 Intervention:** Patient education, 2-hour group sessions for 9 months | **Mean age group 1:** 63 (SD 9.1)  **Mean age group 2:** 63.6 (SD 9.300)  **% male group 1:** 55.0  **% male group 2:** 52.0  **Ethnicity group 1:** Not reported | **Primary outcome:**  HbA1c SIG  **p =** 0.010 |
| {Majumdar, 2007 #101} | **Study design:** RCT **CCM elements:** DS **Chronic condition:** Heart disease  **Country:** Canada **Setting:** Primary care **Duration of intervention in (months):** 6.0  **Number of patients:** 171.0 **Number of health professionals:** **Study Aim:** We hypothesized that an intervention consisting of patient-specific one-page evidence summaries, generated and endorsed by local opinion leaders, would improve prescribing of angiotensin-converting enzyme (ACE) inhibitors or angiotensin receptor blockers (ARBs) in heart failure (HF) and that of statins in ischemic heart disease (IHD). | **Group 1 Intervention:**  Only medication profile faxed  **Group 2 Intervention:** One page evidence summary faxed to GP and medication profile | **Median age group 1:** 74 (Range 66-80)  **Median age group 2:** 76 (Range 69-82)  **% male group 1:** 45.0  **% male group 2:** 34.0  **Ethnicity group 1:** Not reported | **Primary outcome:**  Percentage effective meds prescribed NS  **p =** |
| {Scott, 2006 #136} | **Study design:** RCT **CCM elements:** DSD+SMS **Chronic condition:** Diabetes  **Country:** USA **Setting:** Community based care **Duration of intervention in (months):** 9.0  **Number of patients:** 149.0 **Number of health professionals:** **Study Aim:** The outcomes of pharmacist-managed diabetes care services in a community health center were studied. | **Group 1 Intervention:**  Usual care  **Group 2 Intervention:** Pharmacist managed diabetes care program | **Age group 1:** 54.8% >=50  **Age group 2:** 50% >= 50  **% male group 1:** 35.6  **% male group 2:** 42.1  **Ethnicity group 1:** Caucasian | **Primary outcome:**  HbA1c SIG  **p =** 0.049 |
| {Richardson, 2010 #130} | **Study design:** RCT **CCM elements:** DSD+SMS+DS **Chronic condition:** Hypertension  **Country:** Canada **Setting:** Primary care **Duration of intervention in (months):** 9.0  **Number of patients:** 303.0 **Number of health professionals:** **Study Aim:** The primary objective of this study was to determine whether adults with a chronic illness within a primary care setting who received a rehabilitation intervention in this setting showed greater improvement in health status and had fewer hospital admissions and emergency room visits compared with adults who do not receive the intervention. Background More than half of Canadians (16 million people) | **Group 1 Intervention:**  Usual care  **Group 2 Intervention:** A rehabilitation multi-component intervention was delivered by a physiotherapist (PT) and occupational therapist in a primary care setting and included collaborative goal setting for rehabilitation needs, a six-week chronic disease self-management (SM) workshop, referral to community programs and a web-based education programme | **Mean age group 1:** (SD )  **Mean age group 2:** (SD )  **% male group 1:** 35.5  **% male group 2:** 37.7  **Ethnicity group 1:** Not reported | **Primary outcome:**  Improvement in health status (via SF-36 questionnaire) NS  **p =** |
| {Glasgow, 2010 #64} | **Study design:** RCT **CCM elements:** SMS **Chronic condition:** Diabetes  **Country:** USA **Setting:** Community based care **Duration of intervention in (months):** 4.0  **Number of patients:** 463.0 **Number of health professionals:** **Study Aim:** This study evaluated minimal and moderate support versions of an Internet-based diabetes self-management program, compared to an enhanced usual care condition. | **Group 1 Intervention:**  Usual care  **Group 2 Intervention:** CASM - website with graphical display of patients HbAic, BP and cholesterol. Self-managem education, goal setting and action plans **Group 3 Intervention:** CASM plus follow up telephone calls from health professionals | **Mean age group 1:** 59 (SD 9.1)  **Mean age group 2:** 58.7 (SD 9.300)  **Mean age group 3:** 57.8 (SD 9.3)  **% male group 1:** 48.5  **% male group 2:** 55.4  **% male group 3:** 46.3  **Ethnicity group 1:** Caucasian | **Primary outcome:**  Behaviour modification SIG  **p =** 0.010 |
| {Lin, 2006 #277} | **Study design:** RCT **CCM elements:** SMS+DSD **Chronic condition:** Diabetes  **Country:** USA **Setting:** Primary care **Duration of intervention in (months):** 12.0  **Number of patients:** 329.0 **Number of health professionals:** **Study Aim:** Among patients with diabetes, major depression is associated with more diabetic complications, lower medication adherence, and poorer self-care of diabetes. We reported earlier that enhanced depression care reduces depression symptoms but not hemoglobin A1c level. This study examined effects of depression interventions on self-management among depressed diabetic patients. | **Group 1 Intervention:**  Usual care  **Group 2 Intervention:** Nurse management of depression. Individualised management which inlcude medication or problme solving skills. Lifestyle counselling | **Mean age group 1:** 58 (SD )  **Mean age group 2:** 58.6 (SD )  **% male group 1:** 35.2  **% male group 2:** 34.8  **Ethnicity group 1:** Caucasian | **Primary outcome:**  Self care NS  **p =** |
| {Agvall, 2013 #7} | **Study design:** RCT **CCM elements:** SMS+DSD **Chronic condition:** Heart failure  **Country:** Sweden **Setting:** Primary care **Duration of intervention in (months):** 12.0  **Number of patients:** 160.0 **Number of health professionals:** **Study Aim:** The purpose of this study was to evaluate if the use of HF management programmes (HFMPs) also has beneficial effects on HF patients in primary health-care (PHC) | **Group 1 Intervention:**  Usual care  **Group 2 Intervention:** Participants were subject to a follow-up examination performed by the HF nurses with the support of a GP with the aim of optimizing treatment according to guidelines. All participants received oral and written information about HF. All participants in the intervention group had the possibility of directly contacting the HF nurseat the PHCC by telephone during office hours | **Mean age group 1:** 75 (SD 7.1)  **Mean age group 2:** 75.0 (SD 8.6)  **% male group 1:** 64.0  **% male group 2:** 73.0  **Ethnicity group 1:** Not reported | **Primary outcome:**  The primary outcome was a composite endpoint based on changes in EF, NT-proBNP levels and Qol assessed using the well-validated Short-Form 36 (Sf-36) questionnaire, and all mortality SIG  **p =** 0.010 |
| {Bardach, 2013 #15} | **Study design:** RCT **CCM elements:** HCO+CIS **Chronic condition:** Heart disease  **Country:** USA **Setting:** Primary care **Duration of intervention in (months):** 11.0  **Number of patients:** 84.0 **Number of health professionals:** **Study Aim:** To assess the effect of P4P incentives on quality in EHR-enabled small practices in the context of an established quality improvement initiative | **Group 1 Intervention:**  Usual care  **Group 2 Intervention:** Incentivized clinics were paid for each patient whose care met the performance criteria, but they received higher payments for patients with comorbidities Intervention group receiving financial incentives and benchmarked quarterly reports of their performance | **Mean age group 1:** 47 (SD 4.8)  **Mean age group 2:** 45.8 (SD 6.7)  **% male group 1:** 10.5  **% male group 2:** 8.6  **Ethnicity group 1:** Not reported | **Primary outcome:**  Rates of appropriate antithrombotic therapy SIG  **p =** 0.001 |
| {Beune, 2014 #20} | **Study design:** RCT **CCM elements:** SMS+CR **Chronic condition:** Hypertension  **Country:** Netherlands **Setting:** Primary care **Duration of intervention in (months):** 6.0  **Number of patients:** 146.0 **Number of health professionals:** **Study Aim:** To evaluate the effect of a practice-based, culturally appropriate patient education intervention on blood pressure (BP) and treatment adherence among patients of African origin with uncontrolled hypertension | **Group 1 Intervention:**  Usual care  **Group 2 Intervention:** Three nurse-led, culturally appropriate hypertension education sessions. Three structured 30-minute culturally appropriate couselling sessions at 2 weeks, 8 weeks and at 20 weeks after baseline assessment. Culturally appropriate writtten educational materials If applicable, referrals to neighbourhood facilities, such as walking clubs and health food stores, that support patients in adopting healthier lifestyles and are suitable for Surinamere and Ghanian people | **Mean age group 1:** 55 (SD 9.5)  **Mean age group 2:** 53.3 (SD 10.19)  **% male group 1:** 56.0  **% male group 2:** 39.0  **Ethnicity group 1:** Non-Caucasian | **Primary outcome:**  SBP NS  **p =** |
| {Black, 2013 #22} | **Study design:** RCT **CCM elements:** DSD+DS+HCO **Chronic condition:** Diabetes  **Country:** Australia **Setting:** Primary care **Duration of intervention in (months):** 12.0  **Number of patients:** 1853.0 **Number of health professionals:** **Study Aim:** This study aimed to evaluate the impact of a structured intervention involving non-GP staff in GP practices on the quality of care for patients with diabetes or cardiovascular disease | **Group 1 Intervention:**  Usual care  **Group 2 Intervention:** The intervention aimed to assist non-GP staff to work as a team when implementing practice systems that support chronic disease care. Non-GP staff included administrative staff and clinical staff but did not include allied health professionals. Initially, facilitators from the research team conducted an education session of 1-2 h with GP practice staff. Three 1-h practice visits were provided with resource manuals and workbooks for each of the 11 elements | **Mean age group 1:** 63 (SD 11.5)  **Mean age group 2:** 63.3 (SD 10.7)  **% male group 1:** 46.7  **% male group 2:** 46.4  **Ethnicity group 1:** Not reported | **Primary outcome:**  Patient-assessed quality of care NS  **p =** |
| {Blackberry, 2013 #23} | **Study design:** RCT **CCM elements:** SMS+DSD+DS **Chronic condition:** Diabetes  **Country:** Australia **Setting:** Primary care **Duration of intervention in (months):** 18.0  **Number of patients:** 473.0 **Number of health professionals:** **Study Aim:** To evaluate the effectiveness of goal focused telephone coaching by practice nurses in improving glycaemic control in patients with type2 diabetes in Australia | **Group 1 Intervention:**  Usual care  **Group 2 Intervention:** The intervention involved practice nurses being taught to deliver structured telephone coaching to prime patients with the aim of self managing their diabetes. Each practice nurse assigned to the intervention group received a two day training programme in telephone coaching | **Mean age group 1:** 62 (SD 10.5)  **Mean age group 2:** 63.6 (SD 10.4)  **% male group 1:** 60.0  **% male group 2:** 54.0  **Ethnicity group 1:** Not reported | **Primary outcome:**  HbA1c NS  **p =** |
| {Bove, 2013 #29} | **Study design:** RCT **CCM elements:** DSD+CIS+SMS **Chronic condition:** Hypertension  **Country:** USA **Setting:** Community-based care **Duration of intervention in (months):** 6.0  **Number of patients:** 241.0 **Number of health professionals:** **Study Aim:** We evaluated an Internet- and telephone-based telemedicine system for reducing blood pressure (BP) in underserved subjects with hypertension. | **Group 1 Intervention:**  Control subjects were provided with data from their initial assessment and instructed to contact their primary care provider for further care  **Group 2 Intervention:** The system is a Health Insurance Probability and Accountability Act compliant, secure, encrypted Web system that allows bidirectional data transfer between patient and practice. Telemedicine arm were given a sphygmomanometer, a scale if needed and a pedometer and instructions to their use. Data, text and voice messages are recorded in a database that provides patients with their information in the form of a personal health record | **Mean age group 1:** 58 (SD 13.5)  **Mean age group 2:** 61.0 (SD 13.6)  **% male group 1:** 35.0  **% male group 2:** 35.0  **Ethnicity group 1:** African American | **Primary outcome:**  BP NS  **p =** |
| {Chen, 2013 #33} | **Study design:** RCT **CCM elements:** DSD+DS+SMS **Chronic condition:** Hypertension  **Country:** USA **Setting:** Primary care **Duration of intervention in (months):** 6.0  **Number of patients:** 374.0 **Number of health professionals:** **Study Aim:** The objectives of this study were to compare indices of 24-hour blood pressure (BP) following a physician-pharmacist collaborative intervention and to describe the associated changes in antihypertensive medications | **Group 1 Intervention:**  Patients in both groups were given written information about managing BP  **Group 2 Intervention:** Pharmacists identified problems leading to poor BP control, created a care plan, and made specific recommendations to the patient's physician regarding changed in drug therapy For patients in the co-managed group, clinical pharmacists within the intervention offices evaluated medications and BP at baseline and 1 month, and by telephone at 3 months, with the option of more frequent contact if BP remained poorly controlled | **Mean age group 1:** 59 (SD 14)  **Mean age group 2:** 57.2 (SD 14.5)  **% male group 1:** 45.0  **% male group 2:** 38.6  **Ethnicity group 1:** White | **Primary outcome:**  SBP SIG  **p =** |
| {De San Miguel, 2013 #40} | **Study design:** RCT **CCM elements:** DSD+SMS+CIS **Chronic condition:** COPD  **Country:** Australia **Setting:** Community-based care **Duration of intervention in (months):** 6.0  **Number of patients:** 80.0 **Number of health professionals:** **Study Aim:** To determine if self-monitoring via home-based telehealth equipment could, when combined with ongoing remote monitoring by a nurse, reduce the incidence of hospitalizations and emergency department (ED) presentations for people with chronic obstructive pulmonary disease (COPD). | **Group 1 Intervention:**  COPD Book  **Group 2 Intervention:** Participants measured their vital signs and answered questions relating to their general state of health, on a daily basis. These were transmitted automatically via telephone to a secure Web site where they were monitored by the telehealth nurse Participants were visited at home by the telehealth nurse, who installed the telehealth equipement and trained participants in its use | **Mean age group 1:** 74 (SD )  **Mean age group 2:** 71.0 (SD )  **% male group 1:** 57.1  **% male group 2:** 38.9  **Ethnicity group 1:** Not reported | **Primary outcome:**  Hospital admissions NS  **p =** |
| {Gilani, 2013 #63} | **Study design:** **CCM elements:** DSD DS **Chronic condition:** Diabetes  **Country:** Canada **Setting:** Primary Care **Duration of intervention in (months):** 12  **Number of patients: Number of health professionals:** **Study Aim:** To assess the effect of adding pharmacists to primary care teams on initiation of guideline-concordant antiplatelet therapy in type 2 diabetic patient | **Group 1 Intervention:**  Usual care  **Group 2 Intervention:** Pharmacist intervention included a complete medication history, limited physical examination, provision of guideline-concordant recommendations to the physician to optimize drug therapy and 1-year follow-up Pharmacists maintained close follow-up with intervention patients for 1 year | **Mean age group 1:** 59.1 (SD 12.1)  **Mean age group 2:** 56.9 (SD 11.6)  **% male group 1:** 41  **% male group 2:** 39  **Ethnicity group 1:** Not reported | **Primary outcome:**  The primary outcome was the proportion of patients using antiplatelet drug at 1 year SIG  **p =** 0.001 |
| {Østerås, 2014 #120} | **Study design:** RCT **CCM elements:** DSD+SMS **Chronic condition:** Arthritis  **Country:** Norway **Setting:** Primary care **Duration of intervention in (months):** 6.0  **Number of patients:** 130.0 **Number of health professionals:** **Study Aim:** To determine the clinical effectiveness of an exercise programme on self-reported hand activity performance in people with hand osteoarthritis (OA) | **Group 1 Intervention:**  Usual care  **Group 2 Intervention:** All exercises were practiced with an occupational therapist during the first group session, which took place immediately post-randomization. In the 8 weeks with no group session, a weekly telephone call was made by one of the co-authors. The main purpose of the telephone call was to ensure adherence to the exercise programme as evidence of such effects has been reported previously | **Mean age group 1:** 65 (SD 9)  **Mean age group 2:** 67.0 (SD 8)  **% male group 1:** 9.0  **% male group 2:** 11.0  **Ethnicity group 1:** Not reported | **Primary outcome:**  Self-reported hand activity performance at 3 months measured by the Functional Index for Hand Osteoarthritis NS  **p =** |
| {Palmas, 2014 #121} | **Study design:** RCT **CCM elements:** SMS+CR+DSD **Chronic condition:** Diabetes  **Country:** USA **Setting:** Community-based care **Duration of intervention in (months):** 12.0  **Number of patients:** 360.0 **Number of health professionals:** **Study Aim:** The Northern Manhattan Diabetes Community Outreach Project evaluated whether a community health worker (CHW) intervention improved clinically relevant markers of diabetes care in adult Hispanics | **Group 1 Intervention:**  Four sets of Spanish-language educational materials containing information on communication between physician and patient, diabetes management, mental health, and a diabetes cookbook  **Group 2 Intervention:** Two full-time CHWs delivered a multicomponent intervention that included one-to-one visits, group visits, and telephone follow-up the focus of one-to one visits was to assess existing barriers to healthcare, empowering the patient and developing achievable goals. The focus of the group visits was mainly on nutrition education and exercise activities. | **Mean age group 1:** 58 (SD 7.8)  **Mean age group 2:** 57.1 (SD 7.7)  **% male group 1:** 37.4  **% male group 2:** 39.2  **Ethnicity group 1:** Hispanic/Latino | **Primary outcome:**  HbA1c NS  **p =** |
| {Pecina, 2013 #123} | **Study design:** RCT **CCM elements:** DSD+CIS **Chronic condition:** COPD  **Country:** USA **Setting:** Community-based care **Duration of intervention in (months):** 12.0  **Number of patients:** 205.0 **Number of health professionals:** **Study Aim:** Telemonitoring is being increasingly used for chronic disease monitoring. While the primary aim of telemonitoring is to improve chronic disease managementand decrease hospitalizations, the potential impact on patient’s health-related quality of life may be an additional benefit. | **Group 1 Intervention:** Daily home telemonitoring. Monitoring was done with the Intel Health Guide, which collects biometric data and administers symptom questionnaires with the goal of early detection of decline in health status. Any concerning results were addressed either by the nurse, a videoconference visit with a geriatric nurse practitioner, a message to or visit with the patients primary care provider, or referral to ED as appropriate **Group 2 Intervention:**  Usual care | **Mean age group 1:** 79.6 (SD 8.7)  **Mean age group 2:** 79.2 (SD 7.4)  **% male group 1:** 50.7  **% male group 2:** 43.8  **Ethnicity group 1:** Not reported | **Primary outcome:**  Physical component summary (PCS) on the Short Form Health Questionnaire (SF-12va) at 12 months NS  **p =** |
| {Pinnock, 2013 #127} | **Study design:** RCT **CCM elements:** DSD+CIS+SMS **Chronic condition:** COPD  **Country:** UK **Setting:** Primary care **Duration of intervention in (months):** 12.0  **Number of patients:** 256.0 **Number of health professionals:** **Study Aim:** To test the effectiveness of telemonitoring integrated into existing clinical services such that intervention and control groups have access to the same clinical care | **Group 1 Intervention:**  Conventional self-monitoring  **Group 2 Intervention:** Using a touch screen, telemonitoring participants recorded a daily questionnaire about symptoms and treatment use, and monitored oxygen saturation using linked instruments. The clinical team responsible for their care visited participant at home to explain how to use the technology and provide self management education | **Mean age group 1:** 68 (SD 8.4)  **Mean age group 2:** 69.4 (SD 8.800)  **% male group 1:** 49.0  **% male group 2:** 41.0  **Ethnicity group 1:** Not reported | **Primary outcome:**  The primary outcome was time to hospital admission due to COPD up to one year after randomisation NS  **p =** |
| {Quinn, 2013 #129} | **Study design:** RCT **CCM elements:** SMS+DS+CIS **Chronic condition:** Diabetes  **Country:** USA **Setting:** Primary care **Duration of intervention in (months):** 12.0  **Number of patients:** 163.0 **Number of health professionals:** **Study Aim:** To test whether adding mobile application coaching and patient/provider web portals to community primary care compared with standard diabetes management would reduce glycated hemoglobin levels in patients with type 2 diabetes | **Group 1 Intervention:**  Usual care  **Group 2 Intervention:** The patient coaching system included a mobile diabetes management software application and a web portal The mobile software application allowed patients to enter diabetes self-care data on a mobile phone and receive automated, real-time educational, behavioral and motivational messaging specific to the entered data The patient web portal augmented the mobile software application and consisted of a secure messaging center (Pt/prov comm) personal health record with additional diabetes information, learning library and logbook to review historical data **Group 3 Intervention:** The provider portal had different views for data on the basis of study group. The data-only view (group3, CPP) allowed access unanalyzed patient data. **Group 4 Intervention:** The provider portal had different views for data on the basis of study group. Group 4 (CPDS) providers had access to analyzed patient data linked to standards of care and evidence-based guidelines Group 4 (CPDS) providers had access to analyzed patient data linked to standards of care and evidence-based guidelines Coach-PCP Portal with Decision Support (CPDS) | **Mean age group 1:** 53 (SD 8.4)  **Mean age group 2:** 52.8 (SD 8)  **Mean age group 3:** 53.7 (SD 8.2)  **Mean age group 4:** 52.0 (SD 8.0)  **% male group 1:** 50.0  **% male group 2:** 52.2  **% male group 3:** 45.5  **% male group 4:** 50.0  **Ethnicity group 1:** African American | **Primary outcome:**  HbA1c SIG  **p =** 0.001 |
| {Rothschild, 2014 #134} | **Study design:** RCT **CCM elements:** SMS+DSD **Chronic condition:** Diabetes  **Country:** USA **Setting:** Community-based care **Duration of intervention in (months):** 24.0  **Number of patients:** 144.0 **Number of health professionals:** **Study Aim:** We assessed whether community health workers (CHWs) could improve glycemic control among Mexican Americans with diabetes. | **Group 1 Intervention:**  Bilingual newsletter called DiabetesAction  **Group 2 Intervention:** CHW delivered behavioral self-management training during 36 home visits over 2 years Prior to starting the intervention, 10 CHWs received more than 100 hours of training on diabetes, behavioral self-management support and home visiting. From those who demonstrated acquisition of the needed knowledge and skills, 3 were hired for MATCH | **Mean age group 1:** 54 (SD 12.7)  **Mean age group 2:** 53.7 (SD 11.7)  **% male group 1:** 29.6  **% male group 2:** 35.6  **Ethnicity group 1:** Hispanic/Latino | **Primary outcome:**  HbA1c SIG  **p =** 0.005 |
| {Sen, 2014 #137} | **Study design:** RCT **CCM elements:** HCO+DSD **Chronic condition:** Diabetes  **Country:** USA **Setting:** Community-based care **Duration of intervention in (months):** 3.0  **Number of patients:** 75.0 **Number of health professionals:** **Study Aim:** To test the effectiveness of two different magnitudes of financial incentives for improving adherence to remote-monitoring regimens among patients with poorly controlled diabetes. | **Group 1 Intervention:**  Twelve weeks of daily home-monitoring of blood glucose, blood pressure, and weight (Using biometric devices)  **Group 2 Intervention:** A lottery incentive with expected daily value of $1.40 for daily monitoring (using biometric devices) **Group 3 Intervention:** A lottery incentive with expected daily value of $2.80 for daily monitoring (Using biometric devices) **Group 4 Intervention:** | **Mean age group 1:** 54 (SD )  **Mean age group 2:** 54.7 (SD )  **Mean age group 3:** 54.3 (SD )  **% male group 1:** 39.0  **% male group 2:** 33.0  **% male group 3:** 35.0  **Ethnicity group 1:** African American | **Primary outcome:**  Daily use of three home-monitoring devices during the three-month intervention SIG  **p =** 0.007 |
| {Bellary, 2008 #17} | **Study design:** RCT **CCM elements:** DSD+SMS+DS **Chronic condition:** Diabetes  **Country:** UK **Setting:** Mixed **Duration of intervention in (months):** 24.0  **Number of patients:** 1486.0 **Number of health professionals:** **Study Aim:** We investigated the effectiveness of a culturally sensitive, enhanced care package in UK general practices for improvement of cardiovascular risk factors in patients of south Asian origin with type 2 diabetes. | **Group 1 Intervention:**  Usual care  **Group 2 Intervention:** Enhanced care included an additional practice nurse time (4 h practice per week), supported by link workers and a community nurse specialising in diabetes. All patients were contacted by a link worker before and between appointments to encourage clinic attendance. Additionally, link workers provided interpretation and additional educational input in local languages to patients. The two community diabetes-specialist nurses covered the nine intervention practices, and attended some research clinics every 6-8 weeks, providing additional educational and clinical support including insulin initiation to practice teams. Practices were encouraged to adhere to treatment protocols and to achieve targets | **Mean age group 1:** 57 (SD 11.9)  **Mean age group 2:** 57.0 (SD 11.9)  **% male group 1:** 49.0  **% male group 2:** 54.0  **Ethnicity group 1:** Non-Caucasian | **Primary outcome:**  BP SIG  **p =** 0.018 |
| {Fisher, 2013 #57} | **Study design:** RCT **CCM elements:** SMS+DSD **Chronic condition:** Diabetes  **Country:** USA **Setting:** Community-based care **Duration of intervention in (months):** 12.0  **Number of patients:** 392.0 **Number of health professionals:** **Study Aim:** To compare three interventions to reduce diabetes distress (DD) and improve self-management among non-clinically depressed adults with type 2 diabetes mellitus (T2DM). | **Group 1 Intervention:**  Patients randomized to Leap Ahead, a minimal intervention in comparison with the other two conditions, received a 20-min, computer-delivered health risk appraisal along with diabetes information preeceding each of the eight phone calls  **Group 2 Intervention:** Patients randomized to CASM were introduced to "My Path To A Healthy Life", a 40-minutes previously validated, web-based diabetes self-management improvement program The predominantly web-based intervention also provided an ask-the-expertforum to enhance engagement Patients received four live phone calls from their interventionist to check progress and problems regarding their use of CASM and to provide encouragement to continue their efforts **Group 3 Intervention:** Patients randomized to CAPS received a 60-min in-peron intervention that included CASM plus PST CASM (the intervention in group 2) As in CASM, CAPS patients received four live phone calls to check progress on CASM and PST, respond to problems, and provide encouragement and a live supplimental booster session at month 5 **.** | **Mean age group 1:** 55 (SD 10.8)  **Mean age group 2:** 56.9 (SD 8.699)  **Mean age group 3:** 55.8 (SD 9.3)  **% male group 1:** 40.6  **% male group 2:** 52.0  **% male group 3:** 43.8  **Ethnicity group 1:** Caucasian | **Primary outcome:**  Diabetes Distress Scale (DDS) NS  **p =** |
| {Davis, 2010 #39} | **Study design:** RCT **CCM elements:** SMS+DSD **Chronic condition:** Diabetes  **Country:** USA **Setting:** Mixed **Duration of intervention in (months):** 12.0  **Number of patients:** 165.0 **Number of health professionals:** **Study Aim:** To conduct a 1-year randomized clinical trial to evaluate a remote comprehensive diabetes self-management education (DSME) intervention, Diabetes TeleCare, administered by a dietitian and nurse/certified diabetes educator (CDE) in the setting of a federally qualified health center (FQHC) in rural South Carolina. | **Group 1 Intervention:**  Usual care consisted of one 20-minutes diabetes education session, usingt ADA materials, conducted individually at the time of randomization by the LPN  **Group 2 Intervention:** Remote comprehensive diabetes self-management education (DSME) intervention, Diabetes TeleCare, administered by a dietitian and nure/certified diabetes educator (CDE) Three group sessions were conducted in-person; all others were conducted by interactive videoconferencing by the self-management education team Additionally, participants in the intervention group were offered retinal imaging in the primary-care setting when they were due for their annual exam | **Mean age group 1:** 59 (SD 9.300)  **Mean age group 2:** 59.9 (SD 9.4)  **% male group 1:** 23.7  **% male group 2:** 27.1  **Ethnicity group 1:** Not reported | **Primary outcome:**  HbA1c SIG  **p =** 0.004 |
| {Godwin, 2009 #66} | **Study design:** RCT **CCM elements:** CIS+DSD **Chronic condition:** Hypertension  **Country:** Canada **Setting:** Primary care **Duration of intervention in (months):** 12.0  **Number of patients:** 285.0 **Number of health professionals:** 24.0 **Study Aim:** The rationale for conducting our study was based on the belief that in the family practice environment, hypertensive patients with above target BP would improve their BP control if they monitored their BP at home. | **Group 1 Intervention:**  Canadian hypertension guidelines  **Group 2 Intervention:** Patients were divided into two groups: one with at least weekly measurements of BP at home, recording those measurements and showing those to the family physician during office visits for hypertension and the control group were given Usual care Home BP monitoring device and instructed in the proper use of the device | **Mean age group 1:** 69 (SD 11.8)  **Mean age group 2:** 67.8 (SD 11.6)  **% male group 1:** 48.7  **% male group 2:** 48.4  **Ethnicity group 1:** Not reported | **Primary outcome:**  SBP NS  **p =** |
| {Koelewijn-van Loon, 2009 #88} | **Study design:** RCT **CCM elements:** DSD+SMS+DS **Chronic condition:** Heart disease  **Country:** Netherlands **Setting:** Primary care **Duration of intervention in (months):** 12.0  **Number of patients:** 615.0 **Number of health professionals:** **Study Aim:** We examined the effect of involving patients in nurse-led cardiovascular risk management on lifestyle adherence and cardiovascular risk. | **Group 1 Intervention:**  In both groups, general practitioners delegated the tast of cardiovascular risk management to the practice nurses  **Group 2 Intervention:** Nurses informed the patients of their absolute 10-year cardiovascular mortality risk using a risk communication tool developed for this study. Nurse provided support to patients using an updated decision aid. Adapted motivational intverviewing. The nurses consulted with the general practitioners if medication was being considered | **Mean age group 1:** 58 (SD 10)  **Mean age group 2:** 56.0 (SD 10)  **% male group 1:** 47.0  **% male group 2:** 43.0  **Ethnicity group 1:** Not reported | **Primary outcome:**  Lifestyle adherece (composite adherence score) NS  **p =** |
| {Krass, 2007 #89} | **Study design:** RCT **CCM elements:** DSD+SMS **Chronic condition:** Diabetes  **Country:** Australia **Setting:** Community-based care **Duration of intervention in (months):** 6.0  **Number of patients: Number of health professionals:** **Study Aim:** To assess the impact of a community pharmacy diabetes service model on patient outcomes in Type 2 diabetes. | **Group 1 Intervention:**  Usual care  **Group 2 Intervention:** Intervention pharmacies delivered diabetes service to patients with type 2 diabteres, which comprised an ongoing cycle of assessment, management and review provided at regular intervals over 6 months in the pharmacy. Services included support for self monitoring of blood glucose, education, addherence support, and remiders of checks for diabetes complications | **Mean age group 1:** 62 (SD 11)  **Mean age group 2:** 62.0 (SD 11)  **% male group 1:** 51.0  **% male group 2:** 51.0  **Ethnicity group 1:** Not reported | **Primary outcome:**  HbA1c SIG  **p =** 0.020 |
| {Shea, 2009 #140} | **Study design:** RCT **CCM elements:** DSD+SMS+DS **Chronic condition:** Diabetes  **Country:** USA **Setting:** Mixed **Duration of intervention in (months):** 60.0  **Number of patients:** 1665.0 **Number of health professionals:** **Study Aim:** To examine the effectiveness of a telemedicine intervention to achieve clinical management goals in older, ethnically diverse, medically underserved patients with diabetes. | **Group 1 Intervention:**  Usual care  **Group 2 Intervention:** IDEATel - home telemdeicne unit with nurse case management of diabetes | **Mean age group 1:** 71 (SD 6.8)  **Mean age group 2:** 70.8 (SD 6.5)  **% male group 1:** 37.9  **% male group 2:** 36.5  **Ethnicity group 1:** Caucasian | **Primary outcome:**  HbA1c SIG  **p =** 0.001 |
| {McManus, 2014 #108} | **Study design:** RCT **CCM elements:** SMS+CIS **Chronic condition:** Hypertension  **Country:** UK **Setting:** Primary care **Duration of intervention in (months):** 12.0  **Number of patients:** 552.0 **Number of health professionals:** **Study Aim:** To determine the effect of self-monitoring with self-titration of antihypertensive medication compared with usual care on systolic blood pressure among patients with cardiovascular disease, diabetes, or chronic kidney disease | **Group 1 Intervention:**  Usual care  **Group 2 Intervention:** Patients randomized to self-management were trained to self-monitor blood pressure using a validated monitor with self-titration of medication following a predetermined plan, in 2 or 3 sessions, each lasting aproximately an hour. Following training, intervention patients went to their family physician to agree with the individualized 3-step plan to increase of add antihypertensive medications. | **Mean age group 1:** 70 (SD 9.699)  **Mean age group 2:** 69.3 (SD 9.300)  **% male group 1:** 59.4  **% male group 2:** 60.1    **Ethnicity group 1:** Caucasian | **Primary outcome:**  SBP SIG  **p =** |
| {Ogedegbe, 2014 #119} | **Study design:** RCT **CCM elements:** SMS+DSD+DS **Chronic condition:** Hypertension  **Country:** USA **Setting:** Mixed **Duration of intervention in (months):** 12.0  **Number of patients:** 1039.0 **Number of health professionals:** **Study Aim:** Using the chronic care model as an implementation framework, the Counseling African Americans to Control Hypertension (CAATCH) trial used a cluster-randomized design to evaluate the effectiveness of a practice-based, multilevel intervention for improving BP control among hypertensive patients. The intervention targeted both physicians and patients in CHCs. | **Group 1 Intervention:**  Patients and physicians at the Usual care (UC) site received printed patient education material and hypertension treatment guidelines, respectively  **Group 2 Intervention:** 6 behavioral lifestyle telephone/group couseling sessions 4 modules of interactive, computerized patient education focused on the causes, complications, and treatment of HTN; expected medication adverse events; and methods for adoption of healthy lifestyle behaviors. Free validated automated home BP monitors. They were encouraged to record their weekly BP readings in a diary. Physicians attended monthly hypertension case rounds and received feedback on their patients' home BP readings and chart audits | **Mean age group 1:** 57 (SD 12.8)  **Mean age group 2:** 56.3 (SD 11.5)  **% male group 1:** 26.0  **% male group 2:** 30.7  **Ethnicity group 1:** African American | **Primary outcome:**  BP NS  **p =** |
| {Harris, 2013 #74} | **Study design:** RCT **CCM elements:** DS+DSD **Chronic condition:** Diabetes  **Country:** Canada **Setting:** Mixed **Duration of intervention in (months):** 12.0  **Number of patients: Number of health professionals:** 154.0 **Study Aim:** The objective of the Advancing Insulin Management in General Practice (AIM@GP) trial was to determine the effectiveness of an insulin initiation strategy utilizing diabetes specialist and community retail pharmacy support to increase family physician insulin prescribing rates | **Group 1 Intervention:** Physicians received community retail pharmacist support (option to refer patients to pharmacist(s) for a 1-hour insulin-initiation session) Family physicians received insulin initiation/titration education, a physician-specific 'report card' on the characteristics of their type 2 diabetes (T2DM) population, and a registry of insulin-eligible patients at a workshop  **Group 2 Intervention:**  Usual care | **Mean age group 1:** 51.4 (SD 8.81 )  **Mean age group 2:** 48.2 (SD 9.06 )  **% male group 1:** 72.6  **% male group 2:** 75.6  **Ethnicity group 1:** Not reported | **Primary outcome:**  Physician's insulin prescribing rate (IPR) - The number of insulin starts per 12- month intervention of insulin eligible patients NS  **p =** |
| {Mitchell, 2014 #109} | **Study design:** RCT **CCM elements:** SMS+DSD **Chronic condition:** COPD  **Country:** UK **Setting:** Primary care **Duration of intervention in (months):** 6.0  **Number of patients:** 184.0 **Number of health professionals:** **Study Aim:** The Self-Management Programme of Activity, Coping and Education (SPACE) FOR COPD is a 6-week self-management intervention for COPD, and this study aimed to evaluate the effectiveness of this intervention in primary care. | **Group 1 Intervention:**  Usual care  **Group 2 Intervention:** The comprehensive programme is structured around the SPACE FOR COPD manual, which is a 176-page workbook that individuals can follow independently at home. Participants randomised to SPACE FOR COPD were introduced to the programme by a physiotherapist during a 30-45-min consultation. Motivational interviewing technique were used to underpin the cosultation | **Mean age group 1:** 69 (SD 10.1)  **Mean age group 2:** 69.0 (SD 8)  **% male group 1:** 49.0  **% male group 2:** 60.0  **Ethnicity group 1:** Caucasian | **Primary outcome:**  The primary outcome was symptom burden, measured by the self-reported Chronic Respiratory Questionnaire (CRQ) dsypnoea domain NS  **p =** |
| {Niiranen, 2013 #116} | **Study design:** RCT **CCM elements:** SMS+DS+DSD **Chronic condition:** Hypertension  **Country:** Finland **Setting:** Primary care **Duration of intervention in (months):** 12.0  **Number of patients:** 229.0 **Number of health professionals:** **Study Aim:** The implementation of lifestyle modifications, home blood pressure (BP) measurement, and optimization of antihypertensive drug therapy have been shown to improve BP control in tightly controlled research settings. Our objective was to determine the effect of these interventions in a primary care setting, with the family practitioners and nurses serving as the interventionists. | **Group 1 Intervention:**  Usual care  **Group 2 Intervention:** Individual and group couseling from trained GP or nurse of the health center on healthy lifestyle. Their antihypertensive drug therapy was guided by home BP measurements performed at 3-month intervals instead of by conventional office measurements. Participants self-measured their BP at 0,3,6 and 12 months from the beginning of the study and additionally 1 month after any changes in antihypertensive medication. The BP readings were mailed to the treating physician | **Mean age group 1:** 62 (SD 9.1)  **Mean age group 2:** 62.9 (SD 8)  **% male group 1:** 51.8  **% male group 2:** 48.1  **Ethnicity group 1:** Not reported | **Primary outcome:**  SBP NS  **p =** |
| {Kruis, 2014 #90} | **Study design:** RCT **CCM elements:** DS+DSD+SMS **Chronic condition:** COPD  **Country:** Netherlands **Setting:** Primary care **Duration of intervention in (months):** 24.0  **Number of patients:** 1086.0 **Number of health professionals:** **Study Aim:** To investigate the long term effectiveness of integrated disease management delivered in primary care on quality of life in patients with chronic obstructive pulmonary disease (COPD) compared with usual care. | **Group 1 Intervention:**  The practice nurses in the Usual care group received a course on clinical performance of spirometry only, to divert attention from topics related to the intervention  **Group 2 Intervention:** General practitioners, practice nurses, and specialised physiotherapists in the intervention group received a two day training course on incorporating integrated disease management in practice. During a refresher course at 6 and 12 months, the team learnt the details of a web-based decision support system for audit and feedback with patients' and professionals' portals, named Zorgdraad **.** | **Mean age group 1:** 68 (SD 11.1)  **Mean age group 2:** 68.2 (SD 11.3)  **% male group 1:** 57.3  **% male group 2:** 50.5  **Ethnicity group 1:** Not reported | **Primary outcome:**  The primary outcome was health status at 12 months, measured by the Clinical COPD Questionnaire (CCQ) NS  **p =** |
| {Griffin, 2011 #70} | **Study design:** RCT **CCM elements:** DS+DSD+SMS **Chronic condition:** Diabetes  **Country:** Mixed **Setting:** Primary care **Duration of intervention in (months):** 60.0  **Number of patients:** 3057.0 **Number of health professionals:** **Study Aim:** Intensive treatment of multiple cardiovascular risk factors can halve mortality among people with established type 2 diabetes. We investigated the effect of early multifactorial treatment after diagnosis by screening. | **Group 1 Intervention:**  Routine care group (According to guidelines of Dutch College of General Practitioners)  **Group 2 Intervention:** A treatment protocol: intensive treatment of glucose, blood pressure and lipids and structured lifestyle education. The intensive treatment protocol was carried out by a diabetes nurse together with a GP. Structured lifestyle education. Nurses were authorized to prescribe medications, supervised by GPs. During the first year of intervention, every 3 months a 2 hours training session was arranged. The sessions included discussions about obstacles to reaching target values, exchanging experiences, and evaluating cooperation with GPs. At least once a year, GPs in ADDITION group were reminded to treat their patients according to protocol **.** | **Mean age group 1:** 60 (SD 6.8)  **Mean age group 2:** 60.3 (SD 6.9)  **% male group 1:** 57.3  **% male group 2:** 58.5  **Ethnicity group 1:** Caucasian | **Primary outcome:**  Composite cardiovascular events NS  **p =** |
| {Villeneuve, 2010 #174} | **Study design:** RCT **CCM elements:** DSD+DS **Chronic condition:** Lipid disorders  **Country:** Canada **Setting:** Mixed **Duration of intervention in (months):** 12.0  **Number of patients:** 225.0 **Number of health professionals:** 185.0 **Study Aim:** In this study, we compared a collaborative model involving physicians and pharmacists with usual care for patients with dyslipidemia | **Group 1 Intervention:**  Usual care  **Group 2 Intervention:** The pharmacist provided couseling and used a patient decision aid to draw up a treatment plan, which included lifestyle changes and pharmacotherapy. During the titration visits, the pharmacist evaluated lifestyle changes, the patient's tolerance of and adherence with the pharmacotherapy, and drug's efficacy and then adjusted the statin dosage accordingly. After each visit, the phramacist prepared an interim report and sent it to the physician by fax | **Mean age group 1:** 62 (SD 12)  **Mean age group 2:** 59.3 (SD 9.6)  **% male group 1:** 60.0  **% male group 2:** 64.0  **Ethnicity group 1:** Not reported | **Primary outcome:**  Lipids NS  **p =** |
| {Serumaga, 2011 #138} | **Study design:** ITS **CCM elements:** HCO **Chronic condition:** Hypertension  **Country:** UK **Setting:** Primary care **Duration of intervention in (months):**  **Number of patients: Number of health professionals:** **Study Aim:** To assess the impact of a pay for performance incentive on quality of care and outcomes among UK patients with hypertension in primary care. | **Group 1 Intervention:**  Based on the proportion of patients achieving certain quality indicators, general practitioners could receive payments as high as 25% of their total income | **Mean age group 1:** 58 (SD 15.4)  **% male group 1:** 44.8  **Ethnicity group 1:** Not reported | **Primary outcome:**  BP NS  **p =** |
| {Simpson, 2010 #144} | **Study design:** RCT **CCM elements:** DSD+DS **Chronic condition:** Hypertension  **Country:** Canada **Setting:** Mixed **Duration of intervention in (months):** 12.0  **Number of patients:** 260.0 **Number of health professionals:** **Study Aim:** To evaluate the effect of adding pharmacists to primary care teams on the management of hypertension and other cardiovascular risk factors in patients with type 2 diabetes. | **Group 1 Intervention:**  Usual care  **Group 2 Intervention:** Pharmacists performed medication assessments and limited history and physical examinations and provided guideline-concordant recommendations to optimize medication management. These recommendations were discussed with the primary care physician who was responsible for authorizing medication changes. The pharmacist then worked independently with the patient to implement these changes | **Mean age group 1:** 59 (SD 12.1)  **Mean age group 2:** 58.8 (SD 11.1)  **% male group 1:** 41.9  **% male group 2:** 43.5  **Ethnicity group 1:** Not reported | **Primary outcome:**  SBP SIG  **p =** 0.020 |
| {Jansink, 2013 #80} | **Study design:** RCT **CCM elements:** SMS+DSD+DS **Chronic condition:** Diabetes  **Country:** Netherlands **Setting:** Primary care **Duration of intervention in (months):** 14.0  **Number of patients:** 940.0 **Number of health professionals:** **Study Aim:** To study the effectiveness of a comprehensive diabetes programme in general practice that integrates patient-centred lifestyle counselling into structured diabetes care. | **Group 1 Intervention:**  Usual care  **Group 2 Intervention:** Nurses in the intervention group received a comprehensive programme Training in lifestyle couseling based on motivational interviewing Record-keeping and instruction chart Feedback about their own video-recording | **Mean age group 1:** 64 (SD 9.800)  **Mean age group 2:** 64.1 (SD 8.9)  **% male group 1:** 54.0  **% male group 2:** 55.9  **Ethnicity group 1:** Not reported | **Primary outcome:**  HbA1c NS  **p =** |
| {Munshi, 2012 #112} | **Study design:** RCT **CCM elements:** SMS+DSD **Chronic condition:** Diabetes  **Country:** USA **Setting:** Mixed **Duration of intervention in (months):** 12.0  **Number of patients:** 100.0 **Number of health professionals:** **Study Aim:** To evaluate whether assessment of barriers to self-care and strategies to cope with these barriers in older adults with diabetes is superior to usual care with attention control. The American Diabetes Association guidelines recommend the assessment of age-specific barriers. However, the effect of such strategy on outcomes is unknown. | **Group 1 Intervention:**  An educator called participants a total of 11 times within the first 6 months. Did not provide any diabetes-related advice or strategies and only discussed non-diabetes-related life events  **Group 2 Intervention:** Patients underwent evaluation for barriers to self-care by a diabetes educator we versed with age-specific barriers. The strategies were designed to optimize patients' ablility to prefrom self-care leading to better adherence with treatment recommendation given by their medical providers. Patients in this group received phone contact from the care managers as often as needed during the intervention period | **Mean age group 1:** 75 (SD 5)  **Mean age group 2:** 75.0 (SD 5)  **% male group 1:** 53.0  **% male group 2:** 43.0  **Ethnicity group 1:** Not reported | **Primary outcome:**  HbA1c SIG  **p =** 0.030 |
| {Nguyen, 2013 #114} | **Study design:** RCT **CCM elements:** SMS+DSD+CIS **Chronic condition:** COPD  **Country:** USA **Setting:** Community-based care **Duration of intervention in (months):** 12.0  **Number of patients:** 125.0 **Number of health professionals:** **Study Aim:** The purpose of this study was to test the efficacy of two 12-month dyspnea self-management programs (DSMPs), Internet-based (eDSMP) and face-to-face (fDSMP), compared with a general health education (GHE) control on the primary outcome of dyspnea with activities. | **Group 1 Intervention:**  Attention control  **Group 2 Intervention:** Internet-based Dyspnea Self-Management Program (eDSMP). The eDSMP incorporated technological enhancements to support earlier recognition of worsening symptoms through real-time monitoring, prompt feedback, and convenient access to information and support. Consultation by a nurse was to establish rapport with participants and understand his or her current level of exercise and experiences with dyspnea through motivational interviewing **Group 3 Intervention:** Face-to-face Dyspnea Self-Management Program (fDSMP). Consultation by a nurse was to establish rapport with participants and understand his or her current level of exercise and experiences with dyspnea through motivational interviewing Individualized feedback and reinforcement regarding use of dyspnea self-management strategies and exercise progress vie e-mail (eDSMP) or telephone (fDSMP) | **Mean age group 1:** 69 (SD 8)  **Mean age group 2:** 68.5 (SD 11)  **Mean age group 3:** 68.2 (SD 9.9)  **% male group 1:** 59.0  **% male group 2:** 58.0  **% male group 3:** 46.0  **Ethnicity group 1:** Caucasian | **Primary outcome:**  Dyspnea with activities NS  **p =** |
| {Dresser, 2013 #45} | **Study design:** RCT **CCM elements:** DS **Chronic condition:** Hypertension  **Country:** Canada **Setting:** Primary care **Duration of intervention in (months):** 6.0  **Number of patients:** 1272.0 **Number of health professionals:** **Study Aim:** We questioned whether a simplified comprehensive treatment algorithm featuring initial use of single-pill combinations (SPCs) would improve management of participants with both hypertension and dyslipidemia. | **Group 1 Intervention:**  Guideline care  **Group 2 Intervention:** STITCH2 algorithm Investigators available to family practitioners for consultation | **Mean age group 1:** 61 (SD 11)  **Mean age group 2:** 61.6 (SD 11.5)  **% male group 1:** 53.8  **% male group 2:** 53.5  **Ethnicity group 1:** Not reported | **Primary outcome:**  BP+Lipids NS  **p =** |
| {Eakin, 2013 #49} | **Study design:** RCT **CCM elements:** SMS+DSD **Chronic condition:** Diabetes  **Country:** Australia **Setting:** Primary care **Duration of intervention in (months):** 6.0  **Number of patients:** 302.0 **Number of health professionals:** **Study Aim:** Living Well with Diabetes is a telephone-delivered weight loss intervention designed for real-world delivery | **Group 1 Intervention:**  Usual care  **Group 2 Intervention:** The intervention followed a motivational interviewing approach. Detailed workbook. Telephone intervention | **Mean age group 1:** 58 (SD 9)  **Mean age group 2:** 57.7 (SD 8.1)  **% male group 1:** 57.0  **% male group 2:** 55.6  **Ethnicity group 1:** Caucasian | **Primary outcome:**  Weight SIG  **p =** 0.006 |
| {Juul, 2014 #84} | **Study design:** RCT **CCM elements:** DS+DSD+SMS **Chronic condition:** Diabetes  **Country:** Denmark **Setting:** Primary care **Duration of intervention in (months):** 18.0  **Number of patients: Number of health professionals:** **Study Aim:** The aim of this trial was to evaluate the effectiveness of a training course for general practice nurses in motivation support at 18 months follow-up in the affiliated type 2 diabetes population | **Group 1 Intervention:**  Usual care  **Group 2 Intervention:** 16-hour course with interactive training targeted the nursed employed in the practice. Self-determination theory-based course 30-minute visit to the practice by one of the course teachers (month 10), during which implementation issures were addressed | **Mean age group 1:** 61 (SD 8.6)  **Mean age group 2:** 60.2 (SD 8.5)  **% male group 1:** 57.1  **% male group 2:** 55.9  **Ethnicity group 1:** Not reported | **Primary outcome:**  HbA1c NS  **p =** |
| {McKinstry, 2013 #105} | **Study design:** RCT **CCM elements:** SMS+DS+CIS **Chronic condition:** Hypertension  **Country:** UK **Setting:** Primary care **Duration of intervention in (months):** 6.0  **Number of patients:** 401.0 **Number of health professionals:** **Study Aim:** To determine if an intervention consisting of telemonitoring and supervision by usual primary care clinicians of home self measured blood pressure and optional patient decision support leads to clinically important reductions in daytime systolic and diastolic ambulatory blood pressure in patients with uncontrolled blood pressure. | **Group 1 Intervention:**  Usual care  **Group 2 Intervention:** 20 minute training session on how to use telemonitoring. Participants and clinicians could log on to a website to see the data, and automated SMS texts or emails could be send to participants with feedback on their blood pressure control. Participants could contact their clinicians if they were concerned about their blood pressure control and clinicians could contact participants if needed to arrange modification of therapy. Educational sessions with a member of the research team who specialises in the management of hypertension | **Mean age group 1:** 61 (SD 10.7)  **Mean age group 2:** 60.5 (SD 11.8)  **% male group 1:** 60.0  **% male group 2:** 59.0  **Ethnicity group 1:** Not reported | **Primary outcome:**  SBP SIG  **p =** 0.000 |
